# The Homeobox Genes: Classification, Regulation, Biological Functions, and Diseases

**DOI:** 10.1002/mco2.70651

**Published:** 2026-03-16

**Authors:** Maedeh Dadzadi, Shahin Ramazi, Mona Darvazi, Sepideh Yoosefi, Melika Abbasi, Shirin Farsad

**Affiliations:** ^1^ Department of Biotechnology Faculty of Advanced Science and Technology Tehran Medical Sciences Islamic Azad University Tehran Iran; ^2^ Department of Biophysics Faculty of Biological Sciences Tarbiat Modares University Tehran Iran; ^3^ Department of Drug and Food Control Faculty of Pharmacy Tehran University of Medical Sciences Tehran Iran; ^4^ Department of Biotechnology Medical Islamic Azad University of Tehran Tehran Iran; ^5^ Department of Microbiology Faculty of Basic Science Qom Branch Islamic Azad University Qom Iran

**Keywords:** cancer, epigenetics, homeobox genes, lung cancer, noncancerous diseases, pathological variants

## Abstract

Homeobox genes constitute a large family of transcription factors that act as master regulators involved in multiple fundamental processes such as development and cell differentiation. Consequently, these transcription factors perform diverse functions throughout human life. However, dysregulation of homeobox gene expression, through pathogenic variants or epigenetic alterations, has been increasingly associated with a wide range of human disorders. In particular, correlations between homeobox genes and various types of cancer have been documented in hundreds of studies. This review provides an integrative overview of homeobox gene biology, summarizing their classification as well as their physiological and pathological roles across noncancerous and cancerous diseases. Particular attention is given to how dysregulation of gene expression contributes to various noncancerous diseases (e.g., congenital, metabolic, and neurodegenerative disorders) and to malignancies, especially the five highest incidence of cancers, with a detailed focus on lung cancer, where epigenetic mechanisms play a central role in tumor progression.

## Graphical abstract

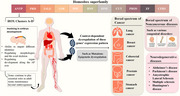

## 1 Introduction

It has been four decades since researchers first identified a short but pivotal DNA sequence, later termed the homeobox. Across the animal kingdom, homeobox genes give rise to a broad class of transcription factors (TFs) that play central roles in developmental pathways across tissues and stages of life. The initial discovery of these genes was made in *Drosophila melanogaster*, where they were found to regulate homeotic transformations, defining the identity of body segments [1, 2, 3, 4]. Since then, hundreds of homeobox‐containing genes have been identified in a wide range of species. Homeobox genes constitute one of the largest TFs superfamilies in the human genome, with over 200 members characterized to date. Among vertebrates, the HOX gene family is one of the best‐studied subsets of the homeobox gene superfamily [5, 6]. This homeobox genes have conserved region, approximately 180 base pairs long, that encodes a ∼60‐amino‐acid DNA‐binding domain known as the homeodomain (HD). Therefore, a defining feature of most homeobox genes is their conserved HD, which enables sequence‐specific DNA binding and precise regulation of gene expression essential for embryogenesis and cell differentiation. Accordingly, numerous studies have established that TFs encoded by homeobox genes serve as master regulators of developmental processes, functioning at the top of gene regulatory hierarchies. Through this hierarchical control, they initiate broad genetic cascades that govern the expression of numerous downstream genes (e.g., effector genes), eventually contributing to the formation of tissue and organ [3, 7, 8, 9]. In particular, *HOX* genes encode evolutionarily conserved TFs that are indispensable for the proper development of bilaterian body plans. Notably, more posterior body regions typically express a larger complement of *HOX* genes than do anterior regions. This spatio‐temporal collinearity of *HOX* genes expression highlights how genomic regulation underpins their role as master regulators in developmental patterning [7]. Importantly, some homeobox genes, chiefly those within the HOX clusters, remain transcriptionally active well beyond embryogenesis. Their region‐specific expression is stably preserved in adult cell types, such as mesenchymal stem cells (SCs) and fibroblasts, where it forms an epigenetically maintained “positional memory” that preserves in embryonic axial information and continues to shape tissue physiology across the lifespan [10, 11, 12, 13]. High‐resolution transcriptomic profiling of fibroblasts isolated from anatomically precise sites confirms that these cells retain a distinctive “HOX code” (the position specific‐pattern of *HOX* genes’ expression), mirroring the embryonic pattern of these genes’ expression well into adulthood [11, 13, 14].

Despite their well‐established developmental roles, growing evidence indicates that homeobox gene dysregulation underlies a wide spectrum of human pathologies, encompassing both noncancerous diseases and multiple cancer types. Alterations in homeobox gene dosage, structure, or transcriptional regulation have also been implicated in diverse noncancerous disorders, ranging from congenital malformations and metabolic syndromes to neurodegenerative conditions, as well as in cancer, where their dysregulation contributes directly to tumorigenesis [15, 16]. Decades of research have demonstrated that aberrant expression of specific homeobox genes promotes tumor invasion, metastasis, and poor prognosis, frequently correlating with adverse clinicopathological features and poorer survival across tumor types [3, 17, 18]. Notably, accumulating studies reveal that mechanisms such as aberrant DNA methylation, overexpression, and regulation of expression by long noncoding RNAs (lncRNAs)/microRNAs (miRNAs) play critical roles in modulating homeobox gene activity and, consequently, influence oncogenic signaling pathways. Evidence increasingly supports that homeobox function is highly context dependent, with the same gene exerting opposing effects depending on the cell type, tissue microenvironment, and interacting molecular networks [19].

Building on these insights, the present review aims to provide a comprehensive and integrative synthesis of homeobox gene biology across physiological and pathological contexts. Specifically, this review (i) systematically reviews homeobox gene expression and function in normal physiology, emphasizing their hierarchical control of differentiation and tissue homeostasis; (ii) delineates the genetic and epigenetic mechanisms through which homeobox dysregulation contributes to disease pathogenesis across congenital, systemic, and neurodegenerative disorders; (iii) reviews the role of homeobox genes in malignancies, with particular focus on the five highest incidence of human cancers lung, breast, colorectum, prostate, and stomach with an in‐depth discussion of lung cancer, where homeobox deregulation is especially prominent; and (iv) explores unifying mechanistic themes linking homeobox gene regulation, tissue‐specific function, and disease etiology.

## 2 Homeobox Genes

### 2.1 Classification of Homeobox Genes

**FIGURE 1:**
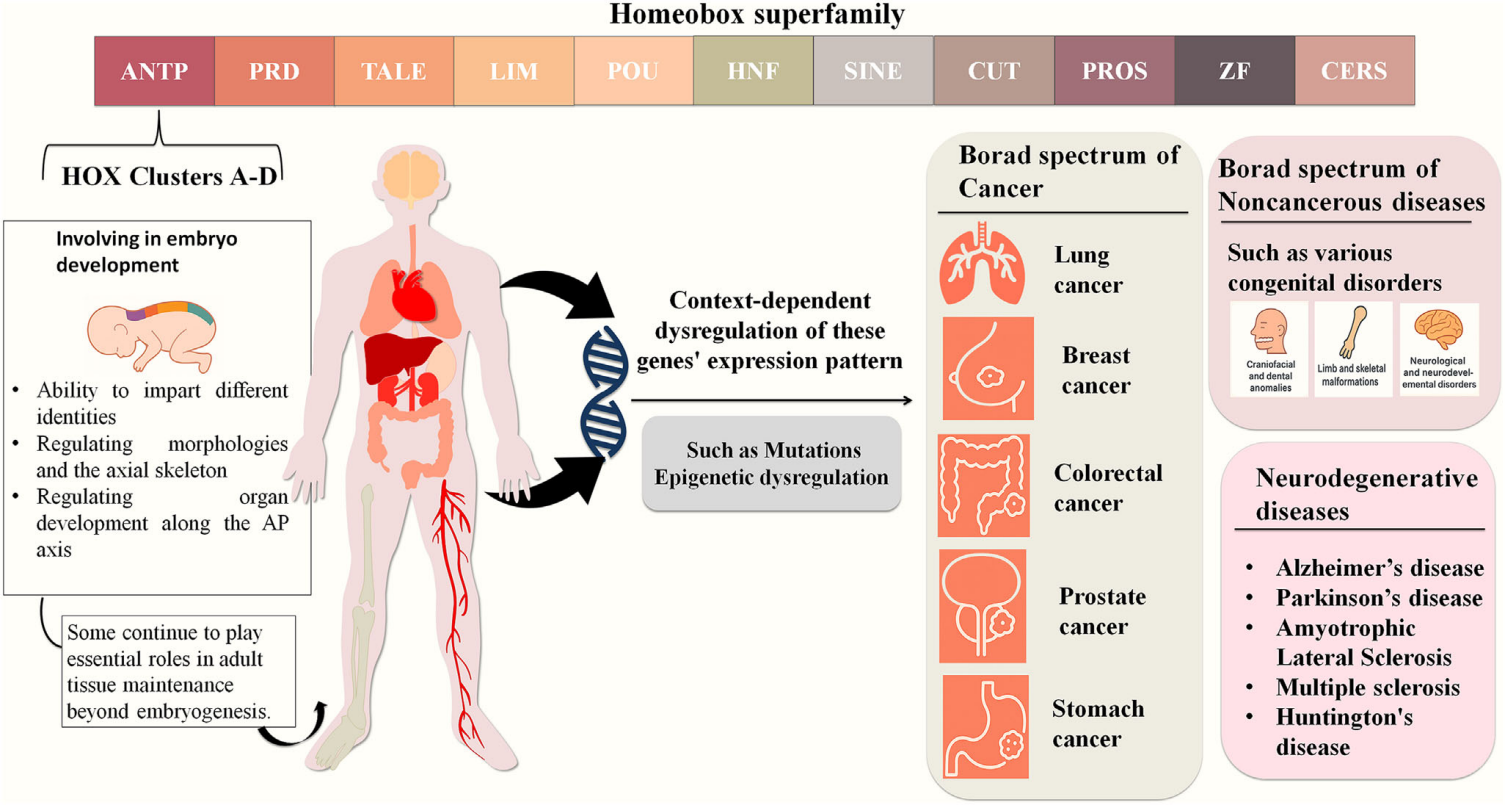
Overview of the homeobox gene superfamily and its pathophysiological roles. The homeobox superfamily comprises several major classes, including *ANTP*, *PRD*, *TALE*, *LIM*, *POU*, and others. Among these, the HOX clusters (A–D) play critical roles in embryonic development specifically in conferring cellular identity, regulating morphogenesis, and guiding axial patterning. Beyond development, *HOX* genes remain active in adult tissue homeostasis. Dysregulation of their expression, whether through mutation or epigenetic alteration, is implicated in a broad spectrum of diseases, including multiple cancers and neurodegenerative disorders.

Homeobox genes have been classified into distinct categories by generally examining both the evolutionary relationships of their homeodomain amino acid sequences and the association of the homeodomain with additional protein domains [20]. Another important criterion for classification of homeobox genes into specific families involves assessing their sequence similarity, as genes with higher degrees of homology are typically placed within the same family [21, 22]. In the human genome, homeobox genes are broadly categorized into 11 core classes, each further subdivided into subclasses comprising multiple gene families (Figure 1 and Table 1). Within these families, individual genes share conserved sequences and structural domains that reflect their functional and evolutionary relationships. It should be noted that members of the Antennapedia (ANTP) class are restricted exclusively to multicellular animals (Metazoa) and have not been identified in unicellular eukaryotes, even though homeobox genes do exist in the latter. In humans, the *ANTP* class constitutes the most expansive and functionally versatile category within the homeobox gene superfamily. It is subdivided into two primary subclasses: the *HOX‐like* (*HOXL*) genes, most notably the *HOX* genes, and the NK‐like (NKL) genes, which are classified as non‐HOX. Beyond the well‐known *HOX* clusters, the *HOXL* subclass also includes other key members, such as the ParaHox genes (e.g., *caudal‐type homeobox* (*CDX*), and *genomic screened homeobox* (*GSX*)) and a group of extended *HOX* genes [7, 20, 23, 24]. Among these, the ParaHox genes stand out not only for their developmental significance but also for their evolutionary origin. The term “ParaHox cluster” was introduced to reflect its sequence similarity and evolutionary correspondence to specific paralogous groups within the HOX gene clusters. Beyond their structural complexity, the evolutionary trajectories of HOX and ParaHox genes offer key insights into the ancient origins of the ANTP class. Comparative genomic analyses suggest that the remarkable diversification of this class arose through early tandem gene duplication events. These duplications are thought to have produced distinct lineages, including the NK subclass and a putative ProtoHox cluster. The latter is proposed to have undergone further duplication prior to the evolutionary split between deuterostomes and protostomes, ultimately giving rise to both the HOX and ParaHox clusters. This functional compartmentalization reflects the evolutionary refinement of ANTP‐derived clusters toward distinct developmental trajectories [23, 25].

**TABLE 1:** Functional, family‐level overview of selected human homeobox genes. The table lists representative protein‐coding genes. Notably, within the *ANTP* class, two major subclasses can be distinguished: HOXL and NKL. For more detailed comprehensive overview, see the review article from Holland et al. [20].

Class	Subclass	Family/representative genes	Core developmental processes (examples)	References
ANTP (Antennapedia)	HOXL	HOX (A–D)	Anterior–posterior patterning, and involve in the development of axial skeleton, hindbrain, and hematopoietic	[7]
		EVX (*EVX1*)	*EVX1* is expressed in the posterior primitive streak, occurring earlier in comparison with other genes within the HOXA cluster.	[26]
		MNX (*MNX1*)	*MNX1* is marker and specification of the spinal MN lineage.	[27]
		MEOX (*MEOX1/2*)	*MEOX1* regulates the development of somite.	[28]
		GBX (*GBX1/2*)	*GBX1* and *2* regulate hindbrain formation and MHB morphogenesis.	[29]
		PDX (*PDX1*)	*PDX1* regulates pancreatic organogenesis and β‐cell identity.	[30]
		CDX (*CDX1/CDX2*)	*CDX2* development and differentiation of intestinal epithelial cells.	[31]
	NKL	BARHL (*BARHL1*)	*BARHL1* regulates inner ear hair‐cell and CNS sensory neuron development through Atoh1‐dependent, autoregulatory expression.	[32]
		BARX (*BARX1*)	*BARX1* expression involves in the development of face, cranium, muscle, and stomach.	[33]
		DLX (*DLX 1–6*)	*DLX5* involves in the regulation of limb development.	[34]
		MSX (*MSX1/2*)	*MSX1/2* are expressed in embryonic craniofacial mesenchyme; *MSX1* acts as a transcriptional repressor essential for odontogenesis and palatogenesis.	[35]
		NKX (*NKX2‐5*)	*NKX2‐5* is involved in cardiac development, acting as one of the earliest markers of cardiac progenitors and being essential for cardiac morphogenesis and postnatal heart function.	[36]
		HMX (*HMX1/2/3*)	*HMX1* contributes to the regulation of craniofacial and somatosensory development; it is highly expressed in the eye and branchial arches and influences neuronal fate and migration.	[37]
		LBX (*LBX1*)	*LBX1* is essential for the development of nerve tissue and muscles.	[38]
		VAX (*VAX1/2*)	*VAX1* contributes to optic chiasm development via RGC regulation.	[39]
		VENTX	*VENTX* expression drives myeloid differentiation.	[40]
		HHEX	*HHEX* expression contributes to normal liver and bile‐duct development and to the establishment of hematopoiesis.	[41]
		NOTO	*NOTO* contributes to a proper node morphogenesis and regulates ciliogenesis in the posterior notochord.	[42]
PRD (Paired)	PAX	*PAX1/ PAX9*	*PAX1* and *PAX9* cooperatively regulate sclerotome cells patterning and differentiation into vertebral bodies and IVD.	[43]
	PAXL	OTX (*OTX1/2*)	*OTX* genes regulate the development of sensory organs, the early fetal retina, and the mammary gland.	[44]
		PITX (*PITX1/2/3*)	*PITX* regulates left–right asymmetry and the development of various organs, such as the eye, brain, pituitary, branchial arches, and hindlimbs.	[45]
		SHOX (*SHOX2*)	*SHOX2* is implicated in heart development and its expression occurs in CNS basal plate, cardiac inflow tract, third pharyngeal arch, and derived structures.	[46, 47]
		ALX (*ALX1–3*)	*ALX1* expression in frontonasal neural crest cells contributes to periocular and frontonasal mesenchyme.	[48]
		PHOX (*PHOX2A*)	*PHOX2A* is crucial for determining the noradrenergic phenotype and exhibits nervous system‐specific functions.	[49, 50]
		HESX (*HESX1*)	*HESX1* is expressed during early forebrain and pituitary development	[51]
		*RHOX (RHOX1, RHOX6, RHOX8*, and *RHOX10)*	The expression of *RHOX* genes is implicated in germ‐cell development.	[52]
	PAXL (embryo‐restricted)	*ARGFX, CPHX (CPHX1/2), DPRX, DUXA, DUXB, NOBOX*, and *TPRX (TPRX1/2)*	These genes are predominantly expressed during human preimplantation embryonic stages.	[53]
TALE	*—*	IRX (*IRX1–6*)	*IRX* genes participate in both development and differentiation.	[54]
		MEIS (*MEIS1–3*)	*MEIS2* regulates the differentiation of hESCs into various types of cardiovascular cells.	[55]
		Mohawk (*MKX*)	*MKX* maintains tendon/ligament homeostasis and mediates mechanotransduction in tenocytes.	[56]
		PBX (*PBX1–4*)	*PBX1* is involved in coordinating multiorgan patterning, including skeletal and limb structures, cardiovascular and neuronal systems, and the development of the hematopoietic, pancreas, and urogenital tract.	[57]
		*PREP (PREP1/2*, also referred as *PKNOX1)*	*PREP1* is critically required for normal embryonic development.	[58]
		TGIF (*TGIF1/2*)	*TGIF* is essential for normal gastrulation and for proper formation of the AVE.	[59]
LIM	*—*	*LHX (LIM‐HD*, e.g., *LHX2, LHX3, LHX4, LHX6)*	*LHX3* and *LHX4* are involved in the development of pituitary gland and nervous system.	[60]
POU	*—*	*POU1F1, POU2F1 (*also known as *OCT1)*	*POU2F1* is expressed in various cell types and tissues and is involved in the regulation of CD4^+^ T‐cell differentiation.	[60]
HNF	*—*	*HNF1A* and *HNF1B*	*HNF1A* and *HNF1B* are key regulators of embryonic development and adult homeostasis and are involved in an autoregulatory network within the liver, kidney, pancreas, and gut.	[61]
SINE	*—*	*SIX1/2*	*SIX1* regulates progenitor cell expansion, differentiation, and the development of various tissues such as kidneys.	
CUT	*—*	CUX (*CUX1/2*)	*CUX1* is highly expressed in the developing and adult CNS. Its expression has also detected in the human hippocampus and cortex, particularly in pyramidal neurons of layers II–V.	[62]
		ONECUT (*ONECUT1–3*)	*ONECUT1* is involved in early pancreatic development and endocrine differentiation.	[63]
		SATB (*SATB1/2*)	*SATB1* expression is critical for embryonic development, for immune‐system maturation, and supports the pluripotent state of HSCT in the adult bone marrow.	[64]
PROS	*—*	PROX (*PROX1/2*)	*PROX1* is a key developmental regulator required for the morphogenesis of multiple organs (e.g., spinal cord, brain, retina, and liver) and specification of lymphatic endothelial identity.	[65]
ZF (Zinc‐finger homeobox)	*—*	ZHX (*ZHX1–3*)	*ZHX2* has been implicated in erythroid differentiation.	[66]
CERS (Cerberus/other)	*—*	*CERS1–6*	*CerS1* plays a pivotal role in brain development.	[67]

Abbreviations: *ALX1*: *ALX homeobox 1*; *ARGFX*: *Arginine‐fifty homeobox*; *AVE*: *Anterior visceral endoderm*; *BARHL1*: *BarH‐like homeobox 1*; *BARX1*: *BARX homeobox 1*; *CDX1*: *Caudal type homeobox 1*; *CER1*: *Cerberus 1*; *CPHX1*: *Cytoplasmic polyadenylated homeobox 1*; *CUX1*: *C like homeobox 1*; *DLX1*: *Distal‐less homeobox 1*; *DPRX*: *Divergent paired‐related homeobox*; *DUXA*: *Double homeobox A*; *EVX1*: *Even‐skipped homeobox 1*; *GBX1*: *Gastrulation brain homeobox 1*; hESCs: human embryonic stem cells; *HESX1*: *HESX homeobox 1*; *HHEX*: *Hematopoietically expressed homeobox*; *HMX1*: *H6 family homeobox 1*; HSCs: hematopoietic stem cells; *IRX1*: *Iroquois homeobox 1*; IVD: intervertebral discs; *LBX1*: *Ladybird homeobox 1*; *LHX2*: *LIM homeobox 2*; *MEIS1*: *Meis homeobox 1*; *MEOX1*: *Mesenchyme homeobox 1*; *MKX*: *Mohawk homeobox*; MNs: motor neurons; *MNX1*: *Motor neuron and pancreas homeobox 1*; *MSX1*: *Muscle segment homeobox 1*; *NKX2–5*: *NK2 homeobox 5*; *NOBOX*: *NOBOX oogenesis homeobox*; *NOTO*: *Notochord homeobox*; *ONECUT1*: *One cut homeobox 1*; *OTX1*: *Orthodenticle homeobox 1*; *PAX1*: *Paired box 1*; *PAX2*: *Paired box 2*; *PAX3*: *Paired box 3*; *PAX4*: *Paired box 4*; *PBX1*: Pre*‐B‐cell leukemia homeobox 1*; *PDX1*: *Pancreatic and duodenal homeobox 1*; *PHOX2A*: *Paired‐like homeobox 2A*; *PITX1*: *Paired‐like homeodomain transcription factor 1*; *PREP1 (PKNOX1)*: *PBX/Knotted 1 Homeobox 1*; RGC: retinal ganglion cell; *RHOX1*: *Reproductive homeobox on the X chromosome 1*; *SATB1*: *Special AT‐rich sequence‐binding protein 1*; *SHOX*: *Short stature homeobox*; *SIX1*: *Sine Oculis Homeobox 1*; *TGIF1*: *TGFB‐induced factor homeobox 1*; *TPRX1*: *Tetra‐peptide repeat homeobox 1*; *VAX1*: *Ventral anterior homeobox 1*; *VENTX*: *Vent homeobox*.

Among the major homeobox gene classes, the Paired (PRD) class represents the second most extensive group after ANTP in the human genome. Genes in this class are critically involved in regulating developmental processes, particularly in early embryogenesis. This class is generally subdivided into two distinct subclasses: the Paired‐box (PAX) (paired‐type homeobox) subclass, which includes genes directly related to the PAX family, and the PAX‐like (PAXL) subclass, encompassing genes that are structurally or functionally divergent from PAX but still share homology within the paired‐like domain. The PAXL subclass comprises a diverse set of approximately 28 gene families and also consists of pseudogenes. This subclass is also commonly referred to in the literature as PRD‐like homeobox genes. Some notable examples of key gene families within this subclass include *Aristaless‐related homeobox (ALX)*, *Arginine‐fifty homeobox* (*ARGFX*), *Paired‐like homeobox* (*PHOX*) *genes*, *Cytoplasmic polyadenylated homeobox* (*CPHX*), *Orthodenticle homeobox* (*OTX*), *Divergent paired‐related homeobox* (*DPRX*), *short stature homeobox* (*SHOX*), *Paired‐like homeodomain TFs* (*PITX*), *Double homeobox* (*DUX*), and *Tetra‐peptide repeat homeobox* (*TPRX*) [23, 53]. In humans, several members of the PRD‐like subclass are preferentially expressed in the germline and during the earliest stages of development, including in oocytes and zygotes, highlighting their potential role in preimplantation development. For instance, transcripts of genes such as *DPRX*, *NOBOX oogenesis homeobox* (*NOBOX*), and *ARGFX* have been detected [20, 53].

Within the PRD class, genes in the PAX subclass are notable for encoding a distinct group of TFs marked by the inclusion of a highly conserved DNA‐binding region known as the paired domain (PD). This domain, composed of approximately 128 amino acids, is structurally unique among homeobox proteins due to the inclusion of a paired‐box motif a signature feature that has remained remarkably conserved across evolutionary lineages and sets *PAX* proteins apart from other homeobox families. The strong evolutionary conservation in the PD domain underscores its critical function in mediating the DNA‐binding activity of PAX proteins during gene regulation. Owing to this conservation, PAX proteins frequently exhibit similar DNA‐binding motifs; nonetheless, each member of the family achieves functional specificity by regulating a distinct repertoire of target genes. Notably, the expression of PAX genes has been documented across a wide range of organisms, including both vertebrates and invertebrates. Building on their structural features and target specificity, these proteins play essential roles in developmental processes. By directing lineage commitment and promoting cell differentiation, they ensure the coordinated formation of tissues and organs, while also maintaining cell identity to safeguard the long‐term integrity of tissue function. The mammalian PAX family is composed of nine genes (*PAX1* to *PAX9*), which distributed across eight distinct chromosomes, underscoring the evolutionary antiquity of this subclass. These genes are categorized into four structural subgroups, differentiated by the presence of additional conserved domains beyond the PD. Specifically, classification hinges on whether a PAX protein includes a HD, complete or partial, and/or an octapeptide linker. The octapeptide linker contributes to transcriptional repression by interacting with cofactors and other regulatory proteins, thereby attenuating the expression of downstream target genes. Furthermore, each of the nine PAX proteins harbors a C‐terminal transactivation domain. Collectively, this modular organization of these domains has been evolutionarily conserved across higher vertebrates. Group I members, including PAX1 and PAX9, are devoid of the HD and rely solely on the PD for DNA binding, although they also carry an octapeptide linker. Importantly, although the HD domain is dispensable for the core activity of PAX proteins, whether complete or partial it still contributes to the regulation of specific target genes. By cooperating with the PD, the HD further enhances the DNA‐binding capacity of these proteins. Group II proteins, including PAX2, PAX5, and PAX8, possess a partial HD together with the octapeptide. In this configuration, the domain exhibits diminished DNA‐binding capacity. Group III members, including PAX3 and PAX7, contain a complete HD together with the octapeptide, whereas Group IV members, including *PAX4* and PAX6, harbor a full HD but lack the octapeptide. This intact domain incorporates a helix–turn–helix configuration that promotes dimer formation, a property that enhances both the strength and stability of DNA binding [68].

**FIGURE 2:**
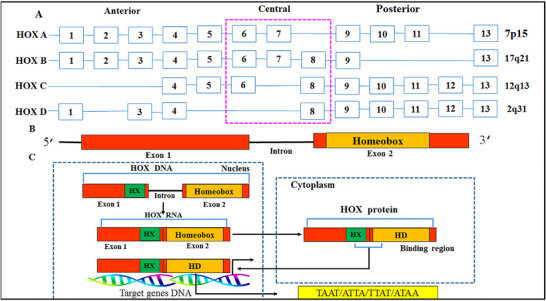
(A) Schematic structure of HOX genes. Structurally, *HOX* genes consist of two exons and a single intron. The second exon encodes a homo domine DNA‐binding domain consisting of 60 amino acids (known as HD). This HD is crucial for the transcriptional regulation of target genes. (B) Transcriptional role of HOX proteins. HOX proteins mediate gene expression by binding to the promoter regions of their target genes, thereby either activating or repressing their transcription. The HD and hexapeptide (HX) motifs present within the HOX proteins are integral for their regulatory activity [74].

While the 11‐class classification provides a comprehensive evolutionary framework, an alternative functional scheme classifies mammalian homeobox genes into two principal categories based on chromosomal organization and structural features. This approach offers additional insights into the regulatory architecture and developmental roles of these genes. The first category, known as Class I, includes the *HOX* genes, which belong to the HOXL subclass of the ANTP class. These genes are organized into four tightly linked clusters HOXA through HOXD located on separate chromosomes (Figure 2 and Table 2) [20, 69, 70]. In total, 39 *HOX* genes contribute to anterior–posterior (AP) axis patterning and are grouped into 13 paralogous, numbered *HOX1* to *HOX13*, based on their sequence homology and positional order within clusters, reflecting evolutionary conservation across the clusters [20, 71]. During vertebrate embryogenesis, HOX gene clusters exhibit the principle of collinearity, whereby their chromosomal arrangement parallels both the spatial domains and the temporal sequence of gene activation. In vertebrates, this synchronization extends across the entire clusters a phenomenon often referred to as whole‐cluster spatio‐temporal collinearity (WSTC) [72, 73]. Specifically, spatial collinearity aligns gene position from 3′ to 5′ with expression domains along the AP axis; temporal collinearity ensures that genes are activated sequentially in developmental time following the same order. A third dimension, quantitative collinearity, has been documented in limb formation, wherein genes located more posteriorly within a cluster tend to be expressed at higher levels. These tightly coordinated mechanisms underscore the evolutionary significance of HOX clustering, serving as a robust framework for establishing precise body plan architecture in vertebrates [20]. The second category, referred to as Class II or non‐HOX/ParaHox genes, is composed of a wide range of gene families (e.g., PAX within the PRD class). Unlike the clustered HOX genes of Class I, members of this group are dispersed across different chromosomal locations, reflecting a higher degree of structural variability [20, 69].

**TABLE 2:** Genomic organization of the four human HOX clusters (*HOXA–HOXD*). Each cluster is located on a distinct chromosome and collectively encodes 39 *HOX* genes. The table summarizes the complete list of genes present in each cluster, their cytogenetic positions, and long noncoding RNAs (*lncRNAs*).

Cluster	Genes	Chromosomal location	Embedded noncoding elements lncRNA	Explanation	References
HOXA	*HOXA1 HOXA2 HOXA3 HOXA4 HOXA5 HOXA6 HOXA7 HOXA9 HOXA10 HOXA11 HOXA13*	7p15‐7p14.2	*HOTTIP* *HOXA‐AS2* *HOXA‐AS3* *HOXA10‐AS* *HOXA11‐AS* *HOTAIRM1*	*HOTTIP* is a polyadenylated lncRNA transcribed from the 5′ end of the *HOXA* cluster. *HOXA‐AS2* is positioned between *HOXA3* and *HOXA4*. *HOXA10‐AS* (also referred as *HOXA‐AS4*) is located at the 3′ end of the *HOXA* cluster *HOXA‐AS2* is positioned between *HOXA1* and *HOXA2*.	[75, 76, 77, 78, 79, 80, 81]
HOXB	*HOXB1 HOXB2 HOXB3 HOXB4* *HOXB5 HOXB6 HOXB7 HOXB8 HOXB9 HOXB13*	17q21.32	*HOXB‐AS1* *HOXB‐AS2* *HOXB‐AS3* *HOXB‐AS4* *HOXB‐AS5 HoxBlinc*	*HOXBLINC* originates from the anterior *HOX*B locus.	[82, 83, 84]
HOXC	*HOXC4 HOXC5 HOXC6 HOXC8 HOXC9 HOXC10 HOXC11 HOXC12 HOXC13*	12q13.13	*HOXC‐AS1* *HOXC‐AS2* *HOXC‐AS3* *HOXC13‐AS* *HOTAIR*	*HOTAIR* is positioned between *HOXC11* and *HOXC12*.	[82, 85]
HOXD	*HOXD1 HOXD3 HOXD4 HOXD8 HOXD9 HOXD10 HOXD11 HOXD12 HOXD13*	2q31.1	*HOXD‐AS1* *HOXD‐AS2*	*HOXD‐AS1* is also referred as *HAGLR*.	[82, 86]

Abbreviations: *HAGLR*: *HOXD Antisense Growth‐Associated Long Noncoding RNA*; *HOTAIR*: *HOX antisense intergenic RNA*; *HOTAIRM1*: *HOX antisense intergenic RNA myeloid 1*; *HOTTIP*: *HOXA transcript at the distal tip*; *HOXA10‐AS*: *HOXA10 cluster antisense RNA*; *HOXA11‐AS*: *HOXA10 cluster antisense RNA*; *HOXA‐AS2*: *HOXA cluster antisense RNA2*; *HOXA‐AS3*: *HOXA cluster antisense RNA3*; *HOXB‐AS1*: *HOXB Cluster Antisense RNA 1*; *HOXB‐AS2*: *HOXB Cluster Antisense RNA 2*; *HOXB‐AS3*: *HOXB Cluster Antisense RNA 3*; *HOXB‐AS4*: *HOXB Cluster Antisense RNA 4*; *HOXB‐AS5*: *HOXB Cluster Antisense RNA5*; *HOXBLINC*: *HOXB locus‐associated lncRNA*; *HOXC‐AS1*: *HOXC cluster antisense RNA1*; *HOXC‐AS13*: *HOXC cluster antisense RNA13*; *HOXC‐AS2*: *HOXC cluster antisense RNA2*; *HOXC‐AS3*: *HOXC cluster antisense RNA3*; *HOXD‐AS1*: *HOXD cluster antisense RNA1*; *HOXD‐AS2*: *HOXD cluster antisense RNA2*.

### 2.2 The Physiological Function of Homeobox Genes

As outlined above, homeobox genes constitute a large family of TFs defined by a conserved homeodomain DNA‐binding motif, and they are widely recognized as master regulators of developmental and cellular processes [74]. Their roles are conserved across a broad range of organisms, from invertebrates to vertebrates, including mammals and humans. The expression of these genes is under precisely regulated in space and time, ensuring that embryonic programs unfold in an orderly manner. This remarkable evolutionary conservation underscores their essential contribution to body plan establishment and organogenesis. Importantly, their role is not confined to embryogenesis. While they orchestrate body‐axis formation and tissue patterning in early development, accumulating evidence shows that certain homeobox genes remain active in postnatal and adult tissues, where they continue to participate in diverse cellular processes such as regulating stem cell (SC) function, maintaining tissue integrity, and preserving long‐term homeostasis. Collectively, these TFs also modulate a broad spectrum of fundamental biological activities, including proliferation, lineage specification, differentiation, hematopoiesis, programmed cell death, migration, angiogenesis, tissue repair, and cell‐cycle regulation, although the relative contribution of individual families to each process can vary considerably [87, 88].

Moreover, individual homeodomain families execute specialized biological roles that reflect their structural and regulatory diversity. This diversity stems not only from variations in DNA‐binding specificity, but also from differences in the presence of associated domains, interactions with cofactors, and the broader transcriptional context in which these proteins function. Such combinatorial mechanisms result in distinct gene regulatory programs across different families [89]. For instance, LHX genes represent a crucial subfamily within the homeobox gene family. These genes encode LIM‐homeodomain (LIM‐HD) proteins, which feature two LIM domains in their N‐termini and a centrally located HD. The HD is responsible for binding specific DNA elements in target genes. Extensive research has demonstrated that these genes encode TFs that regulate gene expression during pivotal developmental processes. The LIM domains enable protein–protein interactions, while the HD directly binds to DNA, thereby influencing the transcription of target genes [90, 91]. Another well‐defined example is the CDX family. The CDX TFs are characterized by the presence of a highly conserved homeobox DNA‐binding domain, which allows them to bind to specific regulatory regions and consequently activate or repress the transcription of their target *HOX* genes. CDX genes’ family encodes a group of TFs that play a critical role in the regulation of *HOX* genes’ expression during embryonic development. The CDX proteins act as crucial upstream regulators, integrating signals from key signaling pathways such as retinoic acid and Wnt to modulate the activity of *HOX* genes promoter. This regulatory function of the CDX factors is essential for the proper patterning of the AP body axis. Through this mechanism, the CDX family members, which in humans include *CDX1*, *CDX2*, and *CDX3*, orchestrate the spatiotemporal expression of the *HOX* genes, ensuring the coordinated development of the body plan along the *AP* axis [92, 93].

During embryogenesis, certain homeobox genes display prominent, stage‐specific expression patterns, where they act as central regulators of key developmental events. For instance, PRD‐like homeobox genes are selectively expressed during the earliest phases of human embryonic development. Within this group, *ARGFX* and *DPRX* have been reported to function as a transcriptional activator and repressor, respectively, and both are directly implicated in the regulation of embryonic genome activation (EGA) during preimplantation development [53, 94]. *LEUTX*, another example of an early‐expressed gene, encodes a DNA‐binding TF. The complete homeodomain isoform of *LEUTX* has also been shown to be fully competent in initiating the expression of numerous genes associated with EGA [53, 95]. Additional examples of functional specialization can be found in other homeobox families. Members of the MSX family, for instance, act as key regulators of epithelial–mesenchymal transition (EMT). EMT is a well‐known biological process associated with embryonic development and morphogenesis, playing a vital role in tissue remodeling during organogenesis and tissue regeneration. Conversely, families such as PAX and DLX are more prominently involved in driving tissue‐specific lineage commitment and differentiation. These genes help define developmental trajectories by modulating transcriptional networks in a context‐dependent manner [96, 97, 98]. DLX TFs play essential functions throughout vertebrate embryonic development and act as central regulators of early skeletal morphogenesis and bone homeostasis, controlling key processes such as chondrogenesis and osteogenesis. During early development, they are particularly important for craniofacial formation, whereas *HOX* genes primarily govern axial and appendicular skeletal patterning. In addition to their developmental roles, DLX genes also participate in adult bone remodeling. In postnatal bone, *DLX* activity remains essential for skeletal integrity through interactions with osteogenic regulators such as *RUNX2* and *OSX/SP7*. As development progresses, members such as *DLX3* and *DLX6* expand their functions beyond embryogenesis. For example, sustained expression of *DLX3* in chondrocytes has been documented, underscoring its critical role in cartilage development [96, 99].

Beyond embryogenesis, certain homeobox genes, particularly those within the HOX clusters, retain transcriptional activity in certain adult cell types, particularly in adult SCs like mesenchymal stromal cells (MSCs) [11]. For instance, within the homeobox superfamily, the HOX clusters have been identified as a pivotal role in hematopoiesis and hematopoietic SC (HSC) differentiation. *HOXA9* is one of the most abundantly expressed TFs in HSCs, where it acts as a key regulator of stemness and differentiation through the regulation of a broad set of target genes (e.g., *CDK6*, *Erg*, *Foxp1*, *Gfi1*, *SOX4*, and *Lmo2*). Notably, many of these genes are also regulated by other cofactors such as meis homeobox 1 (MEIS1) and HOXB4, indicating the existence of a cooperative regulatory network that underpins HSC maintenance and differentiation. Together, HOXA9 and *HOXB4* play a particularly prominent role in sustaining stemness within HSC populations. During normal hematopoiesis, the gradual decline in these genes’ expression serves as a molecular signal that drives SCs out of quiescence and promotes their progression toward differentiation to lymphoid and myeloid. Consistent with this, genes in the *HOXA* cluster including *HOXA5*, *HOXA7*, and *HOXA9* are progressively downregulated during the differentiation of human pluripotent SCs (hPSCs). Experimental evidence indicates that elevated *HOXA9* levels accelerate the hematopoietic differentiation of human embryonic SCs (hESCs), driving hemogenic endothelial precursors toward primitive and CD45^+^ blood cell lineages. In contrast, *HOXA9* expression undergoes a marked decline as HSCs progress to fully differentiated blood cells. Therefore, while downregulation of *HOXA9* accompanies normal differentiation, its sustained overexpression has been directly linked to leukemogenesis, with elevated *HOXA9* expression consistently detected across multiple acute myeloid leukemia (AML) subtypes [100, 101, 102]. In addition, studies indicate that *PBX1* functions both in early human development and in stage‐ and tissue‐specific roles later in life, with alternative splicing generating isoforms such as PBX1a in the adult brain, PBX1b in embryonic tissues, and PBX1d in CD4^+^ T cells. Its sustained expression across selected organs and immune cell subsets highlights a regulatory versatility that extends into adulthood [57], providing a conceptual bridge to other homeobox genes with persistent activity beyond embryogenesis. As a result of this functional diversity, these proteins influence a wide array of downstream targets with critical cellular roles, for example, regulators of the cell cycle and apoptosis (e.g., *p53*) and angiogenic factors (e.g., vascular endothelial growth factor A [VEGFA]) [21]. A broader example of representative biological processes governed by homeobox genes, alongside examples of their gene targets within various biological and cellular function, is provided in Table 3. It should be considered that some homeobox genes exhibit context‐dependent functions, extending their influence beyond a single biological process. Their regulatory effects can span multiple layers of tissue physiology, for instance, simultaneously linking developmental, regenerative, and immune pathways, as exemplified by *HOXB5* and *HOXA3* [103, 104]. In both in vivo and in vitro experiments, *HOXB5* has been shown not only to promote revascularization and perfusion during ischemic injury by stimulating endothelial and vascular responses, but also to induce proinflammatory mediators such as MCP‐1 and IL‐6 [103]. A further example is provided by HOXA3, which has been implicated in regulating both regenerative and immune‐related aspects of wound repair. Both in vivo and in vitro studies have demonstrated that during wound healing, particularly in diabetic ulcers, *HOXA3* expression facilitates skin repair by promoting migration of keratinocyte and enhancing angiogenesis, while simultaneously reducing inflammatory mediators. Maintained *HOXA3* expression decreases leukocyte accumulation in these wounds and directly supports macrophage maturation. Functionally, HOXA3 suppresses M1 macrophage polarization by attenuating proinflammatory signaling, while driving an M2 phenotype through pathways such as Signal Transducer and Activator of Transcription 6 (STAT6) activation during wound healing. In parallel, *HOXA3* expression contributes to wound healing by stimulating keratinocyte migration, maintaining epidermal integrity, and particularly promoting angiogenesis through upregulation of downstream effectors such as matrix metalloproteinase‐14 (*MMP14*), while downregulating inflammatory mediators including C–C motif ligand 2) *CCL2*(and *CXCL12* [104, 105]. Collectively, these functions illustrate how a single homeobox gene can exert multiple, context‐dependent activities, underscoring the multidimensional and pleiotropic nature of homeobox gene activity.

**TABLE 3:** Representative biological processes regulated by homeobox genes. This table provides an overview of major biological and cellular functions modulated by homeobox transcription factors, including embryogenesis, lineage specification, hematopoiesis, migration, angiogenesis, tissue repair, and immune regulation. Examples of homeobox gene families and their downstream targets are included to illustrate how these regulators orchestrate developmental and physiological processes in both embryonic and adult contexts.

Biological/cellular function	Examples of homeobox gene	Homeobox‐target gene associations	Function summary	References
Embryogenesis and organogenesis	*CDX2*, most genes in HOX clusters (e.g., *HOXA5, HOXB5*)	CDX2→*Pou5f1* (*OCT4*; repressed) *HOXA1*→*Hnf1b, FOXD3, Zic1, LHX5, PAX8, Fgfr3* *HOX5 (HOXA5, HOXB5, HOXC5)*→*Wnt2/2b, Bmp4*	CDX2 directs early embryonic lineage commitment by activating TE genes and maintaining *OCT4* repression, thereby ensuring TE–ICM segregation at the stage of the first cell fate decision. *HOXA1*, one of the earliest anterior HOX genes expressed during embryogenesis, regulates multiple genes involved in early development. Specifically, it regulates *Hnf1b*, *FOXD3*, and *Zic1*, which contribute to hindbrain development. In addition, *HOXA1*‐mediated modulation of *Pax8* and *Fgfr3* by *HOXA1* links to otic placode specification and inner ear formation. During embryogenesis, *Hox5* genes regulate *Wnt2/2b* expression in the distal lung mesenchyme, thereby contributing normal lung development and PD patterning.	[57, 106, 107, 108, 109]
Migration	*HOXA3* *HOXC8*	*HOXA3→MMP‐14*, and *uPAR* *HOXC8*→*KDM1A* (activated)	*HOXA3* facilitates migration of keratinocytes and ECs, which in turn contributes to angiogenesis through the upregulation of *MMP‐14* and *uPAR* genes. Correspondingly, *HOXA3* drives the migration of these cells mediated by *uPAR*. *HOXC8* directly binds to the *KDM1A* promoter and upregulates its expression, which in turn suppresses the migration of SCAPs and consequently limits their osteo‐/dentinogenic differentiation.	[110, 111]
Stem cell regulation/maintenance	*MSX2* *HOXA9, HOXA10*	*MSX2*→*SOX2* (repressed), and *NODAL* (activated) *HOXA9*/*HOXA10*→*NT10B, FZD1, FZD5* (activated)	MSX2 contributes to the destabilization of pluripotency in hPSCs by repressing *SOX2* and inducing *NODAL*, thereby regulating mesendoderm lineage commitment. Its activity occurs downstream of BMP signaling with synergistic activation by Wnt/LEF1 signaling. Notably, *SOX2* facilitates *MSX2* degradation, underscoring their mutual antagonism in stem cell fate regulation. *HOXA9* and *HOXA10* enhance HSC self‐renewal by upregulating components of the WNT pathway.	[101, 112]
Differentiation and lineage specification	*DLX3, DLX5, MSX2* *PDX1* *PAX5* *PBX1*	*DLX3*→RUNX2 (activated), *Ocn* (activated), *Osteoactivin* (activated)/ *DLX5*→*RUNX2* (activated)/MSX2→*RUNX2* (repressed) *PDX1*→MAFA, Ins1, and Slc2a2 PAX5→*CD19*, *Blk*, *RAG2*, *CD81* (activated); PAX5→*Notch1*, *perforin* (repression); PAX5↔Blimp1 (cross‐antagonism) PBX1→*EBF1*, *PAX5*, *GATA1*, *FOG1*	DLX3 promotes osteoblast differentiation by activating *RUNX2*, *Ocn*, and *osteoactivin*. DLX3 has documented an early and transient association with *RUNX2* expression. In contrast, DLX5 sustains *RUNX2* promoter activity in mature osteoblasts. In both osteoblasts and nonosseous cells, DLX3 and DLX5 act as positive regulators, driving endogenous *RUNX2* transcription and promoter activation, in contrast to the repressive effects of *MSX2*. Collectively, these homeodomain proteins coordinate activation and repression of *RUNX2* to regulate osteoblast differentiation and bone formation. PDX1 is essential for pancreatic development and β‐cell identity. During postnatal β‐cell maturation, PDX1 acts together with *FOXA2* to activate downstream targets such as *MAFA*, *Ins1*, and *Slc2a2*, thereby supporting functional maturation, insulin secretion, and contributing to the pathogenesis of *MODY*. PAX5 maintains B‐cell identity by activating lineage‐specific transcriptional networks, promoting B‐lineage genes (e.g., *CD19*, *Blk*, and *RAG2*(, while repressing alternative hematopoietic lineage genes (e.g., *Notch1*, *perforin*). Through cross‐antagonism with the plasma‐cell regulator Blimp1, PAX5 reinforces the binary fate decision by repressing *Blimp1* expression. In addition, PAX5 directly binds to the *CD81* promoter, linking its transcriptional activity to B‐cell activation and, indirectly, to cell migration. PBX1 contributes to lineage specification in B‐cell and megakaryocyte lineages by regulating genes (e.g., *EBF1*, *GATA1*), which are essential for the expansion and maturation of these cells.	[30, 113, 114, 115, 116, 117, 118]
Hematopoiesis	HOXA9 HOXB4	HOXA9→*HOXC4*, *RUNX1*, and *MYB* *HOXB4*→*RUNX1*, *Scl/Tal1*, *Gata2*, *Gfi1* (direct target genes); *Lmo2*, *Erg*, *MEIS1*, *PBX1*, *Nov*, *AhR*, Hemgn (indirect target genes)	*HOXA9* is implicated in the regulation of hematopoietic differentiation by upregulating the expression of *HOXC4*, *RUNX1*, and *MYB*, thereby driving myeloid progenitors’ development. *HOXB4* plays a crucial role in driving the maturation of embryonic stem cell‐derived hematopoietic precursors into HSCs with long‐term repopulating capacity. It regulates a broad network of genes, some as direct transcriptional targets and others indirectly. *RUNX1* and *Gata2* are examples of direct *HOXB4* targets essential for HSC development, while factors such as *Lmo2*, and *MEIS1* are modulated indirectly.	[102, 119]
Neurogenesis	*DLX* family (e.g., *DLX 2, DLX5*) *LHX2* *CUX2*	*DLX2/DLX5*→*Wnt5a* (activated) *LHX2→PAX6, CER1* (activated) *CUX2*→*Neurod*, *p27^Kip1^* (activated)	DLX TFs promote the differentiation of GABAergic interneurons in multiple brain regions (e.g., the olfactory bulb and cortex) through activation of *WNT5A*. *WNT5A* has been identified as a downstream target of DLX2 and, more specifically, is directly regulated by DLX5. In vivo and in vitro studies confirm that WNT5A signaling is essential for GABAergic differentiation, thereby linking *DLX* activity to Wnt‐mediated interneuron development. LHX2 contributes to human neural differentiation by directly activating *PAX6* and inducing *CER1* expression. In hESCs, neural differentiation is enhanced by conditional expression of *LHX2*, while its lack of expression markedly disrupts this process. Through this dual action, LHX2 drives neural fate while inhibiting WNT/BMP signaling via CER1, thereby restricting non‐neural lineages. CUX2 plays a key role in spinal cord neurogenesis by regulating neural progenitor cell‐cycle dynamics and neuronal fate determination. It directly activates *Neurod*, and *p27^Kip1^*, thereby driving interneuron differentiation, promoting neuroblast formation, and facilitating cell‐cycle withdrawal during the development of spinal cord.	[120, 121, 122]
Vasculogenesis and angiogenesis—lymphangiogenesis	*PROX1* *HOXA9* *HOXB5*	*PROX1*→FoxC2, *Ang2*, and *HOXD8* *HOXA9*→*EphB4* (activated) *HOXB5* →ANGPT2, VEGFR2	PROX1 is implicated in the lymphatic development by driving BEC‐to‐LEC differentiation and activating maturation programs through regulation of key genes’ expression within this process. Therefore, PROX1 induces *FOXC2*, *ANG2*, and *HOXD8*. *HOXD8*, in turn, also induces *ANG2* and *PROX1* expression, forming a positive‐feedback transcriptional network that maintains PROX1 in LECs. PROX1–HOXD8 networks govern lymphatic vessel maturation and maintenance. *HOXA9* regulates ECs migration and tube formation by directly binding to the EphB4 promoter and inducing its expression, thereby promoting angiogenesis. *HOXB5* modulates angiogenesis at multiple levels: it promotes endothelial sprouting and modulates adhesion‐related genes’ expression. By upregulating *VEGFR2*, *HOXB5* supports endothelial precursor differentiation and augments the angiogenic response. In parallel, *HOXB5* increases *ANGPT2*, which functions as a downstream mediator of *HOXB5*‐driven angiogenesis and enables endothelial activation.	[123, 124, 125]
Tissue integrity and homeostasis	*Mohawk (MKX)* *HOXA5* *PAX5* *PAX6* *CDX2*	*MKX*→*Col1a1*, and *TNXB* *HOXA5*→*PR* (Progesterone receptor; activated) *PAX5*→*CD19, BLNK* (activated); *PAX5*→CD28, Ccr2, and *Ccl3* (repressed) *PAX6→WNK2*	*MKX* is involved in the maintaining ligament and tendon homeostasis by regulating ECM‐related genes (*COL1A1*, *TNXB*). *HOXA5* regulates *PR* expression by directly activating the *PR* promoter, thereby contributing to breast development, growth, differentiation, and homeostasis in breast cells. PAX5 secures B‐cell lineage commitment by activating B‐cell‐specific programs (e.g., *CD19, BLNK*) while repressing ∼110 lineage‐inappropriate target genes. Several of these repressed genes (e.g., *Cd28, Ccr2*) are re‐expressed in plasma cells. This dual regulatory role governs key processes such as transcription, migration, and signaling during B‐cell development. Notably, the process of gene repression by *PAX5* is also fundamental for hematopoietic homeostasis; for example, repression of *Ccl3* as a chemokine gene in B cells prevents abnormal osteoclast differentiation, thereby maintaining balanced blood and bone development. PAX6 participates in regulating CECs differentiation and sustains corneal homeostasis through its downstream target *WNK2*. The PAX6–WNK2 axis contributes to the preservation of corneal homeostasis by controlling the expression of corneal‐related markers (e.g., *KRT12*, *CLU*).	[126, 127, 128, 129]
Tissue regeneration and repair	*LHX2* *IRX1*	LHX2→*SOX9*, *TCF4*, and *Lgr5* *IRX1*→*SOX9*	LHX2 regulates ectodermal morphogenesis and stem‐cell activity. It is expressed in hair‐follicle buds and, postnatally, in epithelial compartments with abundant stem cells within the follicle (e.g., the secondary hair germ), where LHX2^+^ cells also express specific stem‐cell markers. After skin injury, *LHX2^+^* cells expand and promote wound re‐epithelialization. Functionally, the in vivo study detected that LHX2 activates *SOX9* and *TCF4* while repressing *LGR5*, underscoring its dual role in coordinating wound repair with hair‐follicle cycling. IRX1 is expressed in the oral epithelium, localizing to the gingival basal layer and stromal cells. Functionally, IRX1 enhances proliferation by upregulating *SOX9* and promotes cell migration by activating the EGF signaling. Following injury, *IRX1* becomes activated in the basal layer of the gingiva, coordinating *SOX9* expression and EGF‐pathway engagement to accelerate wound healing and re‐epithelialization in the oral epithelium.	[130, 131]
Immune regulation and inflammatory signaling	*PAX5* *CDX2*	*CDX2*→*TRIM31* *PAX5*→*NEDD9* (activated); *PAX5*→*PTEN* (repressed)	CDX2 is implicated in intestinal inflammation by regulating *TRIM31* expression, with TRIM31 acting as an inhibitor of NLRP3 inflammasome activation through proteasomal degradation via the ubiquitin pathway, thereby limiting *NLRP3* inflammasome assembly. Downregulation of *CDX2* and *TRIM31* results in elevated proinflammatory cytokines (e.g., *IL‐1β, IL‐6*), thereby promoting intestinal inflammation. PAX5 is essential throughout B‐cell development. During early B‐cell commitment, it regulates TFs, receptors, and signaling genes by binding to promoters and enhancers, including activation of the *Nedd9* gene, which mediates B‐cell migration. In mature B cells, PAX5 supports survival and differentiation by suppressing *PTEN*, a negative regulator of PI3K signaling. This repression sustains PI3K pathway activity, which is crucial for the differentiation of mature B‐cell subsets (e.g., MZ B cells).	[132]

Abbreviations: *ANGPT2*: *Angiopoietin‐2*; BECs: blood vascular endothelial cells; *Blimp‐1*: *B‐lymphocyte‐induced maturation protein 1*; *BLNK*: *B‐cell linker protein*; *BMP*: *Bone Morphogenic Protein*; CEC: corneal epithelial cell; *CER1*: *Cerberus 1 gene*; *EBF1*: *Early B‐cell Factor 1*; ECM: extracellular matrix; EGF: epidermal growth factor; *FOG1*: *Forkhead box G1*; *Foxc2*: *Forkhead box protein c2*; *GATA1*: *GATA‐binding transcription factor 1*; ICM: inner cell mass; *INS1*: *Insulin I*; LECs: lymphatic endothelial cells; *MAFA*: *MAF bZIP transcription factor A*; *MMP‐14*: *Matrix metalloproteinase‐14*; *MODY*: *Maturity diabetes of the young*; MZ: marginal zone; *NLRP3*: *NLR family, pyrin domain containing 3*; *NRP1*: *Neuropilin 1*; *Ocn*: *Osteocalcin*; PD: proximal–distal; PI3K: phosphoinositide 3‐kinase; *RUNX1*: *Runt‐related transcription factor 1*; *RUNX2*: *Runt‐related transcription factor 2*; SCAPs: stem cells of the apical papilla; TE: trophectoderm; *TNXB*: *Tenascin XB*; *uPAR*: urokinase‐type plasminogen activator receptor; *WNK2*: *WNK lysine deficient protein kinase 2*.

Among the biological functions of homeobox TFs, notable implications have also been identified in cancer, primarily through the regulation of core processes such as apoptosis, proliferation, cell migration, and angiogenesis. By governing these processes, they assume a central role in cancer biology. Importantly, in a context‐dependent manner, these factors can exert dual effects in cancer acting as oncogenic drivers by promoting tumor growth, invasion, and angiogenesis, or functioning as tumor suppressors by various processes such as apoptosis [3, 133]. For instance, the *empty spiracles homeobox 1* (*EMX1*) and *EMX2* genes exhibit tumor‐suppressor activity by inhibiting the expression of key stemness‐related genes such as *SRY‐*box TF 2 (*SOX2*) and *MYC*, thereby reducing cancer SC (CSC) populations in sarcomas. In vivo studies have further demonstrated that loss of *EMX* expression is directly associated with the enhancement of tumor aggressiveness in these cancers [134]. Together, this dual context‐specific functionality contributes to a highly dynamic and intricate regulatory network. Furthermore, as mentioned, the interaction of homeodomain proteins is not restricted to embryonic stages but also occurs in later developmental and adult contexts. This underscores a deeper fundamental interface of organogenesis and tumorigenesis, mediated by homeodomains that act during embryonic development as well as those involved in cellular differentiation. Such regulatory plasticity exemplifies how homeobox TFs can function differently depending on the biological context [3, 21].

One of the most striking examples of context‐dependent functions among homeobox TFs is angiogenesis, a process in which these genes play pivotal roles across physiological and pathological contexts. During development and tissue repair, homeobox genes influence endothelial differentiation, sprouting, and vascular remodeling, contributing to placental vascularization and wound healing. Dysregulation of homeobox expression is also implicated in cancer, where aberrant angiogenesis facilitates tumor growth, metastasis, and invasion, ultimately impacting prognosis. Consequently, neovascularization represents a core biological process governed by homeobox genes Several studies have documented a close relationship between neovascularization and homeobox proteins, which modulate this process across both embryonic development and pathological conditions through dual mechanisms [133, 135, 136]. In this regulatory landscape, proangiogenic actions are achieved through upregulation of factors such as *VEGFA*, *FGFs*, *TGFs*, and angiopoietins, driving endothelial activation and sprouting. Conversely, antiangiogenic effects emerge via suppression of proangiogenic signals and stabilization of endothelial quiescence, exemplified by restraining components like *VEGFR2* and by promoting antiangiogenic gene programs. Specific *HOX* members exhibit distinct roles: *HOXB5*, *HOXA3*, and *HOXD3* contribute to angiogenic onset and endothelial lineage commitment, whereas *HOXA5* and *HOXD10* can reinforce vascular stabilization and quiescence. *IRX3* has also been identified as proangiogenic, enhancing endothelial migration and influencing tip‐cell fate through *VEGF–*Notch integration. In cancer, the balance tilts toward proangiogenic outcomes when these genes are overexpressed, supporting tumor vascularization and progression. Notably, *HOXB5*, *HOXB7*, *HOXB9*, *HOXC10*, and *DLX4* have been linked to upregulated angiogenic signaling in diverse cancers, with mechanistic examples including *ANGPT2* induction and activation of extracellular signal‐regulated kinase (*ERK*)*/AKT* pathways, as well as STAT1‐mediated iNOS induction [133, 137, 138]. Angiogenesis includes a cascade of events in which endothelial cells (ECs) play a central role. During vascularization, ECs secrete various components and proteins, such as growth factors. Importantly, homeobox genes exert diverse effects on ECs, particularly during differentiation and maturation [133, 139, 140]. For example, *HOXB5* has been shown to regulate angioblast differentiation into mature ECs and to upregulate *ANGPT2*, a key angiopoietin required for ECs sprouting [125, 140]. In addition, *HOXA3* together with *HOXD3* display proangiogenic functions and are expressed at early stages, correlating with endothelial invasion and sprouting, and have been reported to contribute to endometrial cancer (EC) lineage commitment [125, 140]. *IRX3* has also been identified as a proangiogenic factor, directly promoting EC migration and influencing tip‐cell fate through integration of VEGF–Notch signaling [141]. By contrast, *HOXA5* exerts an antiangiogenic effect by suppressing cell migration, upregulating antiangiogenic genes while downregulating proangiogenic ones (e.g., VEGFR2), thereby repressing the angiogenic process. Along with *HOXD10*, it is also associated with endothelial quiescence, contributing to the stabilization of the mature EC phenotype [133, 142, 143]. The relationship between homeobox gene expression and angiogenesis in disease, especially cancer, is highly complex and multifactorial. In various types of cancer, dysregulation of specific homeobox genes frequently contributes to aberrant angiogenesis, in part through their regulation of key proangiogenic related factors such as *VEGF* and *FGFs*. Therefore, overexpression of homeobox genes with proangiogenic properties leads to enhancement of tumor angiogenesis, thereby supporting tumorigenesis and its progression [133, 137]. Genes such as *HOXB5*, *HOXB7*, *HOXB9*, *HOXC10*, and *DLX4* have been reported to be upregulated in different cancers, promoting vascular expansion and tumor growth [137, 144, 145, 146]. For instance, *HOXB5* overexpression in esophageal cancer leads to enhance the level of *ANGPT2* expression, which in turn activates the extracellular signal‐regulated kinase (*ERK*/*AKT)* pathway, thereby enhancing angiogenesis and promoting both proliferation and metastasis [144]. Similarly, *DLX4* overexpression has been detected in ovarian cancer, where it promotes angiogenesis through a STAT1‐dependent mechanism that induces iNOS expression. Increased the level of iNOS is strongly linked to augmentation of tumor angiogenesis [144]. The subsequent sections will elaborate in greater detail on how alterations in the expression and function of homeobox genes contribute to cancer development and progression.

#### 2.2.1 The Function of Genes Within HOX Clusters

Among homeobox gene families, *HOX* genes hold particular significance, not only because of their indispensable developmental roles but also due to their clustered genomic organization and the extensive body of research focused on them [147, 148]. As pivotal transcriptional regulators, *HOX* genes play a vital role in the regulation of patterning and cell fate throughout embryogenesis [3]. (Some examples are summarized in Table 3.) From a biological perspective, the function of the HOX proteins encompass numerous aspects of embryonic development, cellular physiology, and tissue homeostasis [149]. Particularly during embryonic development, the expression of *HOX* genes, along with intricate gene networks, determines the temporal and spatial development of human limbs [82, 150]. Within this developmental framework, HOX TFs are indispensable for the AP axis patterning across bilaterian animals through regulation of downstream target genes. Beyond the axis patterning, HOX proteins also participate in regulating diverse organogenesis, specification of individual cell types, and the coordination of morphogenetic programs throughout development. Notably, by modulating the expression of their downstream targets, HOX TFs ensure the establishment and diversification of morphological patterns along the AP axis. Such tightly regulated activity has been observed across diverse embryonic and adult tissues, highlighting the central role of HOX TFs in coordinating regional identity, developmental processes, and cell‐type specification throughout the body [11, 151, 152, 153].

As mentioned earlier, the activities of *HOX* genes are notably not limited to embryonic development but continue to influence key cellular functions in adult tissues throughout the human lifespan [11, 152, 153]. As mentioned earlier, these TFs contribute to the maintenance and differentiation of both embryonic and adult SCs, helping direct lineage‐specific outcomes in processes such as adipogenesis and neurogenesis. SCs are a type of cells that possess an extraordinary ability to both renew themselves and differentiate into various cell types across multiple lineages and also generate diverse cell types. These genes further support the differentiation of tissue‐specific SCs into various specialized cell types required for specific lineages within the adult body. Among their known roles, *HOX* genes are crucial in directing SCs differentiation across various biological pathways, such as the development of adipose tissue and neurogenesis [11, 154, 155]. A unique feature of *HOX* genes is their ability to reprogram the entire body regions’ identity, a process known as homeosis [11, 152, 153]. Furthermore, HOX proteins also participate in nontranscriptional activities, influencing the regulation of critical cellular processes such as DNA replication and repair, mRNA translation, and protein degradation [149]. Importantly, *HOX* genes extend their influence beyond developmental programs to regulate key processes in adult tissues. In cancer, their expression is often dysregulated, with specific genes being either upregulated or downregulated depending on the biological context. Such alterations have been documented across a wide spectrum of malignancies, thereby underscoring the importance of these TFs in both physiological development and pathological conditions [3, 133].

Structurally, these genes are typically divided into two exons and one intron, with the homeobox sequence located in the second exon. The functions of the HOX are reliant on a conserved 60‐amino acid HD and a hexapeptide motif (HX) that have been evolutionarily preserved. The HD is predominantly involved in binding to DNA at specific recognition sites, ultimately resulting in the transcriptional regulation of target genes through either activation or inhibition [74]. The HD acts as a DNA‐binding domain, with a preference for recognizing a specific TA‐rich core DNA sequence, such as TAAT or TTAT, among other functions [149]. Consequently, *HOX* genes perform key TF functions that have been preserved throughout evolution and are found in all bilaterian animals [149]. The unique structural arrangement of HOX proteins, consisting of three helices, is crucial for their DNA‐binding function. The helix–turn–helix DNA‐binding motif allows them to recognize –TAAT– motifs and facilitate binding. Both helices 2 and 3 adopt the helix–turn–helix configuration, which is a defining feature of TF binding. The HD primarily attaches to DNA by engaging helix 3, also known as the recognition helix, within the major groove of the DNA. This fundamental role of HD proteins lies in their ability to modulate the expression of various genes [156]. Interestingly, HOX proteins demonstrate nearly identical affinity in binding to these sites, underscoring the HD as a defining aspect of HOX transcriptional regulation [7, 151, 157, 158]. In addition to the HD, HOX proteins also contain an acidic C‐terminal tail (C‐tail) that allows them to bind with the three‐amino acid loop extension (TALE) and serve as a cofactor. Furthermore, a HX motif has been detected in some HOX proteins, which includes a strongly preserved YPWM motif and a flexible linker region. This motif is essential in determining TALE cofactor selectivity, which in turn regulates specific *HOX* target genes. The binding of HOX cofactors improves the stability of HOX‐DNA interactions [82, 150, 157].

#### 2.2.2 The Involvement of Homeobox Genes in Various Signaling Pathways Under Physiological Condition

Across the human lifespan, a conserved set of signaling pathways, particularly Wnt/β‐catenin, Notch, Hedgehog (Hh), TGF‐β, bone morphogenetic protein (BMP), mitogen‐activated protein kinase (MAPK)/ERK, PI3K/AKT, Janus tyrosine kinase (JAK)/STAT, and NF‐κB, are frequently implicated from embryogenesis through adulthood to execute context‐specific biological processes such as tissue homeostasis, repair, and immune response regulation. Therefore, any dysregulation of these signaling pathways is intimately associated with various pathological conditions, particularly cancer [11, 159, 160, 161, 162]. Within this integrated network, homeobox TFs participate by interfacing with major pathways (e.g., WNT, Notch, and NF‐κB) to coordinate diverse biological processes such as development, cell differentiation, and immune responses. For instance, HOX clusters have been implicated in the regulation of pathways such as WNT and MAPK [11, 87]. These interactions influence both physiological stages, from embryogenesis to adulthood, as well as various pathological conditions, most notably cancer. Therefore, homeobox TFs typically serve dual roles, as downstream targets and as upstream regulators of these pathways, depending on cellular and developmental context. For example, MSX1 and MSX2 serve as downstream mediators of BMP2 signaling during woman endometrial decidualization. Moreover, during cardiogenesis, NKX2‐5 enhances canonical WNT signaling by upregulating *R‐spondin3*, thereby promoting this pathway activation [11, 87, 163, 164]. Other representative examples of these pathways under physiological conditions, along with selected homeobox genes implicated in each pathway, are summarized in Table 4.

**TABLE 4:** Representative examples of the involvement of homeobox genes in some important signaling pathways.

Pathway	Core physiological function	Representative homeobox–pathway interactions and associated mechanisms	References
Wnt signaling	Cell fate determination, cell proliferation, tissue homeostasis, migration, survival	*PAX3* → Its TF binds the *WNT1* promoter and regulates its expression during embryonic development. *DLX2* → Its TF transcriptionally activates *WNT1*, leading to upregulation of Wnt/β‐catenin signaling and promotion of osteogenic differentiation. *PROX1* → enhances Wnt/β‐catenin signaling by forming a complex with β‐catenin/TCF7L1, driving the expression of *FOXC2* and *GATA2* in lymphatic endothelial cells.	[165, 166, 167, 168]
*NF‐κB* signaling	Embryonic development, hematopoiesis, innate immunity, inflammatory responses	*HOXC4* → its expression enhances the proliferation of hematopoietic progenitors (e.g., myeloid and erythroid lineages), leading to increased proportion of *CD43^+^* cell. This hematopoietic effect is linked to the upregulation of NF‐κB signaling and is likely mediated by alterations in cell‐cycle dynamics. *HOXA9* → contributes to hematopoiesis and hematopoietic differentiation; its overexpression enhances hematopoiesis and increases CD34^+^ cell numbers while decreasing erythroid progenitors, effects closely associated with NF‐κB pathway activation.	[119, 169, 170]
Notch signaling	Cell fate determination, expansion, survival, self‐renewal, and differentiation during development, along with hematopoiesis, and T cell differentiation	*HOXB4* → contributes to the regulation of HPCs proliferation during early thymic colonization; Notch signaling together with TNF‐α upregulates *HOXB4* and *GATA3* transcription, thereby modulating T cell differentiation, proliferation, and self‐renewal. *DLX1*/*DLX2* → their expression contribute to neurogenesis in the subcortical telencephalon, acting in concert with Mash1 as demonstrated in in vivo studies. DLX1/DLX2 modulate this process through regulation of Notch signaling: Mash1 promotes early neurogenesis, whereas DLX1 and DLX2 are essential to downregulation of Notch activity in late progenitors, thereby regulating cell differentiation and specification.	[171, 172]
EGFR signaling	Cell growth, proliferation, differentiation	*HOXA7* → promotes granulosa cell proliferation, differentiation, and cell growth by upregulating *EGFR* expression, thereby enhancing EGFR‐mediated signaling activity. *DLX2* → regulates postnatal neurogenesis in the subventricular zone by promoting NSC‐to‐TAP lineage progression and enhancing neuronal progenitor proliferation via EGFR signaling.	[173, 174]
BMP signaling	Limb patterning, morphogenesis, axial growth, bone development, skeletal maintenance	*DLX5/6* and *MSX1/2* → coordinate limb development and morphogenesis through two complementary mechanisms: a cell‐autonomous mechanism in which DLX TFs modulate *MSX2* expression within AER and limb mesoderm cells, and a noncell‐autonomous mechanism where BMP2/4 act as mediators of a regulatory loop linking the AER and the anterior limb mesoderm. *DLX5* → contributes to BMP2‐induced osteogenesis of MSCs by upregulating osteogenic factors such as *RUNX2*; it acts as a central mediator of the BMP signaling pathway and drives osteogenic differentiation toward the osteogenic lineage.	[175, 176]
RA signaling	Embryonic patterning, organogenesis, limb development, cellular differentiation	*SHOX2* → upregulated by RA signaling in cooperation with WNT signaling during pacemaker differentiation; this synergy promotes hiPSC differentiation into sinus node‐like cells and, when overexpressed, *SHOX2* further induces pace‐like cells with enhanced physiological pacing, reflecting the diverse regulatory role of RA in cardiac development. *HOXA1* → contributes to ESCs differentiation and is induced by RA signaling through the involvement of a distal enhancer; this enhancer engages in long‐range chromatin looping to sustain RA‐mediated *HOXA1* expression and regulate downstream target genes during early ESC differentiation in an in vitro study.	[177, 178, 179]
*MAPK* signaling	Embryonic development, differentiation, proliferation	*MSX1 *→ contributes to cell proliferation during limb development; CDK1‐dependent phosphorylation at the Ser136 residue of MSX1 enhances the upregulation of *FGF9/18* expression, which in turn facilitates proliferative signaling through activation of the MAPK pathway, underscoring the role of the MSX–FGF–MAPK axis in limb morphogenesis.	[180]

Abbreviations: AER: apical ectodermal ridge; *CDK1*: *Cyclin‐dependent kinase 1*; EGFR: epidermal growth factor receptor; ESCs: embryonic stem cells; FGF: fibroblast growth factor; Fgf: fibroblast growth factor; *FOXC2*: *Forkhead box protein C2*; hiPSC: human‐induced pluripotent stem cells; HPCs: hematopoietic progenitor cells; *MAPK*: *Mitogen‐activated protein kinase*; NSCs: neural stem cells; *RA*: *retinoic acid*; TAPs: transit‐amplifying precursor.

Cells regulate diverse biological processes through intricate networks of receptors and signaling pathways that integrate multiple inputs and engage in extensive crosstalk, enabling context‐specific responses [181]. For instance, the WNT/β‐catenin pathway shows crosstalk with RA, BMP, Notch, NF‐κB, and Hh pathways. During osteogenesis, WNT/β‐catenin signaling potentiates *BMP*‐dependent target gene expression, promoting osteogenic differentiation [165, 182, 183]. In cardiac development, RA and WNT signaling play fundamental roles in morphogenesis and heart development. NF‐κB interacts with WNT, MAPK, TGF‐β, PI3K/AKT, and JAK/STAT pathways to regulate inflammation and immune responses [161]. Homeobox TFs likewise act as crucial regulators that integrate and mediate crosstalk among signaling networks. For instance, in vitro studies with murine F9 cells show that *RA*‐induced collinear activation of HOX cluster genes (A–D) requires PI3K/Akt signaling during gastrulation, suggesting that crosstalk helps gastrulating cells preserve positional information critical for AP axis formation [184]. In vivo and ex vivo work indicate that NKX2–5 contributes to the regulation of hemogenic and cushion endocardial cell generation through Notch activation, while RA activity is concurrently repressed by dehydrogenase/reductase 3 (*Dhrs3*); together, the NKX2‐5/Notch/RA signaling axis promotes the differentiation of these cells into macrophages implicated in the remodeling of cardiac valve. On the other hand, disruption of signaling crosstalk common in cancer can rewire pathway interconnections to promote tumor progression. In lung adenocarcinoma (LUAD), for example, upregulated WNT/TCF4–HOXB9 signaling facilitates metastasis to bone, augmenting aggressiveness [185]. More broadly, although WNT signaling maintains normal tissue homeostasis, its dysregulation drives oncogenesis; collectively, these findings underscore how alterations in Homeobox‐related signaling pathways can redirect developmental programs to sustain cancer [137, 165, 181].

## 3 Functional Involvement of Homeobox Genes in Human Diseases

Homeobox genes encode pivotal developmental TFs with precise, context‐dependent functions that influence diverse physiological processes throughout life, including maintenance of adult tissue homeostasis. Because these factors regulate broad gene networks and intersect with major signaling pathways (e.g., WNT/β‐catenin, Notch, BMP/TGF‐β), perturbations in signaling cascades can reprogram homeobox regulatory circuits and downstream outputs. As a result, alterations in homeobox activity or expression can propagate through gene‐regulatory networks, contributing to developmental anomalies or various pathological states [3, 11, 186]. Dysregulation of homeobox genes arises through multiple mechanisms, including somatic mutations, signaling perturbations, epigenetic modifications (notably DNA methylation), and noncoding RNA (ncRNA)‐mediated regulation [3, 187]. Clinically, dysregulation of homeobox genes is observed across a broad range of diseases from developmental and congenital disorders and neurodevelopmental and neurodegenerative conditions to diverse cancers [3, 187]. In cancer, homeobox genes often serve as context‐dependent modulators, acting as oncogenes or tumor suppressors depending on tissue context, and their dysregulation can drive tumor initiation, progression, and metastasis [138, 188, 189]. Emerging evidence highlights widespread DNA methylation changes affecting homeobox gene expression as a key driver of oncogenic programs [190, 191].

Furthermore, mutations within homeobox genes themselves have been identified across diverse cancer types in both germline and somatic contexts. A notable case study is *HOXB13*, which illustrates germline‐somatic contributions to oncogenesis, particularly in prostate cancer (PCa) [187, 192, 193]. In the germline context, a rare *HOXB13* missense variant (*p.Gly84Glu*) has emerged as an important hereditary risk factor for PCa. Beyond PCa, evidence suggests this variant may be associated with increased risk for other cancers, including nonmelanoma skin cancer and rectosigmoid cancer, observed exclusively in male carriers. Notably, this same variant was previously reported to confer an elevated risk of colorectal cancer (CRC), particularly in a small number of mutation carriers with a family history of PCa [193, 194, 195]. In the somatic context, upregulation of *HOXB13* has been observed in primary prostate tumors and is correlated with more aggressive disease, advanced tumor grade and an increased propensity for metastasis, particularly following prostatectomy. This same variant has also been reported to confer elevated CRC risk, particularly among mutation carriers with a family history of PCa. Together, these findings underscore how germline *HOXB13* alterations can influence cancer susceptibility across tissues and highlight the need for integrative studies of *HOX* gene mutations in hereditary cancer predisposition [192]. While mutation insights are significant, this review primarily focuses on the epigenetic regulation of homeobox genes in cancer, with particular emphasis on DNA methylation. Beyond genetic alterations, epigenetic mechanisms have emerged as pivotal regulators of gene expression across a broad spectrum of human disorders, spanning cancer and noncancer conditions, with DNA methylation often serving as a principal modulator of gene activity [196]. In the sections that follow, representative noncancerous conditions and neurodevelopmental disorders linked to alterations in homeobox gene expression are outlined, supported by genetic and epigenetic evidence. Later sections address cancer biology involving homeobox dysregulation, with particular attention to the relationship between aberrant DNA methylation and malignancy, and to lung cancer, where epigenetic silencing or activation of homeobox genes is strongly implicated in tumor pathogenesis.

### 3.1 Noncancerous Diseases Associated With Homeobox Genes

Although homeobox genes are widely recognized for their roles in oncogenesis, mutations or dysregulation of these genes are equally central to the pathogenesis of numerous noncancerous human diseases [3, 7, 187]. Because homeobox TFs coordinate tightly regulated developmental programs, perturbations in their expression or activity can yield highly context‐specific malformations and functional impairments. Consequently, disruptions of homeobox function, or dysregulation of their expression arising from pathogenic variants or epigenetic modifications, can have far‐reaching consequences across congenital and organ‐specific disorders, including congenital malformations, metabolic syndromes, cardiac anomalies, and neurodegenerative conditions (addressed in the next section) [187, 197, 198, 199]. For instance, dysregulation of *HOX* genes at various stages of embryonic and postnatal development has been linked to skeletal malformations such as hand–foot–genital syndrome, syndactyly, and other limb malformations [3, 11, 186].

#### 3.1.1 Congenital and Organ‐Specific Disorders Associated With Homeobox Genes

Because homeobox TFs regulate a broad array of developmental processes, especially morphogenesis and cell differentiation, there is strong evidence for their direct involvement in human disease. Pathogenic variants identified in affected patients often supported by animal models with corresponding gene disruptions, support the conclusion that mutations in these genes can cause serious developmental disturbances. Phenotypes frequently involve congenital defects or organ‐specific abnormalities arising from disrupted patterning and differentiation [200, 201]. For example, *PBX1* deletions lead to haploinsufficiency (HI) and syndromic congenital anomalies of the kidney and urinary tract (CAKUT), with renal defects also observed in *Pbx1*‐null mice. Given the complexity of homeobox gene biology in noncancerous conditions, this section highlights the most frequently reported variant types in human disorders [16]. Table 5 summarizes representative examples of variants across different disease categories and outlines disorders associated with various classes of coding mutations, such as missense, nonsense, and frameshift. It should be noted that, for each gene, a wide range of mutations may be reported across different patients; the examples in Table 5 illustrate representative variant types and mechanisms rather than the full mutational repertoire. As a prominent example, around 700 distinct variants have been reported in *PAX6*, associated with a broad spectrum of ocular abnormalities, including aniridia, cataract, and foveal hypoplasia [202, 203]. Notably, nonsense mutations introduce premature termination codons (PTCs) and typically cause loss‐of‐function (LOF) through truncated proteins. PTCS can also arise from frameshift, splice‐site, or single‐nucleotide variants. The downstream consequences of PTC‐generating variants depend on stop‐codon position and the domain context: most truncations are functionally inactive, but in certain settings they can exert dominant‐negative effects or confer gain‐of‐function (GOF) [204, 205]. For instance, among noncancerous HOX‐related disorders, a nonsense variant in *HOXA2* yields a truncated protein consistent with LOF and autosomal‐dominant bilateral microtia, while missense mutations within the CRX homeodomain can drive dominant retinopathies via GOF mechanisms [206, 207]. Quantitatively, across inherited human diseases, about one‐third of pathogenic variants are nonsense, underscoring their substantial contribution to the pathogenic‐variant spectrum; prior studies similarly estimate that nonsense and frameshift variants introducing PTCs account for roughly one‐third of characterized human genetic disorders [204, 205].

**TABLE 5:** Summary of homeobox gene variants implicated in noncancerous disorders.

Disease category	Disease/syndrome	Implicated homeobox gene(s)	Mutation type/variant	Molecular effect/mechanism	References
Congenital malformations → neurological and neurodevelopmental disorders	Hypomyelinating leukodystrophy (severe form)	*NKX6‐2*	Frameshift (c.606delinsTA, p.Lys202Asnfs*?), nonsense (c.565G>T, p.Glu189*), missense (c.599G>A, p.Arg200Gln)	Biallelic inactivating variants in *NKX6‐2* lead to LOF and disrupt its transcriptional activity. Frameshift and nonsense variants occur within the homeodomain, where nonsense mutations introduce PTC. Missense variants also affect highly conserved residues, impairing DNA binding. These alterations are associated with a severe neurodevelopmental phenotype characterized by hypomyelinating leukodystrophy.	[208]
	HCFP3	*HOXB1*	Missense mutation (c.763C>G, p.Arg255Gly; c.781C>T, p.Arg261Cys)	The identified variants are predicted to reduce the functional capacity of HOXB1. Importantly, this report describes a biallelic combination of pathogenic variants in HCFP3 for the first time.	[209]
	CCHS	*PHOX2B*	Nonsense mutation (c.83C>G, p.Ser28*)	The nonsense mutation occurs in exon 1 and has been identified in patients with CCHS with phenotypic variability.	[210]
Limb and skeletal malformations	MDUGA	*HOXA11*	De novo heterozygous variant (c.881T>G, p.Met294Arg)	This variant occurs within the HD, disrupting both DNA binding and protein–protein interactions, thereby impairing HOXA11 activity. These molecular defects are consistent with the forelimb and hindlimb malformations and urogenital abnormalities observed in both in vivo models and patient studies of MDUGA.	[211]
	HFGS	*HOXA13*	Missense mutation (c.1123G>T, p.V375F)	The mutation of *HOXA13* disrupts DNA‐binding and transcriptional activation of target genes (e.g., *EPHA7*), contributing to the malformations observed in HFGS.	[212]
	SPD	*HOXD13*	Missense mutation (c.G917T, p.R306L)	The mutation in *HOXD13* does not cause HI. Its homozygous mutation enhances Smad5 phosphorylation and thereby disrupts the HOXD13‐pSmad5 interaction, activating the Smad5/p65/c‐Fos/Rank signaling axis and leading to increased osteoclast differentiation.	[213]
	LWD/LMD	*SHOX*	Missense mutations (c.508G>C, p.A170P)/(c.509C>A, p.A170D)	Missense mutations (p.A170P, p.A170D) disrupt nuclear translocation and impair the function of the SHOX protein. Particularly, the A170P mutation induces aberrant subcellular distribution of the protein. Despite being expressed throughout the growth plate, the mutant protein is associated with irregular alignment of chondrocytes.	[214]
	Tibial hemimelia and mirror‐image polydactyly (lower‐limb malformations)	*PITX1*	35 bp deletion in exon 3 (c.765_799del) causing a frameshift mutation (p.Ala256ArgfsX303)	The 35 bp deletion introduces a frameshift and a PTC, leading to haploinsufficiency and loss of the C‐terminal OAR domain, which is essential for DNA binding. This molecular defect impairs PITX1 function, thereby contributing to a spectrum of lower‐limb malformations.	[215]
	SHFM1	*DLX5*	Missense mutation (c.558G>T, p.Gln186His)	The mutation in *DLX5* is located within its DNA‐binding domain, impairing both DNA‐binding affinity and transcriptional activity, thereby leading to SHFM1.	[216]
Congenital malformations → craniofacial and dental anomalies	Craniosynostosis (syndromic/multsuture)	*PRRX1*	Multiple heterozygous variants such as a missense mutation (c.161A>C, p.Asp54Ala), a nonsense mutation (c.283C>T; p.Arg95∗), and a single‐nucleotide deletion (c.52del) expected to cause a frameshift (p.Arg18Alafs∗23)	Multiple *PRRX1* variants have been associated with craniosynostosis. For example, missense mutations can impair PRRX1 nuclear localization; in particular, some variants cause partial or complete loss of function, leading to haploinsufficiency.	[217]
	Congenital tooth agenesis	*MSX1*	Heterozygous deletion (c.433_449del) causing frameshift (p.Trp145Leufs*24)	The frameshift mutation results in a PTC and the production of a truncated protein, ultimately causing impairment of MSX1 function and leading to tooth agenesis.	[218]
	Craniosynostosis (Boston‐type)	*MSX2*	Missense mutation (c.443C>T, p.Pro148Leu)	Missense mutation probably changes the DNA‐binding activity of MSX2.	[219]
	TA	*PAX9*	>150 variants; >50 mutation types → missense, deletion, nonsense, insertion, frameshift mutations	*PAX9* mutations are most frequently associated with molar defects in TA, with the type and position of mutations being associated with variable degrees of PAX9 functional impairment and disease severity. Null mutations cause severe TA due to alterations in PAX9 functions (e.g., transactivation and DNA binding) and are associated with HI, whereas in‐frame mutations exert milder effects.	[220]
	TDO syndrome	*DLX3*	Frameshift mutation (c.604_605del، p.S202*)	The mutation impairs DLX3 transcriptional activity and downregulates *EMP* genes, leading to decreased enamel thickness and hardness.	[221]
	Frontonasal dysplasia spectrum	*ALX4*	Nonsense mutation (c.793C>T, p.R265X)	Mutations in the *ALX4* gene lead to a PTC, generating truncated, nonfunctional proteins. These mutations are associated with defective differentiation of the interfollicular epidermis and result in a severe form within the frontonasal dysplasia spectrum.	[222]
Ocular malformations	Aniridia	*PAX6*	Nonsense (c.718C>T, p.Arg240*; c.299G>A, p.Trp100*), frameshift (c.112del, p.Arg38Glyfs*16) mutations	Mutations lead to the production of truncated PAX6 proteins with impaired DNA‐binding, mostly due to PTC variants that result in haploinsufficiency. These mutations are also associated with anterior lens capsule rupture in aniridia.	[223]
	Severe myopia	*OTX2*	Missense mutation (c.235G>A, p.Glu79Lys)	The mutation is located within the protein's DNA‐binding domain, causing protein destabilization and impairment of its activity. These molecular defects disrupt ocular development and are associated with high myopia and retinal dystrophy.	[224]
	Autosomal recessive microphthalmia	*VSX2* (also known as *CHX10*)	Missense (c.668G>C, p.Gly223 Ala), deletion (c.249delG, p.Leu84SerfsX57) mutations	The missense variant disrupts DNA binding by affecting the conserved CVC motif and is associated with bilateral microphthalmia. Moreover, the deletion results in a truncated VSX2 protein that lacks critical regions (the HD, CVC motif, and C‐terminal).	[225]
	Corneal staphyloma and corneal fistula	*PITX3*	Frameshift mutation (c.640_656dup, p.Gly220Profs*95)	The mutation produces a truncated PITX3 protein and has been associated with unilateral buphthalmos, corneal fistula, and corneal staphyloma.	[226]
Ear and auditory developmental anomalies	Bilateral nonsyndromic microtia	*HOXA2*	Nonsense mutations (c.637A>T (p.Lys213*; c.703C>T, p.Gln235*)	Nonsense mutations cause loss of HOXA2’s transcriptional activation, leading to reduced expression of its target gene, *NKX5‐3* (also known as *HMX1*), which is involved in ear development.	[206]
	Sensorineural hearing impairment (AR and dominant)	*LMX1A*	Missense mutation (c.1106T>C, p.Ile369Thr)	The missense variant in the C‐terminal region disrupts hydrophobic interactions between p.Ile369 and residues in the homeodomain, thereby impairing DNA binding and LMX1A’s transcriptional activation.	[227]
Endocrine and metabolic anomalies	CH	*PAX8*	Eight variants → missense (c.177C>A, p.Ser59Arg; c.208A>G, p.Ser70Gly; c.397C>T, p.Arg133Trp; c.397C>T p.Arg133Trp; c.1334C>T, p.Thr445Met), in‐frame indel (c.396_397delCCinsTT, p.Arg133Trp), in‐frame deletion (c.196_198delTAC, p.Tyr66del), and splicing (c.1276+1G>A) mutations	*PAX8* variants are associated with thyroid dyshormonogenesis despite a normally eutopic gland. Some variants, such as p.Arg133Trp, markedly impair the mutant protein's transactivation activity.	[228]
	CPHD/IGHD	*LHX3*	Missense variants (c.559C>T, p.Pro187Ser; c.658C>A, p.Leu220Met)	The p.Pro187Ser variant destabilizes the HD, disrupting the DNA‐interaction domain, decreasing the protein stability and transcriptional activity, correlating with CPHD, whereas p.Leu220Met is likely benign or associated with IGHD.	[229]
	CPHD	*LHX4*	Missense mutations (c.300G>T, p.Gln100His; c.611G>T, p.Trp204Leu; c.251G>A, p.Arg84His)	The mutations alter the structural conformation of LHX4. In particular, the p.Trp204Leu variant disrupts the hydrophobic core within the homeodomain helix, leading to *LHX4* HI associated with CPHD.	[229]
	CPHD	*HESX1*	Synonymous missense mutation (c.219C>T, p.Ser73Ser)	A synonymous variant in *HESX1* disrupts normal RNA splicing, causing exon 2 to be skipped and exon 1 to splice directly to exon 3, which abolishes the *HESX1* activity.	[230]
	MODY	*PDX1*	Heterozygous missense mutation (c.97C>A, p.Pro33Thr)	The mutation lies within the highly conserved transactivation region of *PDX1* and disrupts its functional integrity. The mutant protein exhibits reduced DNA‐binding affinity and impaired transcriptional activation.	[231]
	MODY9	*PAX4*	Missense mutation (c.487C>T, p.Arg163Trp)	The mutation disrupts the DNA‐binding capacity of the PAX4 protein, causing loss of regulatory control over insulin and glucagon promoters and contributing to dysregulated glucose metabolism and hyperglycemia.	[232]
Cardiac developmental defects and anomalies	Nonsyndromic CHD	*NKX2‐5*	Nonsense mutation (c.342C>A, p.Cys114*)	The nonsense mutation causes PTT, producing a truncated NKX2‐5 protein that impairs its normal function.	[233]
	AF	*PITX2*	Missense mutations (c.309G>C, p.Gln103His; c.370G>A, p.Glu124Lys)	Mutations located within the PITX2c isoform HD impair transcriptional activity, reducing Nppa promoter activation and disrupting the repression of the *SHOX2* promoter. These mutations also disrupt cotransactivation with cardiac cofactors (e.g., Gata4 and Nkx2‐5) and alter the expression of calcium‐regulatory proteins, leading to abnormal calcium handling and the development of atrial fibrillation.	[234]
	SND and AF	*SHOX2*	Missense mutation (c.98C>G, p.Pro33Arg; c.230G>A, p.Gly77Asp)	Heterozygous missense variants in *SHOX2* disrupt its transcriptional activation capacity and downregulate the expression of target genes such as *Bmp4*, which is essential for sinus node formation. These variants are associated with arrhythmogenic phenotypes, particularly SND and AF.	[235]
Kidney, urinary tract, and renal anomalies and diseases	Bilateral renal agenesis	*PAX2*	Synonymous mutation (c.792G>A, p.Gln264Gln)	The synonymous variant affects mRNA splicing, causing exon 6 skipping and introducing a PTC in exon 7. This leads to structural disruption of the HD and generates a truncated protein lacking the transactivation domain. The resulting defect compromises DNA‐binding ability, leading to loss of function.	[236]
	CAKUT and bilateral kidney hypoplasia	*PBX1*	Nonsense mutation (c.992C>A, p.Ser331*)	The mutation occurs at the end of exon 6, a region associated with the HD, resulting in a truncated PBX1 protein.	[237]
Uterine, Müllerian duct, and ovarian anomalies	Septate uterus	*HOXA11*	Missense mutation (c.763C>A, p.Glu255Lys)	The missense mutation occurs within the HD, impairing the DNA‐binding affinity and transactivation function of HOXA11, which impairs Müllerian duct development and leads to a septate uterus due to incomplete medial septum regression.	[238]
	CAUV	*LHX1*	Missense mutation (c.G1108A, p.Ala370Thr)	The mutation lies within the C‐terminal transcriptional activation domain and alters the transcriptional activity of LHX1, disrupting regulation of its downstream target gene, particularly the GSC promoter, thereby affecting urogenital system development.	[239]
	POI	*NOBOX*	Missense mutation (c.131G > T, p.Arg44Leu; c.271G > T, p.Gly91Trp; c.454G > A, p.Gly152Arg; c.1354G > A, p.Asp452Asn; c.349C > T, p.Arg117Trp)	Various *NOBOX* variants exhibit distinct impacts. The p.Gly91Trp and p.Gly152Arg substitutions impair both transcriptional activation and nuclear localization, consistent with a pathogenic loss of function. The p.Asp452Asn and p.Arg117Trp variants also disrupt the transcriptional function of the mutant protein and are consistent with a probable moderate‐risk pathogenic effect.	[240]

Abbreviations: AF: atrial fibrillation; AR: autosomal recessive; CAKUT: Congenital anomalies of kidney and urinary tract syndrome; CAUV: congenital absence of uterus and vagina; CCHS: congenital central hypoventilation syndrome; CH: congenital hypothyroidism; CHD: congenital heart disease; CPHD: combined pituitary hormone deficiency; *EMP*: *enamel matrix protein*; *GSC*: *Goosecoid homeobox*; *HCFP3*: *Congenital facial paresis type 3*; HFGS: hand foot genital syndrome; IGHD: isolated growth hormone deficiency; LMD: Langer mesomelic dysplasia; LWD: Léri‐Weill dyschondrosteosis; *MDUGA*: *Mesomelic dysplasia with urogenital abnormalities*; *MODY*: *Maturity‐onset diabetes of young*; *MODY9*: *MODY type 9*; POI: premature ovarian insufficiency; pSmad5: Phosphorylated Smad5; PTT: premature transcription termination; *SHFM1*: *split‐hand foot malformation type 1*; SND: Sinus node dysfunction; SPD: synpolydactyly; TA: tooth agenesis; TDO: Tricho‐dento‐osseous syndrome.

Heterozygous variants can drive disease either by a GOF or by a LOF that reduces activity below the threshold required for normal physiology. LOF in some genes leads to disease via HI, whereas others may remain clinically unaffected by half‐normal activity [241]. HI denotes a dosage‐sensitive mechanism in which a single functional allele fails to provide sufficient gene product to sustain normal physiology. Consequently, heterozygous LOF variants often manifest as dominant conditions, and HI represents a recognizable subset of rare genetic diseases [16, 241, 242, 243]. In several noncancerous homeobox disorders, diverse variant types have been linked to HI. Robust evidence of HI exists for genes such as such as *PBX1*, *OTX2*, *SHOX*, *PAX6*, and *PITX2* 202]. In *PAX6*, high dosage sensitivity is observed, with most pathogenic variants heterozygous and reducing gene dosage, thereby causing HI and resulting in aniridia with associated features such as corneal opacity, cataract, and glaucoma [16]. Likewise, *PBX1* HI causes syndromic CAKUT, underscoring the critical requirement for proper gene dosage in nephrogenesis [242]. Heterozygous deletion of *OTX2* within the 14q13 region has been identified as the most plausible pathogenic mechanism underlying congenital hypopituitarism and ocular malformations [242]. *SHOX* HI, arising from deletions, duplications, or rarer exonic mutations in *PAR1*, is associated with idiopathic short stature (ISS) and Léri–Weill dyschondrosteosis (LWD); in contrast, increased *SHOX* dosage can contribute to tall stature in sex‐chromosome polysomies (e.g., 47, XXY). Clinically, phenotypic severity in *SHOX*‐related conditions often correlates with hormonal context rather than the mutation class [243].

While the aforementioned mutation types illuminate pathogenic dysregulation across contexts, the phenotypic manifestations of homeobox‐gene dysfunction are often complex, shaped by tissue‐specific expression programs and the position of variants within functional domains. Genotype–phenotype correlations thus provide a critical lens for interpreting how variants in homeobox genes relate to clinical presentations. Even within the same locus, different classes or positions of mutations can yield strikingly variable phenotypes across individuals, reflecting allelic heterogeneity and variable expressivity. Across human cohorts, modest quantitative or qualitative changes can be phenotypically decisive. *PAX6* serves as a well‐studied exemplar, illustrating how clinical phenotypes emerge from the interplay of gene dosage, variant position, and tissue‐specific developmental programs. Beyond its canonical role in eye development, *PAX6* is expressed in neural and nonocular tissues including the forebrain, olfactory system, and endocrine pancreas consistent with its broad developmental functions. Consequently, pathogenic variants that alter *PAX6* dosage or function produce clinically distinct outcomes depending on variant class, domain location, and tissue context. Given that aniridia phenotypes correlate strongly with *PAX6* mutations, comprehensive variant analyses have shown that *PAX6*‐related disorders encompass a wide spectrum of pathogenic variant types, each contributing differently to disease severity [244, 245, 246, 247].

Among reported pathogenic variants, nonsense mutations constitute the largest fraction, followed by frameshift insertions/deletions, splice‐site alterations, and less common classes such as in‐frame indels and C‐terminal extension (CTE) mutations. These variant categories differentially affect *PAX6* protein function and, consequently, the severity of related clinical phenotypes; for example, CTE and other LOF variants tend to yield more severe phenotypes, whereas most missense substitutions are associated with milder forms. Moreover, PAX6‐associated aniridia predominantly arises from heterozygous LOF variants as well as chromosomal rearrangements affecting the 11p13 locus where *PAX6* resides [244, 245, 246, 247]. Together, these observations establish HI as the predominant pathogenic mechanism underlying congenital aniridia (CA) within *PAX6*‐related disorders. Importantly, *PAX6*‐related ocular disorders extend beyond isolated eye malformations. Systemic manifestations in CA frequently accompany ocular involvement, including metabolic disturbances such as thyroid dysfunction, impaired glucose regulation, and hypertension. Consistent with *PAX6* expression in pancreatic tissue, this gene is essential for the development and function of pancreatic β‐cells, so pathogenic variants can contribute to metabolic disorders across life, from persistent hyperinsulinemic hypoglycemia in infancy to an elevated risk of type 2 diabetes in adulthood. Neurological involvement has also been described, including structural brain anomalies in a subset of affected individuals. Isolated foveal hypoplasia (IFVH)‐associated variants are predominantly missense and cluster within specific *PAX6* regions, supporting a genotype–phenotype relationship distinct from that of classic aniridia [244, 245, 246]. Notably, IFVH has been observed in individuals carrying pathogenic *PAX6* variants and typically presents with a fully formed iris. Foveal hypoplasia can co‐occur with aniridia‐associated findings such as cataract or glaucoma. In some cases, biallelic pathogenic variants of *PAX6* (compound heterozygous or homozygous) have been reported and cause profound disruption of ocular development, typically resulting in anophthalmia and severe central nervous system malformations [244, 245, 246]. In addition to *PAX6*, a second example illustrates how biallelic inactivating variants in *NKX6‐2* essential for oligodendrocyte differentiation and regulation of myelin‐associated genes abolish NKX6‐2 function and cause a severe hypomyelinating leukodystrophy, underscoring how dosage and domain context across homeobox genes shape distinct neurodevelopmental outcomes [208].

Beyond biallelic pathogenic mutations that correlate with severity, polyalanine extension mutations represent a distinct mutational mechanism shaping homeobox‐related phenotypes in a length‐dependent manner. The correlation between polyalanine repeat length and disease severity provides a particularly instructive paradigm for understanding genotype–phenotype relationships. Two instructive examples illustrate how these mutation classes converge on clinical expressivity: *HOXD13* and *PHOX2B*. Pathogenic expansions of polyalanine tracts can perturb protein folding and subcellular trafficking in a length‐dependent manner, promoting misfolding and intracellular aggregation of the mutant protein [248, 249]. *HOXD13* harbors diverse mutations causing synpolydactyly (SPD). Comprehensive clinical and molecular analyses have identified three main classes of *HOXD13* mutations associated with SPD: polyalanine expansions, missense mutations, and truncating variants, each linked to distinct molecular mechanisms and phenotypic variability. Polyalanine expansions are the most frequent and are generally associated with the classic, more severe SPD forms compared with missense or LOF variants. Mechanistically, expanded polyalanine tracts promote protein misfolding, cytoplasmic aggregation, and disruption of *HOXD13*’s normal nuclear functions. Missense variants within the homeodomain primarily impair transcriptional activation without causing aggregation. Truncating mutations (nonsense or frameshift) can occur inside or outside the homeobox region; truncations involving the homeodomain abolish DNA binding and transcriptional activity [248]. In congenital central hypoventilation syndrome (CCHS), the vast majority of cases arise from *PARMs* in *PHOX2B*. In healthy individuals, each allele contains about 20 alanines; disease‐associated alleles typically harbor expansions from 24 to 33 alanines. Importantly, individuals with longer polyalanine tracts tend to exhibit severe forms of clinical manifestations of CCHS [249].

##### 3.1.1.1 Epigenetic Regulation of Homeobox Genes in Noncancer Human Disease

Epigenetic modifications have been implicated in a range of noncancerous diseases, highlighting that dysregulation of homeobox gene expression via epigenetic mechanisms can contribute to pathology beyond coding mutations. Epigenetic regulation encompassing DNA methylation, histone modifications, higher‐order chromatin structure, and ncRNAs mediated processes has emerged as a central determinant of gene expression, extending beyond traditional sequence variants [196]. While the interface between homeobox genes and epigenetic regulation has been most extensively explored in oncology, growing evidence demonstrates that similar epigenetic interactions influence noncancerous conditions.

Recent studies across disorders such as endometriosis, type 2 diabetes, and chronic kidney disease show that promoter DNA hypermethylation can repress expression of homeobox genes critical for maintaining tissue‐specific function. Consequently, DNA methylation‐mediated downregulation of homeobox TFs has emerged as a recurring pathogenic mechanism in various noncancerous diseases, whereby epigenetic repression directly impairs tissue physiology. To illustrate this, five representative examples *HOXA10*, *HOXA11*, *HOXD10*, *HOXA5*, and *PDX1* are discussed below to show how promoter hypermethylation of homeobox genes contributes to distinct pathological processes in noncancerous conditions [197, 250, 251, 252]. Among noncancer conditions, endometriosis offers a well‐characterized case where epigenetic regulation of homeobox genes intersects with disease. Across the menstrual cycle, *HOXA10* and *HOXA11* expression is phase dependent: low in the proliferative phase, rising through the secretory phase to a mid‐secretory peak, and remaining elevated in the early decidua after successful implantation. Attenuation of secretory‐phase upregulation of *HOXA10* and *HOXA11* correlates with reduced implantation rates [250]. In contrast, women with endometriosis fail to exhibit the typical cyclic modulation of *HOXA10* and *HOXA11* across menstrual phases, maintaining persistently dysregulated expression throughout the cycle. Multiple independent studies have shown that this disruption of cyclic gene activity is primarily driven by aberrant promoter hypermethylation affecting both genes. Elevated *HOXA10* promoter methylation has been consistently observed in eutopic endometrial tissue of affected women, particularly during the secretory phase, where it directly correlates with marked reductions in gene expression. Moreover, abnormal promoter methylation of *HOXA10* and *HOXA11* has also been reported in other infertility‐associated conditions, such as chronic endometritis and polycystic ovary syndrome, suggesting a shared epigenetic mechanism contributing to endometrial dysfunction across multiple reproductive pathologies [199, 250]. Beyond the reproductive system, similar epigenetic repression of homeobox genes has been observed in other organs. In vitro and in vivo studies have demonstrated that TGF‐β1 stimulation is associated with promoter hypermethylation of *HOXD10*, resulting in suppressed of its expression. Under physiological conditions, HOXD10 functions as a transcriptional repressor of NADPH oxidase 4 (*NOX4)* by directly binding to its promoter; decreased *HOXD10* expression leads to *NOX*4 upregulation, increased ROS production, and contributes to renal tubular injury and fibrosis in chronic kidney disease [251]. Another example is *HOXA5*, which becomes hypermethylated at its promoter during kidney fibrogenesis, leading to loss of expression. *HOXA5* normally represses *JAG1*, thereby restraining *NOTCH* signaling; loss of *HOXA5* expression derepresses *JAG1*, activates *NOTCH* signaling, and promotes fibrogenesis in the kidney [252]. Epigenetic regulation also features in metabolic disease, such as type 2 diabetes. In pancreatic islets from individuals with *T2D*, hypermethylation of distal *HOXA5*‐regulated regions within *PDX1* has been detected and is associated with suppressed *PDX1* expression and attenuated glucose‐stimulated insulin secretion [197].

#### 3.1.2 Neurodegenerative Diseases Associated With Homeobox Gene Dysregulation

Homeobox genes are increasingly implicated as key contributors to NDDs and related disease states, through both genetic variants and epigenetic dysregulation. Under normal physiology, many homeobox genes not only guide early development but also support adult neuronal function, including preservation of neuronal terminal identity, maintenance of neurotransmitter identity, and maintenance of synaptic structure [253, 254]. For example, the LMX1A/B‐driven transcriptional program is crucial for adult midbrain dopaminergic neurons, regulating mitochondrial metabolism and sustaining mitochondrial function [255]. Evidence regarding homeobox gene expression in the adult brain is nuanced: while some studies report that *HOX* genes are undetectable in healthy adult brain tissue, context‐dependent *HOX* activity has been observed in restricted cell populations and disease models, where it appears to help maintain cellular identity. Certain *HOX* TFs have also been implicated in modulating synaptogenesis and supporting neurotransmitter identity [254]. Collectively, dysregulation of homeobox gene expression emerges as a potential pathogenic mechanism linking these genes to neurodegenerative processes. A concrete example is the *HOXB6* locus, where a differentially methylated region containing cg17179862 is hypermethylated in Alzheimer's disease (AD), with elevated methylation in hippocampal tissue correlating positively with tau burden. Table 6 summarizes representative examples of homeobox genes implicated in various neurodegenerative disease related syndromes (NDDS), illustrating the diversity of mechanisms by which their dysregulation can influence neuronal vulnerability and degeneration [256].

**TABLE 6:** The table highlights documented links between specific homeobox genes and major neurodegenerative diseases.

Neurodegenerative disease	Implicated homeobox gene(s)	Expression change in disease (↑/↓)	Functional and mechanistic implications	References
Alzheimer's disease (AD)	*PAX6*	↑ Upregulated	Aβ activates E2F1 and its downstream effector c‐Myb, both of which synergistically transactivate PAX6. PAX6 subsequently upregulates GSK‐3β transcription, leading to increased tau phosphorylation, thereby promoting neuronal death.	[257]
	*HOXA* cluster (esp. *HOXA3*)	↓ Downregulated	Aberrant hypermethylation spanning the HOXA gene cluster, most notably within *HOXA3*, is robustly linked to Alzheimer's disease–related neuropathology, particularly within PFC and STG.	[258]
	*MEOX2*	↓ Downregulated	Reduced *MEOX2* expression impairs angiogenesis and cerebral blood flow, lowers LRP1 levels, and limits Aβ efflux across the BBB, thereby contributing to vascular impairment in AD.	[259]
Parkinson's disease (PD)	*PITX3*	↓ Downregulated	*PITX3* and NURR1 are critically involved in the development and maintenance of mDA neurons. In patients with PD, both genes show significantly reduced expression in PBL samples. Consistently, downregulation of *PITX3* in differentiated mDA neurons leads to progressive neuronal loss.	[260, 261]
	*PBX1*	↓ Downregulated	*PBX1* is involved in the regulation of the maintenance, differentiation, and survival of mDA neurons. In PD, *PBX1* expression is reduced in substantia nigra mDA neurons, which is accompanied by a decrease in its direct target *NFE2L1*. Reduced *NFE2L1* leads to increased vulnerability of mDA neurons to oxidative stress and related damage.	[262]
	*LMX1*	↓ Downregulated	*LMX1A* and *LMX1B* are downregulated in PD, particularly in mDA neurons.	[263, 264]
	*IRX2*	↑ Upregulated	Experimental studies have demonstrated that *IRX2* is upregulated in patient‐derived IOs and NESs carrying the *LRRK2* G2019S mutation, as well as in PD patient‐derived dopaminergic neurons (both *LRRK2* G2019S and sporadic lines), supporting its potential role as a biomarker and linking IRX2 to gut–brain‐axis involvement in PD pathophysiology.	[265, 266]
Amyotrophic lateral sclerosis (ALS)	*PHOX2B*	↓ Downregulated	In vitro studies in human iPSC‐derived motor neurons have demonstrated that TARDBP mutations reduce both axonal *PHOX2B* expression and the stability of its mRNA, leading to diminished axonal resilience and implicating *PHOX2B* downregulation in ALS pathogenesis.	[267]
	*HOXA5*	↑ Upregulated	*C9ORF72* repeat expansion is implicated in both ALS and FTD. Reduced *C9ORF72* expression, particularly transcript variant 2, correlates with upregulation of *HOXA5* and *TTR*, suggesting that their upregulation reflects disease‐linked transcriptional dysregulation across the ALS/FTD spectrum.	[268]
Multiple sclerosis (MS)	*ZEB1*	↑ Upregulated	MS patient‐derived myelin‐reactive Th17 cells exhibit an upregulation of *ZEB1* levels. ZEB1 contributes to disease‐relevant pathogenic inflammation: in CD4^+^ T cells, it is associated with the differentiation of proinflammatory Th1 and Th17 subsets and is involved in the production of IFN‐γ and IL‐17.	[269]
Huntington's disease (HD)	*HOXB7, HOXD4, HOXD10*	↑ Upregulated	Upregulation of *HOXB7, HOXD4*, and *HOXD10* in HD accompanies increased levels of HOX‐related miRNAs.	[270]
	*HOXD1*	↓ Downregulated	*HOXD1* expression is reduced in HD, likely due to its regulation by related miRNAs involved in the disease context.	[270]

Abbreviations: BBB: blood–brain barrier; *C9ORF72*: *Chromosome 9 open reading frame 72*; FTD: frontotemporal dementia; IFN‐g: interferon gamma; IL‐17: interleukin‐17; Ios: intestinal organoids; LRP1: lipoprotein receptor‐related protein 1; *LRRK2*: *Leucine‐rich repeat kinase 2*; mDA: midbrain dopamine; NESs: neuroectodermal spheres; NURR1: nuclear receptor related‐1 protein; PBL: peripheral blood lymphocytes; PFC: prefrontal cortex; STG: superior temporal gyrus; *TARDBP*: *TAR‐DNA binding protein*; TTR: transthyretin.

## 4 A Central Role of Homeobox Genes in Cancer Development

Homeobox genes orchestrate a wide spectrum of biological programs across the lifespan, from embryonic development to maintenance of tissue identity in adulthood [7]. Consequently, precise transcriptional regulation of these genes is essential for tissue homeostasis; even subtle deviations in expression can profoundly reshape cellular behavior and promote tumor progression. Dysregulation of homeobox gene expression whether upregulation or downregulation within the *HOX* clusters has been consistently detected across multiple malignancies, underscoring their fundamental contribution to tumor initiation and progression [3, 7, 191]. Aberrant expression of homeobox genes in cancer cells arises from multiple layers of genetic and epigenetic modifications that disrupt normal transcriptional patterns. These regulatory disturbances may involve genetic alterations such as loss of heterozygosity or gene amplification, alongside epigenetic modification. Across human cancers, and most notably within HOX clusters, epigenetic dysregulation rather than recurrent coding mutations constitutes the dominant axis of homeobox gene alteration. Consequently, these mechanisms act as crucial drivers of tumor initiation, progression, and metastasis through persistent misexpression of key homeobox regulators [3, 191, 270].

In cancer cells, the dysregulation of homeobox genes, especially those within the HOX clusters, has far‐reaching biological consequences, as these TFs rewire core developmental and lineage programs that underlie malignant behaviors [147]. Homeobox genes, particularly those clustered within HOX loci, influence cancer progression by modulating diverse signaling networks, revealing substantial functional plasticity [18]. A prominent example is the EMT, a developmental program hijacked in oncogenesis to enhance tumor cell plasticity. In malignant contexts, EMT promotes dissemination by bestowing epithelial cells with migratory and invasive capabilities, a process closely associated with therapeutic resistance across multiple cancers, including lung carcinoma [271, 272, 273]. Mechanistically, dysregulated homeobox factors intersect with major oncogenic pathways such as Notch, Wnt/β‐catenin, TGF‐β/SMAD, and PI3K/AKT/mTOR, with extensive crosstalk shaping hallmark tumor phenotypes proliferation, invasion, EMT, and angiogenic remodeling. The integrated influence of HOX genes on these signaling cascades underscores their context‐dependent roles in tumor biology and supports their potential as targets for therapeutic intervention [71, 274].

Another critical aspect underscoring the centrality of homeobox genes in cancer is their remarkable dual functionality as both oncogenes and tumor suppressors. Across cancer biology, dysregulation of oncogenes or tumor suppressor genes driven by diverse mutation types or epigenetic mechanisms leading to upregulation or downregulation constitutes a fundamental driver of tumorigenesis [188]. In this landscape, homeobox genes exemplify this dualistic paradigm, displaying either oncogenic or tumor‐suppressive effects depending on cellular context, tissue origin, and developmental stage. Consequently, misexpression of these genes is consistently associated with tumorigenesis in a tissue‐ and stage‐dependent manner. During embryogenesis, those homeobox genes with oncogenic potential may be transiently expressed and contribute to malignant traits when aberrantly re‐expressed in cancer cells, while in fully differentiated adult tissues their expression is often reduced or silenced. Conversely, a subset of homeobox genes remains physiologically expressed in adult tissues but becomes downregulated across various cancers [275]. This dynamic adds a layer of complexity to cancer biology, underscoring that the functional outcome of homeobox genes’ dysregulation is not context‐invariant but contingent on tissue type and developmental state. Illustrative examples of homeobox genes functioning as either oncogenes or tumor suppressors, depending on expression patterns across different tumor contexts, are discussed below. For instance, oncogenic activity is exemplified by *HOXA1*, which is overexpressed in breast cancer and consistently associated with tumor progression and poor prognosis. *HOXA1* is likewise upregulated with tumor‐promoting effects in other malignancies, including PCa, glioblastoma, and head‐and‐neck squamous cell carcinoma (HNSCC) [276]. Similarly, *HOXB5* exhibits oncogenic activity in breast cancer, where its overexpression correlates with increased cellular invasiveness [277]. *HOXB7* is another oncogenic HOX member overexpressed across several cancer types; in breast cancer, high *HOXB7* expression associates with tumor progression and adverse outcomes, and in HNSCC its upregulation correlates with advanced disease stage and poor prognosis [278]. In contrast, certain homeobox genes display tumor‐suppressive functions and are frequently downregulated across cancers. *EMX2* functions as a tumor suppressor, with marked downregulation in lung cancer and esophageal adenocarcinoma; loss of EMX2 enhances cellular proliferation and invasiveness [278, 279]. Similarly, reduced PITX1 expression has been linked to tumor progression in esophageal squamous cell carcinoma (ESCC) [280].

In addition, some homeobox genes exhibit distinct expression patterns across cancers, being upregulated in certain tumor types and downregulated in others. The most striking examples of their functional duality are those that exert opposite roles depending on cancer type and cellular context. For instance, *HOXA9* displays dual functionality across human cancers: its oncogenic activity is evident in both hematologic and solid malignancies. *HOXA9* overexpression has been reported in AML; ∼70% of cases and in acute lymphoblastic leukemia (ALL), where it is strongly associated with disease aggressiveness and poor prognosis. On the other hand, *HOXA9* tumor‐suppressive activity has been observed in cancers such as cutaneous SCC and breast cancer, where its expression is downregulated. Collectively, *HOXA9* can function as either an oncogene or a tumor suppressor, depending on tumor type and cellular context [281, 282, 283]. Among the most striking examples of dual‐function homeobox genes is *NKX3.1*, which plays a pivotal role in prostate development and tumorigenesis. Under physiological conditions, *NKX3.1* promotes epithelial differentiation and acts as a cofactor for the androgen receptor. During PCa progression, its role shifts from tumor suppressor in early stages to exhibiting oncogenic potential in advanced stages [284]. The functional duality of homeobox genes largely depends on multiple factors, including tumor type, the developmental stage of the malignancy, and the surrounding tumor microenvironment (TME). These context‐dependent parameters collectively shape whether a given homeobox gene acts as an oncogene or a tumor suppressor.

In addition to dysregulation of homeobox genes caused by genetic and epigenetic alterations, multiple extrinsic factors influence their expression and function. Among these, the tumor microenvironment (TME) exerts a profound regulatory impact on how these genes contribute to tumor initiation and progression. The TME is a highly complex and dynamic milieu whose composition varies across tumor types, yet consistently includes cancer‐associated fibroblasts, diverse immune cells, vasculature, and extracellular matrix (ECM). Each component can modulate the expression and function of homeobox genes through a range of integrated mechanisms [285, 286]. Among these, the ECM plays a particularly critical role in tumor progression. ECM remodeling not only supports angiogenesis but also promotes tumor cell motility and the induction of EMT, thereby increasing malignant potential and the likelihood of metastasis in solid tumors [286, 287, 288]. EMT is well known for its critical role in the progression of various cancers, driving metastasis and resistance to conventional therapies, particularly in solid tumors. Distinct signaling molecules, such as Wnt, TGF‐β, and BMP, also orchestrate various types of EMT, with each contributing to specific EMT types during processes such as embryonic development, tissue remodeling, and cancer progression. EMT is a central driver of cancer progression, metastasis, and therapeutic resistance, particularly in solid tumors. Distinct signaling pathways such as Wnt, TGF‐β, and BMP orchestrate various EMT types that operate during embryogenesis, tissue remodeling, and cancer progression. EMT entails extensive reprogramming of gene expression, as well as remodeling of cellular morphology and metabolism, enabling epithelial cancer cells to acquire migratory capabilities and more resilient phenotypes. A diverse set of TFs orchestrates EMT, with several core EMT‐TFs acting as central regulators that influence both their own expression and that of other EMT‐TFs. Notably, the *ZEB1* and *ZEB2* zinc‐finger E‐box‐binding homeobox proteins are regarded as important core EMT regulators [289, 290, 291]. Consequently, EMT shapes the expression and regulatory dynamics of various homeobox genes across multiple cancer types, contributing to tumor progression and metastasis. For instance, in esophageal cancer*, forkhead box C1* (*FOXC1*) modulates *ZEB2* expression through its association with the pioneer factor *PBX1*, thereby promoting EMT [292]. In addition, *HOX* cluster‐linked lncRNAs (*HOX*‐*lncRNAs*) have emerged as key regulators of EMT, modulating transcriptional networks that drive tumor progression and metastatic potential [293].

Beyond their involvement in EMT, homeobox genes significantly influence other TME‐associated hallmarks of cancer, particularly angiogenesis. Angiogenesis is a fundamental mechanism by which the TME promotes malignant progression, and multiple *HOX* family members act as pivotal regulators of this process. *HOX* genes have long been implicated in tissue patterning and vascular development, in part by regulating ECs function. Their expression has been associated with proliferation and migration of vascular smooth muscle cells, atherosclerotic plaque formation, and remodeling of cardiac tissue after injury [294, 295, 296]. Emerging evidence also indicates that dysregulation of specific *HOX* genes contributes to tumorigenesis by promoting angiogenesis within tumors. Conversely, *HOX* gene dysregulation has been linked to cardiovascular diseases such as atherosclerosis, heart failure, and arrhythmias, highlighting the broader relevance of *HOX* pathways to tissue remodeling and pathology [294, 295]. In cancer, angiogenesis is tightly coordinated by a balance of pro‐ and antiangiogenic factors, and *HOX* genes can influence this balance through context‐dependent regulation of endothelial and stromal cell behavior as well as interactions with other TME components. Notably, angiogenesis is a critical process in tumor progression as it involves in cancer invasion, and metastasis. This process is modulated by a range of pro‐ and antiangiogenic factors [137, 296, 297]. One of the well‐established *HOX* regulators of angiogenesis is *HOXA9*. *HOXA9* promotes angiogenesis by interfacing with multiple signaling pathways and TFs. Moreover, accumulating evidence shows that *HOXA9* overexpression enhances angiogenic activity while *HOXA9* downregulation markedly impairs these processes. This dual role physiological in some contexts and pathological in cancer highlights the importance of *HOXA9* in cancer biology. Dysregulation of *HOXA9*, whether by aberrant DNA methylation or other mechanisms, can contribute to angiogenesis deregulation, a hallmark of cancer progression and metastasis [281, 298]. In humans, *HOXA9* also drives EC migration, a key step in vasculature formation, in part by upregulating *EphB4*, a receptor tyrosine kinase involved in blood vessel development and maturation. *EphB4* is frequently overexpressed in various cancers and supports tumor growth by enhancing angiogenesis; its expression levels correlate with tumor growth and differentiate status in cancers such as lung cancer, underscoring its oncogenic potential [296, 297, 299, 300]. Other *HOX* family members linked to angiogenesis and dysregulation in cancer include *HOXB3*, *HOXB9*, and *HOXD3*, which have been implicated in regulating angiogenic processes. *HOXB9*, in particular, is often overexpressed across solid tumors (e.g., lung adenocarcinoma) and is associated with increased proangiogenic factor expression and activation of proangiogenic signaling cascades. This mechanistic link is clinically relevant because angiogenesis is a major route through which tumors resist antiangiogenic therapies, suggesting that *HOXB9* angiogenic programs can contribute to therapeutic resistance [137].

### 4.1 Cancer‐Associated Epigenetic Modulation of Homeobox Genes

As described, extensive research over recent decades has established epigenetic modifications as critical contributors to the onset and progression of numerous diseases. These alterations extend beyond cancer to a range of noncancerous and NDDs, underscoring their broad impact on human health. Epigenetic mechanisms regulate gene expression without altering the DNA sequence, thereby influencing disease initiation and progression, including cancer. The major modalities include DNA methylation, histone acetylation and methylation, and ncRNA‐mediated regulation, all of which can silence tumor‐suppressor programs or activate oncogenic circuits. Consequently, these modifications can drive tumorigenesis and shape cancer development and metastasis. Aberrant DNA methylation patterns at CpG sites can silence tumor suppressor genes or aberrantly activate oncogenes. A particularly relevant area in cancer is the dysregulated expression of ncRNAs within the HOX cluster networks [71, 301, 302]. Abnormal methylation of homeobox genes constitutes a crucial area of study in epigenetics, given its impact on regulatory roles and cancer prognosis.

#### 4.1.1 Methylation‐Mediated Silencing and Activation of Homeobox Genes in Cancer

A large body of evidence indicates that aberrant DNA methylation and sequence mutations can dysregulate multiple homeobox genes, promoting cancer initiation and progression by disturbing control of cell proliferation, apoptosis, differentiation, and angiogenesis. Across many cancers, particularly solid tumors, methylation abnormalities are a major contributor to *HOX* genes’ misexpression. Within the four HOX clusters, numerous genes show recurrent dysregulation with functional consequences for tumorigenesis [149, 191]. As discussed, homeobox gene misexpression especially within HOX clusters drives tumorigenesis in a context‐dependent fashion, with individual genes acting as oncogenes or tumor suppressors depending on context. Aberrant DNA methylation is a key mechanism governing this regulation [74, 147]. DNA methylation, mediated by DNA methyltransferases (*DNMTs*; notably *DNMT1* and *DNMT3A/3B*), involves adding a methyl group to cytosine, influencing gene expression by recruiting repressive proteins or hindering TF binding. This process is dynamic, involving de novo methylation and demethylation that contribute to tissue‐specific gene regulation and the establishment of stable methylation patterns in differentiated cells [283, 303]. In mammals, DNA methylation at the C5 position of cytosine can impact gene regulation by recruiting repressive proteins or inhibiting the binding of TFs. This process is highly dynamic, encompassing both de novo methylation and demethylation events, and contributes to tissue‐specific gene regulation and the establishment of stable methylation patterns in differentiated cells [301, 302]. Ongoing research is dedicated to unraveling the intricate mechanisms underlying DNA methylation and its profound impact on gene regulation, development, and the manifestation of various disease states [301, 304, 305]. Notably, aberrant DNA methylation patterns have important roles in cancer onset, progression, and metastasis [306].

Although aberrant DNA methylation is widespread in cancer, its impact on transcription is region and context dependent, not uniformly silencing [71]. In general, CpG‐island promoter hypermethylation represses transcription by recruiting methyl‐CpG‐binding proteins (e.g., MeCP2), thereby reducing transcription‐factor and RNA polymerase II (RNAP II) access [307]. For instance, *HOXD10* is frequently hypermethylated at its promoter and transcriptionally silenced in endometrial cancer (EC), where its loss correlates with tumorigenic progression, particularly in endometrial adenocarcinoma [308]. Similarly, promoter hypermethylation of *HOXA5* has been reported in several malignancies, including CRC, non‐small‐cell lung cancer (NSCLC), and chronic myeloid leukemia (CML); notably, hypermethylation of *HOXA4/HOXA5* is strongly associated with imatinib resistance in CML patients [309, 310]. In breast cancer, *HOXD13* promoter methylation is a common event and predicts worse overall survival (OS), supporting its value as a prognostic [311]. Collectively, aberrant promoter methylation of homeobox genes is linked to disease progression, prognosis, and patient outcomes across diverse cancer types. In line with this, promoter CpG island hypermethylation is a well‐established driver of neoplastic transformation, enforcing stable transcriptional silencing of tumor suppressor genes and enabling oncogenic programs to proceed [306]. *HOXC10* serves as a clear example of hypomethylation‐mediated activation within two different types of cancer, with promoter CpG‐island hypomethylation reported in NSCLC and hypomethylation at its CpG sites within the first intron in gastric cancer, accompanying *HOXC10* overexpression and tumor progression [312, 313].

Although aberrant promoter CpG‐island hypermethylation typically represses gene expression, methylation within gene‐bodies often tracks with active transcription and has been linked to tumor initiation and progression an observation widely referred to as the “DNA methylation paradox” [307]. This paradox is prominently observed at homeobox genes; the pan‐cancer integration of analyses has shown that “DNA methylation canyons,” large under‐methylated tracts, tend to become hypermethylated in various types of cancer, and this kind of hypermethylation is strongly associated with upregulated expression in ≈43% of homeobox genes, which are significantly enriched for oncogenes. For illustration at homeobox loci, gene‐body “canyon” hypermethylation at *DLX1* coincides with aberrant overexpression in bladder urothelial carcinoma (BLCA), uterine corpus endometrial carcinoma (UCEC), lung SCC (LUSC), and LUAD; similarly, *POU3F3* harbors a hypermethylated gene‐body canyon with overexpression in LUSC, BLCA, UCEC, supporting that gene‐body hypermethylation can increase expression at these loci [314]. As a whole, the complexity of these methylation patterns underscores the potential for tissue‐specific therapeutic strategies targeting the epigenetic dysregulation of homeobox genes in cancer [312].

#### 4.1.2 The Interplay Between Homeobox Genes’ Expression, *lncRNAs*, and *miRNAs* in Cancer

The human genome predominantly comprises ncRNAs, which account for approximately 90% of its sequence, while only about 2% of the genome is dedicated to protein‐coding. Notably, ncRNAs are broadly categorized into small ncRNAs (sncRNAs) and lncRNAs [315]. In various biological sources, sncRNAs are also defined as ncRNAs with fewer than 200 nucleotides in length and are categorized into different classes, including *miRNAs*, tRNA‐derived stress‐induced small RNAs (tiRNAs), small interfering RNA (siRNA), small nucleolar RNAs (snoRNAs), and PIWI‐interacting RNAs. Collectively, these different classes of sncRNAs are involved in modulating gene expression through various mechanisms at multiple levels. Moreover, they are known to contribute to epigenetic regulation [316]. Notably, *miRNAs*, the most prevalent class of ncRNAs, are compromised approximately 19–25 nucleotides in length. They are participated in posttranscriptional gene regulation by binding to the 3′‐untranslated regions (3′‐UTRs) of their specific target mRNAs. This interaction typically leads to either mRNA degradation or translational repression. *HOX* genes are remarkable targets of *miRNAs*. A notable connection exists between *miRNAs* and these genes regulation, as some *miRNAs* are located within HOX gene clusters [317, 318]. Notably, *miRNAs* regulate approximately 30–50% of human protein‐coding genes, underscoring their extensive involvement in biological and pathological processes, including carcinogenesis. Significantly, their expression levels are dynamically regulated across various cell types and developmental stages, and aberrations in their expression have been strongly linked to disease progression. Therefore, dysregulation of miRNAs function or expression, whether upregulation or downregulation, are particularly associated with various cancers, including lung cancer. In lung cancer, several *miRNAs* have been identified as key regulators [317, 319, 320]. For example, *miR‐21*, often overexpressed in NSCLC, is associated with tumor progression, whereas *miR‐128*, which acts as a tumor suppressor, is frequently downregulated in NSCLC. These findings underscore the critical roles *miRNAs* play in cancer development and their potential as therapeutic targets or biomarkers [321]. In contrast, *lncRNAs* are characterized by having more than 200 nucleotides in length. They are well known for their lack of protein‐coding potential and exhibit diverse characteristics and functionalities, allowing further classification into subclasses such as sense *lncRNAs*, promoter‐upstream *lncRNAs*, intergenic *lncRNAs*, and bidirectional *lncRNAs* [315, 322]. *LncRNAs* employ diverse and complex mechanisms to influence cellular processes, such as transcriptional regulation, regulation of chromatin structure, RNA splicing, gene imprinting, and cell proliferation. While the precise functions of many *lncRNAs* remain elusive, their roles in gene expression and transcriptional regulation are widely reported. These functions occur through interactions with DNA, mRNA, miRNA, and proteins. For instance, *lncRNAs* can either activate or suppress transcription through various mechanisms. Interestingly, *lncRNAs* share structural analogies with mRNAs, as both are transcribed by RNAP II [322, 323, 324]. Despite their inability to encode proteins, certain *lncRNAs* possess features such as an N7‐methylguanosine (m^7^G) at the 5′ cap and a 3′ polyadenylated (polyA) tail. Notably, nearly half of all *lncRNAs* contain polyA tails, and almost all undergo splicing and accumulate in the cytoplasm, further enhancing their functional versatility. These attributes underscore the intricate mechanisms through which *lncRNAs* regulate transcription [323]. Specifically, one of the primary mechanisms by which *lncRNAs* regulate target gene expression is through their interaction with *miRNAs*, a process also widely reported in various types of cancer. Functionally, *lncRNAs* interact with *miRNAs* via multiple mechanisms, significantly influencing gene expression. A key role of *lncRNAs* is to act as competitive endogenous RNAs (ceRNAs), often described as exhibiting “miRNA sponge” activity by binding to *miRNAs* at specific complementary sites. As ceRNAs, by binding to *miRNAs* at specific complementary sites, *lncRNAs* limit miRNA availability for interaction with target mRNAs. This reduction in miRNA regulatory influence ultimately leads to the upregulation of the target genes. Consequently, this regulatory interaction establishes a complex network involving *lncRNAs*, *miRNAs*, and their targets, emphasizing the multifaceted role of *lncRNAs* in gene expression regulation and their potential implications in tumorigenesis [324]. In addition, *lncRNAs* play integral roles in epigenetic modifications, particularly by recruiting chromatin‐modifying complexes through their interactions with DNA, RNA, and related proteins [322, 325].

In various types of cancer, epigenetic modifications, particularly DNA methylation, play a significant role in regulating the expression of ncRNAs, including both *miRNAs* and *lncRNAs*. Moreover, interactions between epigenetic mechanisms and miRNA regulation critically contribute to the initiation and progression of human cancers. Such alterations in their expression can substantially affect the development and progression of lung and other cancers. For example, the methylation status of CpG islands within promoter regions determines miRNA transcription, with hypomethylation enhancing and hypermethylation suppressing miRNA expression, respectively. Similarly, DNA methylation is one of the primary factors regulating lncRNA expression; abnormal hypermethylation of lncRNA promoters can lead to their silencing. Moreover, *lncRNAs* themselves can modulate methylation patterns by interacting with *DNMTs* and demethylases, directing these enzymes to specific promoter regions to regulate gene expression. Therefore, methylation‐related *lncRNAs* play a key role in shaping tumor biology, with certain *lncRNAs* modulating DNA methylation to regulate gene expression. For instance, aberrant expression of lncRNA ELF3‐AS1 is associated with hypermethylation of *miR‐212*, which contributes to increased NSCLC cells invasion. Similar methylation‐dependent regulatory mechanisms have also been reported in other malignancies. For instance, in gastric cancer, loss of TFF1 expression has been linked to promoter hypomethylation and subsequent activation of the *HOXA10*/miR‐196b‐5p axis, thereby enhancing cell proliferation and invasion. It should be noted that, the bidirectional interplay between *lncRNAs* and DNA methylation underscores their significant role in epigenetic regulation and their contribution to tumor progression, growth, and patient outcomes in lung cancer [326, 327, 328]. As motioned above, in lung cancer, DNA methylation regulates the expression of specific *lncRNAs*, either silencing or activating them. For instance, hypomethylation of the *MIR503HG* promoter in LUAD leads to its upregulation, promoting tumor proliferation through the lncRNA *MIR503HG*/*SNHG17*/miR‐330–3p axis. Conversely, hypermethylation‐induced downregulation of lncRNA lung cancer immune cell infiltration associated RNA (LCIIAR) suppresses LUAD metastasis, highlighting the dual role of methylation in lncRNA‐mediated cancer regulation. In lung cancer metastasis, evidence also suggests that exosomal *lncRNAs*, such as *Ubiquitin‐Fusion Protein 1* (*UFC1*), have been identified as contributors to these processes. Consequently, *lncRNAs* are known to exhibit oncogenic and tumor‐suppressive functions, highlighting their diverse roles in cancer biology [327, 329, 330]. In recent years, several *lncRNAs* have been identified as key players in lung cancer, including cancer‐associated *LncRNA‐1* (*SCAL1*), antisense ncRNA in the INK4 Locus (*ANRIL*), *UFC1*, metastasis‐associated lung adenocarcinoma transcript 1 (*MALAT1*), and *HOXA* transcript antisense RNA, myeloid‐specific 1 (*HOTAIRM1*). Notably, a subset of *lncRNAs* is closely associated with HOX gene clusters such as HOTAIR, *HOTAIRM1*, *HOXA11 antisense RNA (HOXA11‐AS)*, HOXA Cluster Antisense RNA 3 (HOXA‐AS3), and *HOXD cluster antisense RNA 1 (HOXD‐AS1)*. For instance, *HOTAIR*, transcribed from the antisense strand of the HOXC gene cluster on chromosome 12, is particularly well known for its significant upregulation in lung cancers, especially NSCLC. Its strong oncogenic properties make *HOTAIR* a critical regulator of lung cancer progression and metastasis [322, 329, 330]. While HOX‐linked *lncRNAs* such as *HOTAIR* act predominantly as oncogenic drivers in lung cancer, their functions are not uniformly protumorigenic across tissues. This context dependence is exemplified in breast cancer, where diminished *HOTAIRM1* expression associates with more aggressive phenotypes, indicating a tumor‐suppressive role. Loss of *HOTAIRM1* drives breast‐cancer cells to proliferate, form more colonies, and invade, whereas restoring its expression suppresses these malignant traits. Consistently, reduced *HOTAIRM1* levels predict worse outcomes, suggesting it as a potential therapeutic lever in breast invasive carcinoma [331]. Therefore, HOX‐cluster associated *lncRNAs* constitute a context‐dependent epigenetic axis in cancer.

### 4.2 The Interplay of Homeobox Genes and the *Wnt* Signaling Pathway Across Cancers With Emphasis on Lung Cancer

Homeobox genes participate in a wide array of signaling networks that regulate developmental physiology, and under pathological conditions particularly in human cancers—they engage in multiple oncogenic cascades. A broad set of homeobox TFs functionally interacts with core oncogenic pathways, including, Wnt/β‐catenin, TGF‐β/Smad, PI3K/Akt, MAPK/ERK, NF‐κB, and JAK/STAT, in development and tumorigenesis across contexts [87]. Among these, Wnt signaling stands out as central to many fundamental cellular processes, and its dysregulation is a common driver of tumorigenesis. In CRC, aberrant Wnt activity was early linked to disease pathogenesis, and dysregulated Wnt/β‐catenin signaling has since been recognized as a hallmark in a broad range of malignancies. Mutations in pathway components such as adenomatous polyposis coli (*APC*) or *β‐catenin* can sustain constitutive activation, promoting unchecked proliferation, survival, and metastatic potential. Beyond CRC, aberrant Wnt signaling has been implicated in liver, breast, gastric, and other cancers, underscoring its wide oncogenic relevance. Consequently, therapeutic strategies targeting Wnt receptors, core mediators, or downstream effectors are actively explored as potential anticancer approaches. The *Wnt* network does not operate in isolation; it engages in extensive crosstalk with pathways such as Hh, *Not* Hh, Notch, Hippo, TGF‐β/Smad, NF‐κB, and PI3K/AKT, shaping both normal development and tumor behavior. Understanding these interconnections is crucial for designing effective interventions and for interpreting cancer biology in translational contexts [165].

Accordingly, the Wnt signaling pathway remains one of the most extensively studied to date, though its full functionality is not yet fully understood. This critical pathway was first identified in 1982 with the discovery of the int1 (*Wnt1*) gene's role in tumor growth [332]. The Wnt/β‐catenin signaling cascade plays a pivotal part in a wide range of physiological processes. It regulates key functions like embryonic development, immune responses, cell growth, and death, as well as maintaining homeostasis in vital organs such as the lungs, intestines, and liver. This pathway is involved in the broadest array of biological activities, including cell proliferation, differentiation, organogenesis, regeneration, and is associated with conditions like neurodevelopmental disorders and cancer [159, 332, 333, 334, 335]. In the lungs, the Wnt/β‐catenin signaling pathway plays a pivotal role that extends beyond maintaining homeostasis. It is essential for regulating lung epithelial SCs and facilitating their differentiation into specialized epithelial cell types. For example, research indicates that this pathway is probably associated with the regulation of epithelial differentiation in lung resident MSCs (LR‐MSCs). During lung development, Wnt/β‐catenin signaling governs critical processes, particularly the precise fate determination of progenitor cells. Beyond its developmental roles, the pathway is also instrumental in tissue remodeling and regeneration under stress or damage. However, dysregulation of this pathway through various mechanisms can impair these functions, leading to pathological conditions in the lungs, including lung cancer [301, 336]. Therefore, given the paramount significance of this pathway in the lung cells, it is evident that this pathway's abnormal activation could potentially be the underlying cause of various lung diseases, particularly in lung carcinogenesis [333]. Hence, recent research on lung cancer has demonstrated a significant correlation between the activation of the Wnt/β‐catenin pathway and an increased tumor mutational burden in NSCLC. This finding underscores the strong association between *Wnt/β‐catenin* pathway dysregulation and NSCLC tumor development, highlighting its potential impact on the progression of this cancer type [301, 337, 338]. Given the crucial role of the Wnt/β‐catenin signaling pathway in cancer development and progression, particularly through its interaction with the TME, it is vital to explore its regulation at the molecular level [339]. A comprehensive understanding of the various proteins involved in regulating the Wnt signaling pathway is essential for elucidating its impact on cancer progression, including lung cancer. This pathway comprises various proteins and is primarily subdivided into canonical and noncanonical pathways. The Wnt/β‐catenin pathway, commonly referred to as the canonical pathway, is characterized by the translocation of cytoplasmic β‐catenin into the nucleus. Key proteins involved in the Wnt signaling pathway include Wnt ligands, Frizzled receptors, lipoprotein receptor‐related protein 5 and 6 (LRP5/6), Dishevelled 1 (Dvl1), Glycogen Synthase Kinase 3 Beta (GSK3β), APC, Axin, *β‐catenin*, and T‐cell factor/lymphoid enhancer factor (TCF/LEF) family transcriptional regulators [301].

**FIGURE 3:**
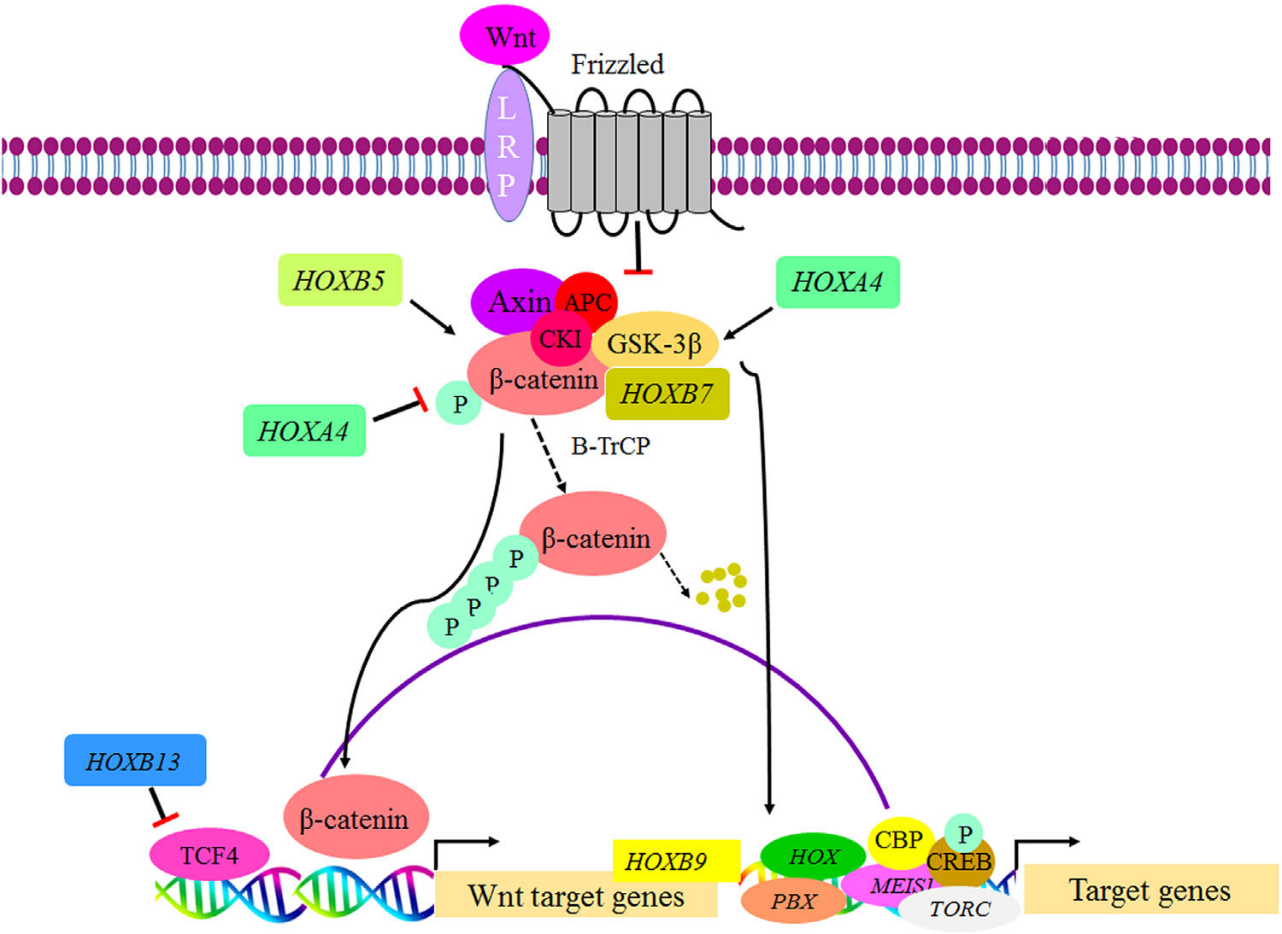
The *Wnt/β‐catenin* signaling pathway and its bidirectional crosstalk with *HOX* genes. In the active state (left), Wnt ligand binding disrupts the *β‐catenin* destruction complex, leading to β‐catenin stabilization and nuclear translocation. Nuclear *β‐catenin* then associates with *TCF/LEF* factors to activate transcription of target genes, including *HOX* genes. HOX proteins, in turn, modulate the pathway through feedback regulation: HOXA4 downregulates *β‐catenin* expression, while *HOXB5* upregulates it. In the inactive state (right), the destruction complex remains active, leading to *β‐catenin* phosphorylation, ubiquitination by *β‐TrCP*, and proteasomal degradation. This schematic highlights the key regulatory loop between Wnt signaling and HOX‐mediated transcriptional control.

Various genes within HOX family exhibit significant involvement in the Wnt signaling cascade. For instance, *HOXB5* has been shown to upregulate the expression of β‐catenin, a key effector in the pathway. Conversely, *HOXA4* appears to decrease β‐catenin levels while increasing the protein and mRNA abundance of GSK3β, a negative regulator of the pathway. Interestingly, the stability of β‐catenin has been linked to the expression of *HOXB9*, which is itself a target gene of the Wnt/TCF signaling axis. Additionally, HOXB7 has been found to form a physical complex with β‐catenin, potentially modulating its activity. Conversely, HOXB13 has been reported to downregulate the expression of *TF 4* (*TCF4*), a crucial TF in canonical Wnt signaling [87]. Furthermore, GSK3β has been shown to promote the conditional association of the transcriptional factor cyclic amp‐responsive element‐binding protein (CREB) and its coactivators with the HOX cofactor MEIS1. This interplay is thought to facilitate HOX‐mediated transcriptional regulation and potentially contribute to tumorigenesis. It should be highlighted that the role of HOX proteins in modulating Wnt signaling can be complex, with some HOX factors promoting cancer progression while others may inhibit it. Further research is needed to fully elucidate the intricate crosstalk between these two pivotal developmental pathways (shown in Figure 3) [87]. Thus, HOX proteins, play essential roles in mediating the Wnt signaling pathway [71, 87].

Among the members involve in Wnt signaling pathway, it is crucial to highlight that β‐catenin acts as a pivotal effector. Once inside the nucleus, β‐catenin can interact with T‐cell factor/lymphoid enhancing factor (TCFs/LEF) TFs, leading to the initiation of the TCFs/LEF transcription complex. Therefore, this pathway ultimately involves the regulation of the expression of target genes. On the other hand, the noncanonical pathways, such as the Wnt/calcium pathway, occur independently of β‐catenin and are more closely related to cell differentiation [159, 333]. As a consequence, the Wnt signaling pathway is crucial in governing gene expression regulation, notably including the *HOX* genes’ expression. In contrast, elevated levels of β‐catenin are directly correlated with the enhanced activation of transcription processes, leading to the overexpression of key genes such as *cyclin D1* and *c‐MYC* [159, 333]. Hence, the overexpression of *Cyclin D1* and *c‐Myc* has been detected in various cancers, including lung cancer, and they are deeply implicated in the process of carcinogenesis [340]. Cyclin D1 plays a critical role in cell cycle regulation, particularly in facilitating the progression from the G1 phase to the S phase. By activating cyclin‐dependent kinases 4 and 6 (CDK4/6), Cyclin D1 ensures proper cell cycle advancement. Due to its critical role in cell cycle regulation, the overexpression of Cyclin D1 is directly associated with neoplastic growth. Such dysregulation has been observed in various types of cancer, especially lung cancer. Existing evidences have consistently detected *Cyclin D1* overexpression in a significant proportion of NSCLC cases, with reported frequencies ranging from approximately 60 and 76% [341, 342]. Similarly, *c‐Myc*, a prominent proto‐oncogene, encodes a TF that plays a pivotal role in regulating the expression of genes essential for fundamental cellular functions. By selectively amplifying the expression of its target genes, *c‐Myc* influences diverse processes such as cell growth, proliferation, differentiation, and division [341, 343, 344]. Additionally, it is a key regulator of apoptosis and angiogenesis. The overexpression of *c‐Myc* has been observed in multiple types of cancers, including lung cancer, where it is strongly associated with tumorigenesis and cancer progression. The involvement of *c‐Myc* in relationship with aggressiveness is reported across numerous human malignancies. In NSCLC, *c‐Myc* overexpression has been reported with significant variability, ranging from 18 to 91% across different studies. For example, a study analysis of 30 NSCLC samples found that 37% exhibited *Myc* overexpression, reflecting inconsistencies in the existing data [343, 345, 346]. These relationships underscore the critical importance of β‐catenin in regulating gene expression linked to cancer progression. As a consequence, dysregulation of the Wnt/β‐catenin signaling pathway is directly associated with a range of cancers. Notably, in various tumor types, it has been observed that the aberrant and excessive activation of the Wnt/β‐catenin signaling pathway is associated with the promotion of CSCs, which is highly associated with tumorigenesis, tumor metastasis, and chemotherapy resistance such as cisplatin, docetaxel, and radiotherapy, and Wnt inhibitors may restore sensitivity [333, 347].

Some *HOX* genes appear to impact lung cancer development by modulating the Wnt signaling pathway, particularly the Wnt/β‐catenin axis, which is commonly disrupted in various types of cancer. Research indicates that HOX proteins interact with β‐catenin and play a crucial role in regulating this pathway, underscoring their significance in cancer progression. Notably, specific HOX proteins, such as HOXA4, HOXA13, and HOXB5, have been directly implicated in the regulation of the Wnt/β‐catenin pathway, particularly in lung cancer [87, 348]. These proteins play crucial roles in cellular differentiation, proliferation, and tumor suppression. A brief overview of their functions and interactions with this pathway is provided below. *HOXA4* exhibits dual roles, functioning either as an oncogene or a tumor suppressor, depending on the cancer type. Abnormal *HOXA4* expression has been observed in various cancers, particularly in lung and cervical cancers. In lung cancer tissues, research shows that *HOXA4* levels are significantly reduced, a change associated with more aggressive tumor characteristics, such as larger tumor size and also poorer patient outcomes. This reduction indicates a critical tumor‐suppressive role for *HOXA4*. Specifically, *HOXA4* primarily acts as a tumor suppressor, largely through its interaction with the Wnt/β‐catenin signaling pathway in lung cancer. Notably, *HOXA4* exerts its tumor‐suppressive effects by enhancing the transcription of GSK3β, a key regulator of the Wnt pathway. GSK3β facilitates the phosphorylation and subsequent degradation of β‐catenin, thereby reducing its levels within the pathway. Additionally, *HOXA4* overexpression directly inhibits β‐catenin expression by binding to the promoter of its gene (*CTNNB1*). This suppression further diminishes the activity of key downstream target genes, including *Cyclin D1* and *c‐Myc*. Through these mechanisms, *HOXA4* effectively disrupts the Wnt/β‐catenin pathway, leading to reduced cell proliferation and metastasis in lung cancer [349, 350]. In contrast, studies have demonstrated that *HOXA13* plays an oncogenic role in lung cancer. Experimental evidence from both cell line studies and patient samples confirms that *HOXA13* is significantly overexpressed in NSCLC tissues compared with healthy samples, suggesting its involvement in cancer progression. In lung cancer, a key mechanism underlying HOXA13's oncogenic activity involves the transcriptional regulation of both the Wnt/β‐catenin and p53 signaling pathways. *HOXA13* activates the Wnt/β‐catenin while inhibiting p53 signaling pathways, thereby promoting the tumor progression [351]. Consequently, the overexpression of *HOXA13* expression not only reduces the expression of *p53* itself but also directly leads to downregulation of p53 downstream target genes, such as *P21* and protein *Bcl‐2‐associated X protein (Bax*). As a result, *HOXA13* disrupts essential processes such as apoptosis. It should be noted that in normal condition, the p53 protein is one of the most important TF that has serious responsibility in regulation of various process, particularly regulating cell cycle arrest and promoting apoptosis. It should be noted that the *p53* pathway encompasses a network of genes and interacts with several other pathways such as the Wnt pathway, the cyclin‐CDK pathway, and the *p38* MAP kinase pathway [351, 352]. Specifically, p53 also involvement in two critical pathways, namely, *p53/p21/p27* and *p53/antiapoptotic protein B‐cell lymphoma‐2 (BCL‐2)/Bax*, are essential for regulating apoptosis as well as cell cycle progression, especially during the G2/M phase. As a consequence, disrupting the *p53* pathway can directly correlated with upregulation of cell proliferation in cancer [351, 352]. As mentioned earlier, *HOXA13* activates the Wnt/β‐catenin signaling pathway, which in turn increases the expression of genes such as *CTNNB1*, *MYC*, and *Cyclin D1*. These genes are associated with enhance tumorigenesis and cancer progression. Consequently, *HOXA13* overexpression significantly contributes to promoting cancer cell metastasis [351, 353]. Findings also have demonstrated a strong association between the expression of *HOXB5* and tumor development in multiple cancer types [348]. *HOXB5* plays a crucial role in modulating the Wnt/β‐catenin signaling pathway, and its association with β‐catenin has been extensively documented in various cancers, including lung, breast, HNSCC, as well as gastric cancers. *HOXB5* significantly impacts cancer progression by directly interacting with the *CTNNB1* promoter, which encodes β‐catenin. This interaction stimulates β‐catenin transcription and upregulates downstream targets such as *cyclin D1* and *c‐Myc*, enhancing the invasive potential in gastric cancer, breast cancer, and particularly NSCLC [71, 354]. Experimental studies on NSCLC cell lines also show that knocking down *HOXB5* reduces β‐catenin levels, which in turn decreases the expression of *cyclin D1* and *c‐Myc*. This downregulation impairs NSCLC cell proliferation and invasion, emphasizing the crucial role of *HOXB5* in regulating the Wnt/β‐catenin pathway during lung cancer progression [277, 354, 355]. Similarly, the Wnt/TCF signaling pathway plays a crucial role in the progression and metastasis of LUAD, particularly through its target genes *HOXB9* and *lymphoid enhancer binding factor 1* (*LEF1*). Therefore, aberrant activity of this pathway enhances the metastatic potential of tumor cells, enabling colonization of distant organs such as the brain and bones. *HOXB9*, a key target gene of *TCF4*, is notably upregulated in lung cancer and has been strongly linked to enhanced invasive and metastatic potential in malignant cells [356, 357]. Likewise, LEF1, one of the key transcriptional effectors of the canonical Wnt pathway, is regulated by Wnt3a and *TCF4*, strengthens Wnt signaling during malignancy Notably, LEF1 can interact with *β‐catenin*, driving increased transcriptional activity even in the absence of changes to β‐catenin levels, and is implicated in increasing tumor colony outgrowth [356]. Moreover, studies have shown that *HOXB9* expression is induced by N‐acetylgalactosaminyltransferase (GalNAc‐T14). GalNAc‐T14 significantly contributes to enhancing the responsiveness of the *Wnt* pathway and to elevating the stabilization of β‐catenin. These molecular occurrences are probably linked to the development of an invasive characteristic in LUAD [87, 356, 358]. In addition, PITX2, as a TF, is also implicated in modulating the Wnt/β‐catenin signaling pathway. Within this context, PITX2 activates certain genes’ expression such as *cyclin D1* and *c‐Myc*. Studies have indicated *PITX2's* association with LUAD, suggesting that it functions as an upregulated oncogene in this cancer type. Findings also have been reported that *PITX2* is overexpressed in LUAD and correlates with poor patient prognosis. Furthermore, research demonstrates that *PITX2* enhances the transcription of Wnt3a, thereby promoting oncogenic effects through activation of the Wnt/β‐catenin pathway [359]. Wnt3a, a well‐known activator of this pathway, is implicated in various cancers due to its critical role in tumorigenesis. Its involvement in this pathway allows Wnt3a to influence tumor progression in a complex manner, either suppressing or promoting activity. Correspondingly, this dual role highlights the complexity of Wnt3a's function in cancer biology, suggesting that its effects may vary based on the specific cancer type. Studies have specifically shown that Wnt3a enhances the development and progression of solid tumors, particularly lung cancers. The mechanism by which Wnt3a exerts its effects involves promoting cancer cell proliferation and self‐renewal [359, 360].

As mentioned earlier, dysregulation of the Wnt signaling pathway is closely implicated in the tumorigenesis of lung cancer and other cancer types. The transcriptional landscape of *Wnt* genes is also markedly altered in LUAD and LUSC, revealing potential biomarkers for these malignancies [359]. Therefore, alterations in the expression levels of *Wnt* genes and their inhibitors, often driven by genetic mutations and epigenetic factors such as abnormal DNA methylation, can substantially contribute to cancer development. These alterations can lead to key oncogenic processes such as increased cell proliferation, and metastasis. Hence, DNA hypermethylation plays a crucial role in disrupting *Wnt* signaling pathways by suppressing the expression of essential regulatory genes. In lung cancer, studies have identified abnormal methylation patterns in multiple Wnt inhibitors, correlating with the aberrant activity of the Wnt signaling pathway. Notably, key Wnt inhibitors such as *APC*, wingless‐type protein 7a (Wnt7a), Wnt inhibitory factor‐1 (WIF1), and axis inhibition protein (AXIN) are frequently found to be hypermethylated in lung cancer, particularly NSCLC [347]. This epigenetic silencing leads to the disruption of normal Wnt signaling regulation, thereby promoting oncogenic processes [301]. Among Wnt inhibitors genes, two *homeobox* genes, *CDX2* and *EMX2*, have been identified as *Wnt* inhibitors. The hypermethylation of the promoters for *CDX2* and *EMX2* is associated with their downregulation, leading to transcriptional silencing in lung cancer [347, 361, 362, 363]. Both *CDX2* and *EMX2* are pivotal in regulating *Wnt* signaling pathways through their roles as inhibitors. Their promoter hypermethylation leads to decreased expression, facilitating enhanced Wnt signaling that promotes lung carcinogenesis. Understanding these mechanisms highlights potential therapeutic targets for restoring the function of Wnt inhibitors in lung cancer treatment strategies. Importantly, individual homeobox genes frequently converge on multiple oncogenic signaling cascades, with engagement patterns that are highly context‐dependent and shaped by tumor type, intrinsic genetic and epigenetic alterations, cell state, and the TME. For example, HOXA9 cooperates with cofactors such as PBX and MEIS to orchestrate a broad gene network and to interface with multiple signaling pathways, including Wnt, TGF‐β, PI3K/AKT, and NF‐κB, collectively regulating oncogenic processes such as EMT, autophagy, cell‐cycle progression, and cellular metabolism [281].

### 4.3 A Central Role of Homeobox Genes in Five Highest Incidence Cancer

As outlined earlier, dysregulation of homeobox genes is widespread across human malignancies, spanning solid tumors and hematologic cancers, and includes recurrent alterations in breast, colorectal, prostate, gastric, and notably lung cancers. These patterns provide a mechanistic basis for tumor‐type‐specific expression programs and clinical behavior. In particular, the dysregulation of HOX gene clusters has been reported in various solid tumors, with distinct expression patterns observed across different cancer types. For instance, genes in the HOXA cluster exhibit altered expression predominantly in breast and ovarian cancers. Additionally, genes in the HOXB cluster are commonly associated with colon cancer. Similarly, genes in the HOXC cluster are frequently upregulated in several malignancies, including colon and PCa. Moreover, genes in the HOXD cluster show aberrant expression in colon and breast cancers. Likewise, most genes across all four clusters of HOX have been reported to be dysregulated in lung cancer. Notably, recent studies suggest that *HOX* genes are also regulated at the level of nuclear‐cytoplasmic transport in carcinomas, adding another layer of complexity to their role in tumorigenesis. This suggests that the any dysregulation of *HOX* genes’ expression and localization may play an important role in the development and progression of various solid tumors. Furthermore, it appears that tumors arising from tissues with similar embryonic origins exhibit relatively similar patterns of *HOXA* and *HOXB* family gene expression. For example, tumors originating from endodermal tissues, such as the colon, prostate, and lung, show more comparable *HOXA* and *HOXB* genes expression profiles compared with breast tumors, which arise from the ectodermal mammary tissue [1, 3]. Importantly, *HOXA11‐AS* antisense RNA (*HOXA11‐AS*) has been found to be upregulated in NSCLC, suggesting its potential involvement in the development and progression of this cancer type. In contrast, the downregulation of the *PITX1* gene has been significantly associated with more advanced tumor stages across various cancer types. This highlights its potential role as a tumor suppressor and suggests its possible application as a prognostic marker [149, 364].

Beyond solid tumors, *HOX* genes are also involved in later developmental processes that establish cell identity, such as hematopoiesis. Expression of HOX gene clusters A, B, and C has been detected in HSCs, underscoring their critical regulatory roles in blood cell development [365]. Deregulation of specific *HOX* genes has been linked to leukemia, with aberrant expression patterns contributing to the pathogenesis of ALL and AML. This evidence highlights the important role HOX genes play in maintaining normal hematopoietic function and how their misexpression can drive malignancies in blood‐forming tissues [148]. Because homeobox dysregulation is widespread across lineages, it is informative to consider the five most common cancers as global exemplars, illustrating how *HOX* networks extend across solid tumors. According to GLOBOCAN 2022, lung cancer has the highest number of new cases and remains the leading cause of cancer death. The five cancers with the greatest global incidence in 2022 were lung (∼2.5 million cases; 12.4%), female breast (∼2.3 million; 11.6%), colorectal (∼1.9 million; 9.6%), prostate (∼1.5 million; 7.3%), and stomach (∼0.97 million; 4.9%). These figures provide the current worldwide benchmark for statements written for 2025 [366].

#### 4.3.1 Aberrant Expression of Homeobox Genes in Lung Cancer

In 2024, approximately 2,001,140 new cancer cases and 611,720 cancer‐related deaths were reported in the united states [367]. Lung cancer remains the first most common malignancy and the leading cause of cancer‐related mortality worldwide in 2024, resulting in the loss of about 350 lives each day [368, 369]. Lung cancer is classified into two main histological groups: NSCLC and small cell lung cancer (SCLC), which account for approximately 85% and 15% of all lung cancer cases, respectively. The NSCLC group can be further divided into various subtypes, with LUAD being the most common, representing around 40% of all lung cancer cases. The second most prevalent NSCLC subtype is LUSC, making up approximately 25% of lung cancer diagnoses. Additionally, large cell carcinoma with or without neuroendocrine features accounts for roughly 10% of lung cancer cases [370]. In lung cancer patients, tobacco use is significantly associated with 35% to % 50 of cases in men and around 17% in women. Additionally, the rising incidence of lung cancer cases can also be attributed to factors such as sedentary lifestyle, urban pollution, obesity, and increased use of cigarettes and alcohol. Moreover, the unregulated expression of the *HOX* genes in cancer can be influenced by factors such as temporospatial heterogeneity, gene dominance, genetic or epigenetic mechanisms, or a combination of these variables, as indicated by research studies [369].

As mentioned previously, homeobox genes are involved in the complex dynamics of cancer development, employing diverse mechanisms that contribute to the process of carcinogenesis. Their dual function as transcriptional regulators acting as both transcriptional activators and repressors is modulated by various factors, which renders their contribution to tumorigenesis multifaceted [3]. Therefore, any alterations in the expression of *homeobox* genes, whether through upregulation or downregulation, have been observed across different cancers, particularly in solid tumors such as lung cancer, especially in NSCLC. Importantly, NSCLC, which accounts for approximately 85% of all primary pulmonary carcinomas, remains the leading cause of cancer‐related mortality worldwide. Lung cancer is a complex biological process that arises from the intricate dysregulation of various oncogenes associated with cancer development. Despite advances in multimodal therapies including surgery, radiation, and chemotherapy, the 5‐year OS rate for NSCLC patients remains suboptimal. This underscores the critical need to identify precise and specific biomarkers that can enhance survival outcomes for NSCLC patients [371]. Research studies have demonstrated that numerous *HOX* genes, such as those from the HOXC and HOXD clusters, exhibit overexpression in primary NSCLC tissues and cell lines. For example, genes like *HOXC4* and *HOXC8* show significantly elevated levels in malignant cells compared with normal lung tissues. This dysregulation of *HOX* genes’ expression suggests that these TFs may contribute to the oncogenic processes involved in the development and progression of lung cancer [372]. In contrast, the downregulation of certain HOX gene clusters, such as *HOXC9* and *HOXD10*, has been linked to increased cell proliferation, while overexpression of *HOXA1* has also been observed in lung carcinoma. Furthermore, genes associated with four clusters of HOX gene exhibit distinct and complex expression patterns in lung cancer. Collectively, these alterations in *HOX* genes’ expression have been reported to negatively impact lung development, contributing to congenital abnormalities and the progression of lung carcinomas [1, 373].

In solid tumors, angiogenesis plays a significant role in the progression of them, recognized as an initial step in tumorigenesis. Moreover, it is a primary driving force behind tumor recurrence [3]. Research has reported the crucial role of specific *HOX* genes in angiogenesis associated with solid tumors. In particular, genes such as *HOXB7* and *HOXA11‐AS* have been discovered to actively promote angiogenesis in lung cancer [149, 374, 375]. In LUAD, *HOXB7* has been identified as a key factor involved in tumorigenicity and metastasis. Elevated levels of *HOXB7* expression are associated with poor clinical outcomes and reduced survival rates in patients. Notably, *HOXB7* is strongly linked to cell proliferation and metastasis in LUAD. Previous studies have shown that its knockdown leads to a reduction in the expression of certain genes such as *VEGFA* and *matrix metalloproteinase‐2* (*MMP‐2*) [376, 377]. In addition, aberrant expression of *HOXA11‐AS* has garnered attention for its potential involvement in the development and progression of NSCLC through the regulation of certain genes and various signaling pathways, such as the TGF‐β signaling pathway. A significant overexpression of *HOXA11‐AS* has been observed in NSCLC tissues, including LUAD and SCC. This implies that *HOXA11‐AS* likely plays a direct role in tumorigenesis, angiogenesis, and invasion in NSCLC [149, 374, 375].

##### 4.3.1.1 Homeobox Genes Dysregulation in Lung Cancer Pathogenesis

###### 4.3.1.1.1 *HOXA* Cluster's Role in Lung Cancer

*HOXA* cluster genes play an integral role in several types of cancer, including lung cancer, leukemia, oral SSC, CRC, and pancreatic cancer [378, 379]. Notably, there is an abundance of CGIs within the HOXA gene cluster, which are believed to facilitate transcription initiation. In normal tissues, these CGIs are generally nonmethylated, allowing for active transcription of the *HOXA* genes. However, aberrant methylation patterns within the HOXA cluster can lead to gene silencing, which is often observed in various cancers, including lung cancer. According to an earlier report, the *HOXA* loci in the human *LUAD* was methylated at several CpG islands [380], although the methylation of the HOXA cluster genes is predominantly found in NSCLC [381]. In lung primary SCC, frequent methylation patterns in HOXA‐associated CpG islands were identified in the first stage of tumors, particularly involving *HOXA1* and *HOXA7* [2].

*HOXA1* is known for its role in regulating various biological processes such as cell proliferation, differentiation, and apoptosis [190, 382]. The elevated levels of *HOXA1* expression has been consistently linked to various malignancies, including various types of lung cancer, where they play a significant role in tumorigenesis [383]. Accordingly, research has demonstrated that *HOXA1* expression is markedly elevated in NSCLC, suggesting its potential utility as a reliable biomarker for the diagnosis of this malignancy. Moreover, its expression correlates with advanced stages of the disease and higher stage tumor node metastasis (TNM) of in LUAD and LUSC. Additionally, its upregulation is correlated with advanced disease stages and higher TNM classifications in both LUAD and LUSC [384] [385]. Consequently, *HOXA1* appears to be intricately linked to various biological processes associated with lung cancer. Furthermore, functional analyses, incorporating gene ontology and Kyoto encyclopedia of genes and genomes (KEGG) pathway assessments, indicating that *HOXA1* plays a pivotal role in essential biological processes such as centromeres’ DNA binding, and demonstrates a significant linkage to the P53 signaling pathway. This connection suggests that *HOXA1* may influence the progression of NSCLC by regulating pathways critical for cell cycle control and programmed cell death [385]. However, a previous study has reported that hypermethylation of the *HOXA1* CpG island is present in LUAD. Subsequent studies have revealed that *HOXA1* expression is detectable in 46% of SCLC patient samples. Notably, reduced *HOXA1* expression is strongly associated with poorer prognosis and decreased survival rates in SCLC patients. Building on this, research has shown that HOTAIR regulates *HOXA1* expression through methylation. These findings underscore the close relationship between *HOTAIR* and *HOXA1* methylation [382, 384, 386]. Therefore, research indicates that depletion of *HOTAIR* leads to a significant decrease in the expression of the DNA methyltransferase enzymes *DNMT1* and *DNMT3b*, which in turn correlates with decreased methylation of the *HOXA1* gene [382]. This suggests a direct regulatory interaction between the HOTAIR and the *HOXA1* gene, where in *HOTAIR* modulates the methylation status of *HOXA1* through its influence on *DNMT* expression [387]. The mechanism by which *HOTAIR* influences *HOXA1* further involves its interaction with epigenetic regulators, particularly the *polycomb repressive complex 2* (*PRC2*). *HOTAIR* has been shown to recruit PRC2 to target genes, facilitating the trimethylation of histone H3 at lysine 27 (H3K27me3), a mark associated with gene silencing. This process is crucial for maintaining the transcriptional repression of genes like *HOXA1*, thereby influencing cellular processes such as proliferation and invasiveness in cancer cells, while the mechanism is unclear [388, 389]. As mention earlier, *HOTAIR* significantly influences the expression of *HOXA1*, demonstrating a multifaceted regulatory role in its downstream target gene. This interaction is intricately linked to the regulation of the NF‐κB signaling pathway, which plays a crucial role in cancer progression and drug resistance. The NF‐κB pathway plays a crucial role in various cellular processes, such as cell survival, and proliferation, as well as innate and adaptive immune response. However, when this pathway is abnormally activated, it is strongly associated with the transcription of genes that promote tumorigenesis, contributing to the process such as enhanced cancer cell proliferation, inhibition of apoptosis, and the enhancement of angiogenesis in various types of cancer [390]. In addition to *HOTAIR* role in lung cancer, *HOTAIR* has also been shown to significantly influence the regulation of the NF‐κB pathway across various cancer types. This underscores the critical nature of *HOTAIR*’s interaction with this pathway, demonstrating its potential as a key regulatory element in tumor biology. In vitro studies on SCLC cell lines have shown that silencing *HOTAIR* leads to inhibition of the NF‐κB pathway, while the overexpression of *HOXA1* also diminishes this pathway's activity. In contrast, reducing HOXA1 expression reverses these effects, resulting in the activation of the NF‐κB pathway. This implies a dynamic regulatory relationship between *HOTAIR*, *HOXA1*, and in the context of SCLC. Besides, the NF‐κB pathway is significantly activated in multidrug‐resistant SCLC cell lines compared with their parental counterparts, leading to a reduced sensitivity of these cells to chemotherapy drugs. Consequently, research suggests that inhibiting this pathway with specific agents has been shown to improve the responsiveness of these resistant cells to various chemotherapeutic treatments. Notably, blocking NF‐κB not only facilitates apoptosis but also induces cell cycle arrest. Therefore, NF‐κB inhibitors could represent a potential therapeutic strategy for overcoming drug resistance in patients with elevated *HOTAIR* expression [382, 386, 387]. Another key factor involved in the regulation of *HOXA1* expression is the influence of *miRNAs* in lung cancer. Several *miRNAs* exhibit a significant relationship with *HOXA1* expression, emphasizing their regulatory roles [320]. Specifically, *miR‐100, miR‐181b‐5p, miR‐181d‐5p*, and *miR‑577* have been identified as key regulators of *HOXA1* expression in various types of lung cancer [385, 391, 392]. For instance, *miR‐100* has been implicated in regulating *HOXA1* expression in both SCLC and NSCLC, highlighting its complex role in lung cancer progression [392, 393]. In SCLC, *HOXA1* expression is inversely correlated with *miR‐100* levels, as *miR‐100* directly targets the 3′‐UTR of the *HOXA1* gene, leading to reduced *HOXA1* expression. In vitro studies on multidrug‐resistant SCLC cell lines have demonstrated that restoring *HOXA1* expression enhances sensitivity to chemotherapy agents. Conversely, silencing *HOXA1* further exacerbates drug resistance in these cells. Additionally, elevated *miR‐100* levels in drug‐resistant cell lines are inversely correlated with *HOXA1* expression, underscoring the regulatory role of *miR‐100* in chemoresistance [392]. In NSCLC, studies have revealed another aspect of miR‐100's influence on *HOXA1* expression, underscoring its antitumor role. Notably, *miR‐100* is frequently downregulated, leading to the upregulation of *HOXA1* expression, which is associated with aggressive clinical features such as advanced TNM stage, lymph node metastasis, and poor OS. Functional studies confirm that restoring miR‐100 expression suppresses NSCLC cell proliferation, invasion, and migration by downregulating the expression of *HOXA1* alongside with key pathways, including EMT process and Wnt/β‐catenin signaling pathway. Moreover, *miR‐100* overexpression leads to decreased levels of key Wnt/β‐catenin components, such as cyclin D1 and c‐Myc, further limiting tumor progression. These findings underscore the pivotal role of *miR‐100* in suppressing the invasive and aggressive properties of NSCLC cells [393]. Another miRNA that targets *HOXA1* expression in NSCLC is *miR‐577*. Functionally, *miR‐577* directly binds to the 3′‐UTR of *HOXA1*. The expression of *miR‐577* has been found to be significantly reduced in NSCLC tissue samples and related cell lines compared with their nontumorous counterparts. In contrast, *HOXA1* was notably upregulated at both the mRNA and protein levels in NSCLC, with its overexpression inversely correlated with *miR‐577* expression, emphasizing its oncogenic role in the tumorigenesis of NSCLC. Experimental analyses also revealed that restoring *miR*‐*577* expression led to a downregulation of *HOXA1* and suppressed both the proliferation and invasion of NSCLC cells, suggesting its potential tumor‐suppressive role. These findings collectively imply that *miR‐577* acts as a tumor suppressor in NSCLC by downregulating *HOXA1* [320]. In LUSC, *miR‐181b‐5p* and *miR‐181d‐5p* have explored to significantly reduce compared with normal lung tissues, showing an inverse relationship with increased *HOXA1* expression. This interaction is believed to influence several signaling pathways associated with lung cancer progression, particularly P53 pathway [385].

Expanding on the involvement of other genes within the HOXA cluster, *HOXA2* has been identified as another member whose expression is altered through hypermethylation in both SCC and NSCLC. Evidence suggests that aberrant *HOXA2* methylation patterns could serve as a potential biomarker for lung cancer, as these patterns stratify lung SCC into distinct transcriptional phenotypes, which are associated with varying prognoses [303, 394]. Previous research suggested that *HOXA2* is more likely to be methylated in patients with recurrent SCC compared with those without recurrence, although no significant link was found between them [394]. The methylation profiles of *HOXA2*, frequently hypermethylated in SCC and linked to its prognosis, can provide prognostic information for SCC patients and also may assist in identifying biomarkers associated with the prognosis of NSCLC patients [303, 394, 395]. In NSCLC, *HOXA2* is often downregulated and acts as a tumor suppressor, contributing to lung cancer progression. Additionally, several factors influence *HOXA2* expression in NSCLC, including *LINC00472* (an intergenic lncRNA) and miR‐1275. In NSCLC, both *LINC00472* and *HOXA2* expressions are significantly reduced, while *miR‐1275* levels are elevated. The increased expression of *miR‐1275* is likely associated with a malignant cell phenotype in NSCLC, as it targets *HOXA2* expression. Experimental studies have shown that the re‐expression of *LINC00472* can directly interact with miR‐1275, inhibiting its expression, which subsequently restores *HOXA2* levels and mitigates the *miR‐1275*‐related malignant phenotype. Consequently, restoring *HOXA2* and overexpressing *LINC00472* lead to a reduction in malignant behaviors such as proliferation and EMT, while promoting apoptosis; however, these effects can be reversed by elevated *miR‐1275* levels or silencing of *HOXA2*. The interplay between the *LINC00472*/miR‐1275/HOXA2 axis is crucial in the progression of NSCLC and accentuates potential therapeutic targets for intervention [395].

Another member of the HOXA cluster with a recognized role in various cancers is *HOXA3*. Extensive studies have reported its potential involvement in cancers such as colon cancer and nasopharyngeal carcinoma. Interestingly, the expression of *HOXA3* shows cancer‐specific variation. For instance, *HOXA3* expression is elevated in colon cancer, which is associated with poor survival rates. In contrast, *HOXA3* expression is reduced in lung cancer, particularly NSCLC, due to elevated methylation levels. Notably, the methylation levels of *HOXA3* are significantly elevated in LUAD samples, suggesting lower *HOXA3* expression may be an independent protective factor. These findings suggest that lower *HOXA3* expression, driven by hypermethylation, may act as an independent protective factor in LUAD, correlating with improved prognostic outcomes [378, 396]. In vitro studies on NSCLC cell lines indicate that *HOXA3* is downregulated, which correlates with enhanced chemotherapy resistance and directly influences EMT‐related markers, such as a decrease in E‐cadherin expression. Research has also demonstrated that, both HOXA3 mRNA and its protein can interact with the lncRNA *HOXA‐AS3*, located on chromosome 7p15.2, suggesting that *HOXA‐AS3* may contribute to the downregulation of *HOXA3* in NSCLC. Experimental evidence further indicates that *HOXA‐AS3* is upregulated in response to drug resistance, including cisplatin treatment in NSCLC. Notably, this elevated expression has been observed *both* in vivo and in vitro. Moreover, silencing *HOXA‐AS3* enhances cisplatin efficacy by upregulating *HOXA3* expression, which in turn promotes cell apoptosis. Furthermore, silencing *HOXA‐AS3* improved the efficacy of cisplatin and upregulated *HOXA3* expression, which subsequently enhanced cell apoptosis [273].

HOXA5 is a critical TF involved in both normal lung development and the progression of lung cancer, particularly in NSCLC. Its dual role indicates its importance in both physiological and pathological contexts [397]. HOXA5 plays a critical role in the proper development of the lung. Research on mouse models has shown that a lack of *HOXA5* leads to severe respiratory distress at birth, due to significant structural defects in the lung, including impaired innervation of the diaphragm and abnormal tracheal formation. This underscores the pivotal role of HOXA5 in regulating the development of lung epithelial cells and associated structures, such as alveoli and airway cell. Specifically, HOXA5 influences the differentiation of various lung cell types and is involved in the signaling pathways that govern lung morphogenesis [398]. HOXA5 also plays a critical role in regulating cell proliferation through its interaction with the CDK inhibitor p21. This interaction is essential for controlling the cell cycle and promoting apoptosis in cancer cells. Additionally, it inhibits tumor growth, highlighting the tumor‐suppressive function of *HOXA5* [399]. Research has established a direct link between the downregulation of *HOXA5* expression and increased cell proliferation and migration in lung cancer, particularly in NSCLC samples. Its expression is significantly correlated with clinicopathological features, including tumor size and lymph node metastasis. These findings indicate the critical role of *HOXA5* as a tumor suppressor, with its reduced expression strongly associated with lung tumorigenesis. So far, the lower level of *HOXA5* expression is correspondingly linked to various adverse clinicopathological features, emphasizing its importance in cancer progression. Experimental evidence supports this notion, showing that ectopic *HOXA5* overexpression in invasive lung cancer cell lines suppresses cell migration and invasion. Functionally, *HOXA5* also suppresses cancer invasiveness partly by regulating cytoskeletal remodeling. Emerging research has revealed a correlation between the downregulation of *HOXA5* expression and miRNA regulation, specifically, *miR‐196a*. Therefore, *HOXA5* expression is influenced by *miR‐196a*, a miRNA that directly binds to its 3′‐UTR regions, leading to reduced *HOXA5* levels at both mRNA and protein levels. This interaction promotes NSCLC cell proliferation, migration, and invasion, creating an inverse relationship between miR‐196a and *HOXA5* expression in NSCLC tissues. While miR‐196a has been detected to implicate in multiple types of cancers, its complete role in lung cancer remains under investigation. However, these findings suggest that targeting miR‐196a could be a promising therapeutic strategy to restore *HOXA5* expression and inhibit tumor progression [400, 401]. The abnormal methylation of *HOXA5* is another identified epigenetic factor that downregulates its expression in lung cancer, a key epigenetic mechanism contributing to lung cancer tumorigenesis. In lung cancer, the expression of *HOXA5* has detected to often absent due to the abnormal methylation of its promoter region, particularly in NSCLC's samples. This methylation‐induced silencing of the *HOXA5* gene contributes to the development and progression of NSCLC's samples, and it can consider as a significant factor in the pathogenesis of this lung cancer subtype. Remarkably, patients with Stage I lung cancer are more likely to have methylated *HOXA5* than never‐smokers, with methylated *HOXA5* being detected in an average of 81.3% of NSCLCs and 51.8% of nonmalignant lung tissues [301, 397, 399, 402]. In NSCLCs, the level of *HOXA5* expression also has been reported to dramatically reduce due to promoter hypermethylation. Consequently, the epigenetic downregulation of *HOXA5* in NSCLC is associated with several clinically relevant factors, including increased tumor size, metastasis to both the primary tumor and lymph nodes, as well as higher TNM staging. Furthermore, the suppression of *HOXA5* appears to be linked with the promotion of LUAD, suggesting that *HOXA5* functions as a tumor suppressor in NSCLC. This downregulation of the *HOXA5* gene also likely contributes to the progression and development of the cancer, particularly LUAD [394, 399].

*HOXA7* has been found to exhibit promoter hypermethylation in lung cancer, with a higher incidence in NSCLC, especially the LUAD subtype. Numerous studies have demonstrated that *HOXA7* promoter hypermethylation is significantly more detectable in both plasma and tumor samples of lung cancer patients compared with benign or healthy controls, underscoring its potential as a reliable diagnostic biomarker for lung cancer. For instance, *HOXA7* hypermethylation was highly detectable in plasma samples from Stage IV patients. Notably, elevated *HOXA7* methylation levels are associated with advanced TNM stages, particularly stage IV disease, further emphasizing its potential as a prognostic biomarker [403, 404]. In addition, studies on the methylation profiles of certain genes in plasma samples suggest that combining the detection of *HOXA7* promoter hypermethylation with the promoter methylation of other genes, such as *cysteine dioxygenase type 1* (*CDO1*), *tachykinin precursor 1* (*TAC1*), and *SRY‐box TF 17* (*SOX17*), demonstrates promising diagnostic performance for early‐stage NSCLC, achieving sensitivity and specificity values of up to 90% and 71%, respectively [403]. Beyond its epigenetic regulation, the reduction of *HOXA7* expression in lung cancer is further influenced by its involvement in a ceRNA network, particularly with *miR‐17‐5p* and *lncRNA* histocompatibility leukocyte antigen complex P5 (HCP5). Studies have highlighted the role of the *HCP5/miR‐17‐5p/HOXA7* axis in LUAD progression, with implications for ferroptosis a type of regulated cell death metastasis, and EMT. In LUAD samples, *lncRNA HCP5* is significantly upregulated and strongly associated with EMT, promoting tumor growth and metastasis. In vitro studies using LUAD cell lines have demonstrated that HCP5 overexpression increases *HOXA7* levels by binding to and suppressing *miR‐17‐5p*. This upregulation of *HOXA7* by *HCP5* promotes ferroptosis. Conversely, silencing *HCP5* increases *miR‐17‐5p* levels, leading to the inhibition of *HOXA7* expression and suppression of ferroptosis. Additionally, *HOXA7* overexpression has been shown to significantly enhance cellular growth, proliferation, and migration in LUAD cells. Overall, this regulatory axis underscores the oncogenic role of *HCP5/miR‐17‐5p/HOXA7* in LUAD and highlights its potential as a promising therapeutic target to inhibit tumor progression and metastasis [405].

HOXA9 functions as a key transcriptional regulator, binding to DNA and modulating the expression of numerous genes. This activity is essential during embryonic development and in preserving the characteristics of HSCs. The protein collaborates with other TFs, enhancing its specificity in binding to target genes, many of which are involved in cell division and cell survival processes [283, 406]. HOXA9 has been reported to involve in the pathogenesis of various cancers, particularly AML and solid tumors such as lung cancer [407, 408]. Methylation of the *HOXA9* promoter region is a crucial mechanism by which its expression is repressed in cancer. In lung cancer, for instance, excessive methylation of the *HOXA9* promoter results in diminished transcriptional activity, thereby contributing to tumorigenesis. This epigenetic alteration disrupts the normal regulatory pathways, enabling cancer cells to avoid differentiation and maintain uncontrolled proliferation [283, 381]. As noted earlier, the NF‐κB signaling pathway is involved in the regulation of various cellular processes, including inflammation, proliferation, and apoptosis. Therefore, deregulation of this pathway is implicated in the development and progression of multiple types of cancer, such as lung, gastric, and hepatocellular carcinoma. Remarkably, HOXA9 as a TF has been found to be associated with the NF‐κB signaling pathway [283]. *HOXA9* has been reported to promote apoptosis and inhibit autophagy by transcriptionally regulating the *p65* subunit of NF‐κB (RELA) in cutaneous SCC. Further studies have shown that the responsive region of *NF‐κB* is located within the first 400 base pairs of the *HOXA9* promoter. In NSCLC, *HOXA9* expression has detected to downregulate in tumor tissues compared with matched nontumor tissues, and overexpression of *HOXA9* inhibited cell migration and invasion, which is linked to the regulation of NF‐κB activity. However, there are conflicting reports regarding the effects of *HOXA9* on the activation of the NF‐κB signaling pathway. This could be attributed to the potential existence of cell heterogeneity, and further in‐depth investigations are warranted to elucidate the precise mechanisms by which *HOXA9* regulates the NF‐κB signaling pathway [283]. The frequent hypermethylation of the *HOXA9* gene plays a significant role in ovarian carcinogenesis, particularly in the early stages (FIGO stage I‐II) [408]. In breast cancer, *HOXA9* and *HOXA10* function as tumor suppressor genes, and methylation at their promoter CpGs islands has been shown to provide valuable prognostic information in breast cancer through interactions at the *HOXA10–HOXA9* promoter CpG sites [407, 409]. A *HOXA9* promoter hypermethylation was detected in 42.6% of NSCLC tumors (23 out of 54) [410]. Previous studies on NSCLC have observed that *HOXA9* methylation rates increase with disease progression and cancer stages, with the rate of methylation increasing from Stage I (38.5%) to Stages III–IV (66.7%). However, no significant correlation was found with the TNM stage, and low detection rates were observed with the methylation frequency [410]. The study results indicated that among Stage IA LUAD samples, 42.9% of the *HOXA9* gene was methylated, which is consistent with the primary tumor DNA. Additionally, a prior study also found hypermethylated *HOXA9* in 80% of lung cancers during their early stages [381, 411]. Furthermore, *HOXA9* hypermethylation in its promoter region has been detected in six lung cancer cell lines, whereas it is not observed in normal epithelial cells. Poor recurrence‐free survival (RFS) was observed in *HOXA9* hypermethylation in never‐smokers, appearing that *HOXA9* hypermethylation can serve as an independent prognostic factor for RFS in patients with nonsmoking NSCLC [412]. Functionally, the HOXA9 protein appears to prevent lung cancer cell migration, whereas its downregulation enhances cell invasiveness and promotes migration [406, 412]. Accordingly, *HOXA9* overexpression in NSCLC cell lines has been shown to prevents invasion [410]. Moreover, *HOXA9* methylation is highly prevalent in SCC compared with adenocarcinoma in lung cancer [413], and dysregulated *HOXA9* is implicated in a variety of solid tumors, particularly in lung cancer [406, 412]. Additionally, *HOXA9* promoter methylation can be considered a potential early lung cancer diagnostic biomarker and a tool for prognosis prediction. However, a deeper understanding of the mechanisms underlying the relationship between HOXA9 and the signaling pathways is still needed such as NF‐κB [381, 411].

Based on a cohort study, *HOXA9* gene methylation along with two other methylated genes (*CDO1* and *TAC1*) were considered as valuable biomarkers for diagnostic tests for NSCLC due to their sensitivity and specificity. Therefore, characterized methylation biomarkers might be useful for accurately detecting the early diagnosis and stages of NSCLC [414]. Subsequent experiments have revealed that hypermethylated *HOXA9* has been detected in bronchial lavage fluids, indicating its potential as a biomarker in lung cancer [406]. *HOXA2* and *HOXA9* hypermethylation have been associated with high‐methylation and low‐methylation epigenotypes in NSCLC [415]. Within the *HOXA* clusters, both *HOXA7* and *HOXA9* promoters contain a high proportion of methylated DNA in the initial stage of SCCs, with *HOXA9* being prominently methylated in 80% of the Stage I tumors, suggesting its potential as a promising marker for lung cancer [2]. In primary lung carcinomas, particularly LUSC, the CpG islands of *HOXA7* and *HOXA9* are methylated in tumors by 45 and 68%, respectively, despite being unmethylated in normal lung tissue [2]. These epigenetic modifications, which result in reduced expression of *HOXA7* and *HOXA9*, may contribute to tumor recurrence and could be useful for detecting NSCLC [2, 303, 412]. In sputum and plasma, a high level of diagnostic accuracy can be identified for early‐stage lung cancer by detecting hypermethylated promoters of *HOXA7* and *HOXA9* [406, 416]. Furthermore, since *HOXA9* exhibits higher methylation levels in SCLC compared with NSCLC, both genes may serve as valid biomarkers for SCLC. Data from lung cancer liquid biopsies support that *HOXA9* methylation levels in circulating cell‐free DNA (cfDNA) can be effectively assessed in lung cancer subtypes, demonstrating *HOXA9’*s high sensitivity (63.8%) [303, 413]. In LUAD, current evidence suggests that *HOXA9* promoter methylation serves as a potential biomarker for early‐stage diagnosis and risk stratification. Studies on Brazilian patients have shown that lower *HOXA9* methylation levels are associated with improved cancer‐specific survival (CSS); however, this marker was not identified as an independent prognostic factor in this study [417].

*HOXA10* expression in lung cancer has been reported inconsistently across various studies, with conflicting findings regarding its role. *HOXA10* is also referred to by several alternative names, including *PL*, *HOX1*, *HOX1H*, and *HOX1.8*. Studies have reported methylation of *HOXA10* in different lung cancer subtypes, such SCC, NSCLC, and LUSC [303, 394, 418]. These findings suggest that the methylation profiles of *HOXA10* may provide valuable prognostic information for patients with SCC and NSCLC [394]. Notably, *HOXA10* methylation has been highly detected in invasive peripheral pulmonary adenocarcinoma (ADC) compared with atypical adenomatous hyperplasia (AAH) or adenocarcinoma in situ (AIS). This indicates that *HOXA10* methylation could be involved in tumor progression in the ADC. However, other studies have reported increased *HOXA10* expression in lung cancer, highlighting the complexity and potential dual role of *HOXA10* in tumor development [418, 419], *HOXA10* overexpression is probably involved in the tumorigenesis and progression of NSCLC, particularly impacting LUSC more than LUAD. Analysis of extensive datasets indicates that *HOXA10* mRNA expression is significantly elevated in NSCLC, with levels markedly higher in LUAD and even more upregulated in LUSC compared with noncancerous tissues—highlighting its importance in LUSC. Accordingly, studies have demonstrated that *HOXA10* plays a significant role in the tumorigenesis and progression of NSCLC by influencing various signaling pathways. For instance, HOXA10 overexpression in NSCLC has been reported to associate with dysregulation of the Wnt and FGF pathways, both critical for tumor progression. Elevated *HOXA10* levels are associated with increased *FGF10* and *FGF17* are correlated with the marked upregulation of *HOXA10* in NSCLC, further supporting its role in tumor progression [418, 420].

Furthermore, the regulation of *HOXA10* expression in lung cancer is influenced by *lncRNAs* and *miRNAs*. Research has identified several *lncRNAs* that play crucial roles in regulating HOXA10 expression, including *HOXA11‐AS* (also known as *ENSG00000240990*), *LINC00461*, *LINC00466*, and *LINC00483*. Accordingly, *miRNAs* also play a crucial role in modulating the level of *HOXA10* expression, including *miR‐144*, *miR‐195*, and *miR‐588*, further emphasizing the complexity of its regulation and its importance in NSCLC pathogenesis [421, 422, 423]. A computational study on a ceRNA network has demonstrated that lncRNA *HOXA11‐AS* regulates the expression of *HOXA10*. The interaction between the *HOXA11‐AS–HOXA10* pair and four *miRNAs*, specifically *hsa‐let‐7a/b/f/g‐5p*, is significantly associated with survival outcomes in lung cancer patients [421, 424]. Notably, lncRNA *HOXA11‐AS* can function either as a tumor suppressor or promoter depending on the cell type, and its expression has been explored to be aberrantly altered in various cancers. Therefore, its involvement in complex molecular networks highlights its critical role in tumorigenesis and cancer progression [423, 425]. For instance, lncRNA *HOXA11‐AS* is significantly upregulated in LUAD, alongside the overexpression of *HOXA10*. This suggests that *HOXA11‐AS* plays a crucial role in the progression of NSCLC, potentially through its influence on target genes, particularly *HOXA10*. Further research is essential to elucidate the precise mechanisms by which *HOXA11‐* *AS* influences different malignancies, and emerging studies suggest that it may serve as a valuable biomarker for lung cancer prognosis [418, 421, 424]. *LINC00466* and *LINC00483* also have detected to play crucial roles in LUAD progression by regulating *HOXA10* expression through interactions with their specific related *miRNAs*, particularly *miR‐144*. It should be noted that in LUAD, *LINC00483* and *LINC00466* are significantly upregulated, while *miR‐144* is downregulated, resulting in the enhanced expression of *HOXA10* [423, 426]. Studies on LUAD tissues have also demonstrated that elevated expression of *LINC00466* and *HOXA10*, combined with reduced *miR‐144* expression, is strongly associated with cancer progression. Notably, silencing *LINC00466* or increasing *miR‐144* expression leads to a significant reduction in HOXA10 levels. Silencing *LINC00466* reduces its binding to *miR‐144*, thereby increasing *miR‐144* levels and effectively suppressing processes that related to cancer development such as cell proliferation and migration, while promoting apoptosis. These findings collectively underscore the critical role of the *LINC00466–miR‐144–HOXA10* axis in driving LUAD progression and suggest promising therapeutic targets within this pathway [426]. Similarly, *LINC00483* binds competitively with miR‐144, effectively impacting the enhancement of HOXA10 in LUAD. This lncRNA contributes to tumor progression by promoting EMT, cell invasion, migration, and reducing radiosensitivity. Therefore, investigation had indicated that knockdown of *LINC00483* reduces its binding to miR‐144, thereby increasing *miR‐144* levels, which suppress *HOXA10* expression. This suppression is directly associated with reduced EMT, cell proliferation, and invasion and increased radiosensitivity. Additionally, silencing *LINC00483* decreases the expression of *MMP‐2* and *MMP‐9* (mesenchymal markers) while increasing the expression of E‐cadherin (an epithelial marker). These findings highlight the oncogenic role of *LINC00483* and its potential as a therapeutic target in LUAD [423, 426]. In addition, the *LINC00461/miR‐195/HOXA10* axis has also been implicated in the progression of LUAD. Overexpression of *LINC00461* suppresses *miR‐195*, leading to increase the levels of *HOXA10* expression. Additionally, upregulation of *LINC00461* is associated with elevated the level of expression of critical genes, including *MMP‐2*, *MMP‐9*, and BCL‐2, while simultaneously reducing the expression of the BAX proapoptotic protein. As a result of upregulation of *LINC00461*, this axis promotes cell proliferation, migration, and reducing radiosensitivity in LUAD. Conversely, silencing *LINC00461* exerts suppressive effects on LUAD by modulating the interaction between *miR‐195* and *HOXA10*, highlighting its potential as a therapeutic target for LUAD [427]. Circular RNAs (circRNAs), a stable subclass of ncRNAs, have emerged as critical regulators in cancer biology, often functioning as *miRNAs* sponges. Under normal physiological conditions, circRNAs participate in various biological processes such as gene regulation. Although their role in lung cancer remains relatively underexplored, growing evidence suggests that circRNAs contribute significantly to cancer progression and malignancy, including in lung cancers such as NSCLC and LUAD. Recent studies have identified circRNAs, particularly *circCSNK1G3* and *circ_0010235*, as key players in regulating *HOXA10* expression [422]. In primary LUAD tissues and related cell lines, *circCSNK1G3* has been shown to be aberrantly expressed, promoting critical processes such as cell proliferation and migration by sponging *miR‐143‐3p*. This interaction suppresses *miR‐143‐3p* expression, leading to *HOXA10* upregulation and facilitating tumor growth and metastasis [422]. Similarly, *circ_0010235* is overexpressed in NSCLC, where it regulates *HOXA10* by sponging *miR‐588*, thereby driving malignancy and reducing radiosensitivity. Overexpression of *circ_0010235* facilitates tumor progression by increasing *HOXA10* levels, while its downregulation, coupled with miR‐588 upregulation, suppresses malignancy and enhances radiosensitivity in NSCLC. These findings underscore the potential therapeutic value of targeting the *circCSNK1G3/miR‐143‐3p/HOXA10* and *circ_0010235/miR‐588/HOXA10* axes in lung cancer [422, 428].

*HOXA11* as an important TF plays a critical role in the proliferation, differentiation, and embryonic development of various tissues, particularly the endometrium [190, 283]. A prior study has indicated that the methylation of the promoter regions of *HOXA5* and *HOXA11* leads to transcriptional silencing, resulting in the loss of their tumor suppressor activities [381]. Evidence suggests that the *HOXA11* gene is hypermethylated at CpG sites in adenocarcinomas compared with normal lung tissue; however, later studies revealed that *HOXA11* was hypermethylated in SCC (74%) and adenocarcinoma (63%), indicating a higher prevalence in SCC [429, 430]. There are instances when LUADs originate from preneoplastic AAH lesions, which progress into AIS and eventually lead to invasive lung cancer. Hypermethylation of *HOXA1* and *HOXA11* at CGIs has been detected in AIS, suggesting that it might be possible to create specific biomarkers for the stages of development of LUAD based on the methylation level of these genes in lesions [431]. *HOXA11* has been found hypermethylated in six lung cancer cell lines, including 69% of primary NSCLCs. *HOXA11* hypermethylation is accompanied by downregulation. It is possible that hypermethylation of *HOXA11* at its promoter in NSCLC drives progression by promoting cell proliferation and migration. In LUAD patients, *HOXA11* hypermethylation may be used as a diagnostic and prognostic marker [429, 432].

###### 4.3.1.1.2 Even‐Skipped Homeobox Genes’ Role in Lung Cancer

Previous studies have shown that hypermethylation of *EVX1* is epigenetically associated with PCa [26]. Similarly, in NSCLC, particularly LUAD, *EVX1* promoter promoter hypermethylation at CpG sites has been reported to correlate with lack of gene expression in [394, 433]. However, within some normal lung tissues have also detected to be methylated with *EVX1* [434]. Additionally, it has been indicated that methylated *EVX1* causes downregulation in lung primary tumors [394]. According to the study, a significant relationship has been identified between clinicopathological parameters and *EVX1* hypermethylation. Furthermore, hypermethylated *EVX1* may be associated with its silencing and determination of precancerous stages that influence tumor aggressiveness [433]. It was reported that the *even‐skipped homeobox 2* (*EVX2*) gene has been detected hypermethylated at CGIs in SCCs’ samples [434, 435]. Moreover, methylated EVX2 was more prevalent in SCCs than in adenocarcinomas, whereas methylated Ras‐association domain family member 1A (RASSF1A) was more frequently observed in adenocarcinomas compared with SCCs in clinical samples from NSCLC patients. A high level of *EVX2* promoter methylation has also been observed in NSCLC compared with noncancerous lung tissues [436]. In NSCLC, methylated *EVX2* due to epigenetic alteration might be caused by the over‐activation of the *PI3K/Akt* pathway [434].

###### 4.3.1.1.3 HOXB Cluster's Role in Lung Cancer

The expression levels of HOXB cluster genes exhibit notable variation across different cancer types, including lung cancer, and are regulated by a complex interplay of factors. Among these, *HOXB5* plays a pivotal role in the Wnt signaling pathway, as previously described [3, 18]. Therefore, alterations in the expression of *HOXB5* in lung cancer have been shown to disrupt this essential pathway, thereby implicating in the tumor progression and malignancy. Similarly, *HOXB9* emerges as a significant player in cancer biology, particularly during lung cancer metastasis, where it acts as a target gene in the Wnt/TCF signaling pathway. Furthermore, epigenetic mechanisms, such as aberrant methylation of specific *HOXB* cluster genes, have been identified as contributors to dysregulation of genes within HOXB cluster, ultimately influencing the onset and progression of various types of cancer, including lung cancer [3, 18, 356]. For instance, hypermethylation of genes in the HOXB cluster, including *HOXB2*, *HOXB3*, *HOXB4*, *HOXB9*, and *HOXB1*, has been reported in several types of cancers. Specifically, *HOXB3* hypermethylation has been linked to epithelial ovarian cancer [303]. In lung cancer, particularly LUAD, hypermethylation of *HOXB3* and *HOXB4* has been identified, suggesting their potential as diagnostic biomarkers [303, 396, 437]. Consequently, alterations in the expression of some genes within the HOXB cluster have been observed in lung cancer studies, as discussed in greater detail below [438].

HOXB2, similar to other HOX proteins, acts as a TF that governs gene expression critical for cellular processes such as differentiation and development. Dysregulation of *HOXB2* expression has been linked to tumorigenesis and the progression of lung cancer, including NSCLC. This gene is regulated by miR‐139‐5p, a miRNA with potent tumor‐suppressive effects. By selectively binding to the 3′‐UTR region of *HOXB2*, miR‐139‐5p downregulates its expression, thereby inhibiting tumor growth through enhanced apoptosis and reduced cellular proliferation. Experimental studies on the lung cancer cell lines have demonstrated that the interaction between miR‐139‐5p and *HOXB2* plays a pivotal role in modulating sensitivity to chemotherapy drugs, including cisplatin. Moreover, upregulating *miR‐139‐5p* has been shown to reverse resistance to chemotherapeutic agents such as cisplatin and paclitaxel in these cell lines. This effect is mediated through the suppression of *HOXB2* and concurrent modulation of key signaling pathways, particularly the PI3K/AKT pathway and the activation of caspase‐3. The PI3K/AKT pathway, known for regulating cell survival and proliferation, is downregulated following the overexpression of *miR‐139‐5p*. Simultaneously, this upregulation increases the expression of *caspase‐3*, a critical enzyme involved in apoptosis. Together, these changes not only suppress tumor cell proliferation but also enhance apoptosis, providing a potential therapeutic approach to overcome drug resistance in lung cancer. Thus, targeting the miR‐139‐5p/*HOXB2* axis holds promise for improving the effectiveness of chemotherapy and reducing tumor progression in lung cancer patients [439, 440].

The involvement of the *HOXB3* gene in the prenatal lung during developmental stages has been linked to cellular differentiation processes. For instance, studies have demonstrated that HOXB3 promotes the expression of clara cell marker genes, facilitating the differentiation of M3E3/C3 cells into clara‐like cells, thereby underscoring its importance in normal lung development. However, the precise mechanisms underlying its function during these stages remain unclear [441]. Beyond its developmental role, HOXB3 has emerged as a significant factor in cancer biology, particularly in lung cancer. While aberrant methylation of *HOXB3* has been observed, other studies have identified elevated *HOXB3* expression levels in various malignancies, with a notable emphasis on lung cancer [303, 396, 437]. In lung cancer, particularly LUAD, *HOXB3* and *HOXB4* are epigenetically implicated through abnormal methylation patterns. Methylation analysis revealed that the promoter regions of the *HOXB3* and *HOXB4* genes are highly methylated in 75% of tumor samples, whereas no methylation is detected in healthy lung tissues. Additionally, the elevated methylation of the *HOXB3/HOXB4* region is particularly evident in metastatic tumors, such as those in the brain and adrenal glands. This aberrant methylation is more pronounced in tumors with metastatic potential compared with nonmetastasizing tumors, underscoring its potential involvement in cancer progression. As a result, *HOXB3/HOXB4* methylation has demonstrated high sensitivity and specificity in detecting lung cancer, underscoring its potential as a diagnostic biomarker [396]. On the other hand, high *HOXB3* expression has been reported in lung cancer, particularly in LUAD, where it is consistently associated with poor clinical outcomes and reduced OS. Additionally, in vitro studies have demonstrated that *HOXB3* downregulation inhibits LUAD cell proliferation and enhances their susceptibility to apoptosis. Computational analyses using tools like tumor immune estimation resource (TIMER) and tumor immune single‐cell database (TISIDB) further reveal a strong association between *HOXB3* expression and tumor immunity such as immune checkpoint molecules (ICMs). These findings highlight *HOXB*3's oncogenic role and its link to tumor immunity in LUAD [438]. In addition, recent studies have also shown that *HOXB4* promoter hypermethylation is frequent in LUAD, associates with early‐stage disease and poorer survival, and carries diagnostic/prognostic value [442].

Dysregulation of *HOXB7* expression has been implicated in tumorigenesis and metastasis across various cancers. While its role in facilitating cancer progression is documented, the specific mechanisms by which *HOXB7* expression levels contribute to malignancy remain largely unclear. Notably, *HOXB7* overexpression has been observed in multiple cancer types, particularly lung and breast cancers biology [376, 443]. For example, in breast cancer cells, *HOXB7* overexpression has been shown to drive the EMT, thereby enhancing tumor progression and metastasis, particularly to the lungs. In vivo and in vitro experiments have shown that this metastatic process may involve the regulation of the TGF‐β/SMAD3 signaling pathways [443]. Remarkably, TGF‐β signaling plays a complex role in cellular processes such as cell proliferation and differentiation, acting through both canonical SMAD‐dependent and noncanonical pathways. This signaling is initiated when one of the three TGF‐β isoforms (TGF‐β1, TGF‐β2, or TGF‐β3) binds to transmembrane serine/threonine kinase receptors. Additionally, dysregulation of the TGF‐β pathway is frequently observed in various cancers [160, 444]. The results of a study suggest that *HOXB7* overexpression is directly linked to elevated TGFβ2 expression, indicating a potential role for *HOXB7* upregulation in modulating TGFβ signaling. *HOXB7* overexpression has been shown to attract tumor‐associated macrophages and drive an M2 phenotype via TGFβ2 signaling, which may contribute to metastatic progression. Notably, silencing TGFβ2 in the breast cancer cell lines overexpressing *HOXB7* significantly reduces lung metastasis, emphasizing the critical interplay between *HOXB7* and TGFβ2 in regulating tumor metastasis, particularly to lung. In LUAD, current evidence indicates that the higher level of *HOXB7* expression is associated with increased inferred fractions of M0/M1 macrophages and decreased levels of resting mast cells and dendritic cells [17, 443]. Therefore, *HOXB7* is significantly overexpressed in NSCLC and LUAD, suggesting its role as a key oncogene involved in carcinogenesis. Particularly, *HOXB7* expression is significantly elevated in LUAD tissues compared with normal lung epithelial tissues. This upregulation is linked to increased cell proliferation and enhanced metastatic potential, contributing to poor clinical outcomes, advanced tumor stages, and reduced patient survival rates. Mechanistically, HOXB7 promotes cell growth and tumor progression by activating the MAPK and *PI3K/Akt* signaling pathways. Conversely, studies have demonstrated that silencing *HOXB7* effectively suppresses associated metastatic processes in LUAD [443]. Moreover, the regulation of *HOXB7* expression is intricately connected to taurine‐upregulated gene 1 (*TUG1*), an lncRNA that binds to the PRC2, facilitating H3K27 trimethylation at the *HOXB7* promoter locus. In contrast, *TUG1* downregulation disrupts PRC2 binding, leading to the loss of H3K27 trimethylation and increased *HOXB7* expression. The expression of *TUG1* is also directly regulated by the p53 protein, as *TUG1* serves as a transcriptional target of p53. This regulatory relationship between p53 and *TUG1* also suggests that p53 plays a role in influencing the expression of *HOXB7*. In NSCLC, *TUG1* had been detected to be downregulated, leading to *HOXB7* overexpression and promoting tumor proliferation through the MAPK and PI3K/Akt pathways. Correspondingly, reduced *TUG1* expression is associated with advanced tumor stage and lower OS. These results indicate the critical role of the p53/*TUG1/PRC2/HOXB7* axis in NSCLC tumorigenesis [445].

*HOXB8* overexpression has been documented in several cancers, including CRC, and more recently, it has been found to be significantly upregulated in NSCLC tissues compared with adjacent normal tissues. This overexpression is strongly correlated with tumorigenesis and lymph node metastasis, which are linked to advanced tumor stages and poor survival rates. Functionally, *HOXB8* drives NSCLC progression by promoting EMT. In vitro studies on NSCLC cell lines have demonstrated that silencing *HOXB8* via siRNA suppresses EMT, evidenced by an increase in *E‐cadherin* expression and downregulation of *N‐cadherin*, *MMP‐2*, and *vimentin*. These findings highlight the potential of *HOXB8* knockdown as a therapeutic strategy to inhibit EMT and reduce NSCLC progression [69, 446].

As described earlier, *HOXB9* is consistently overexpressed in various cancers, including breast and lung cancer, where it functions as a key oncogene. Studies on lung cancer tissue samples demonstrate significantly elevated *HOXB9* expression in NSCLC tissues compared with the noncancerous tissues. Specifically, *HOXB9* overexpression has been detected in 21.3% of LUAD cases and is strongly associated with tumor progression, advanced TNM stages, and poor clinical outcomes, including reduced OS. Notably, its overexpression correlates with lower 5‐year survival rates, underscoring its role in aggressive tumor phenotypes [447, 448, 449]. In lung cancer, *HOXB9* drives tumor‐promoting processes through mechanisms such as the Wnt pathway, the AMP kinase (*AMPK*)–*HOXB9*–Kirsten rat sarcoma viral oncogene homolog (KRAS) axis, and the EMT. By promoting EMT, *HOXB9* overexpression is closely linked to larger tumor sizes and enhances the migratory and invasive abilities of NSCLC cells, directly contributing to metastases, including lymph node involvement and distant organ metastases, particularly to the brain and bones [356, 357, 447, 448]. In brain metastases, the role of *HOXB9* is closely tied to its ability to activate the EMT. This process enables tumor cells to acquire greater mobility and invasive properties, allowing them to disseminate into the bloodstream and colonize other organs. Interestingly, elevated levels of *HOXB9* have been linked to compromised blood–brain barrier (BBB) integrity through the disruption of ECs adhesion junctions via the upregulation of *MMP‐9* expression. As a positive regulator of *MMP‐9*, its overexpression enhances the metastatic potential of NSCLC cells. The endopeptidase activity of MMP‐9 facilitates the degradation of ECM proteins and diminishes the expression of junctional proteins, ultimately weakening the BBB and allowing cancer cells to pass into the brain. This dual mechanism enhancing EMT and disrupting BBB integrity highlights the pivotal role of *HOXB9* in driving metastasis of NSCLC to the brain. Clinical and experimental evidence consistently demonstrates that *HOXB9* is significantly upregulated in the majority of primary NSCLC tumor samples. Moreover, patients with elevated HOXB9 expression in these tumors are more likely to have shorter brain metastasis‐free survival compared with those with lower *HOXB9* expression, underscoring its potential role in NSCLC progression and metastasis [447]. Similarly, in addition to its role in lung cancer metastasis, *HOXC9* has been reported to involve in breast cancer metastasis, particularly to the lungs. In vivo studies reveal that *HOXC9* upregulation is associated with high tumor grade and promotes tumor growth and angiogenesis by upregulating the expression of angiogenic factors such as VEGF and TGF‐β. These factors drive cell motility, mesenchymal transitions, and alter the TME, enhancing neovascularization and facilitating distal metastasis to the lungs [449].

###### 4.3.1.1.4 *HOXC* Cluster's Role in Lung Cancer

Several genes within the HOXC cluster have been implicated in the development and progression of various cancers, including lung cancer, and have attracted significant research interest. Among these, members such as *HOXC8* and *HOXC13* are notably overexpressed in primary lung tumors and lung cancer cell lines, underscoring their potential roles in tumorigenesis [372]. Therefore, overexpression of certain genes in this cluster have been observed in lung cancer studies, contributing to its pathogenesis, as detailed below. *HOXC4* has been identified as an oncogene involved in tumorigenesis, contributing to the development and progression of multiple cancers, including lung cancer. Studies integrating data from the cancer genome atlas (TCGA) and genotype tissue‐expression (GTEx) datasets have demonstrated that *HOXC4* is significantly upregulated in at least 21 different cancer types, highlighting its potential role in carcinogenesis. Notably, its abnormal overexpression has been observed in lung cancer, particularly in LUAD and LUSC, as well as in other malignancies such as colorectal adenocarcinoma and HNSCC [450, 451]. In lung cancer, evidence suggests that *HOXC4* overexpression is associated with poor prognosis, including reduced OS, disease‐specific survival (DSS), disease‐free interval, and progression‐free interval (PFI). This correlation is particularly strong in early‐stage squamous cell lung cancer (SqCLC), where *HOXC4* has one of the most pronounced impacts on poor prognosis and survival outcomes, especially OS. Additionally, in LUAD, studies indicate that aberrant *HOXC4* expression correlates with mutations in mismatch repair (MMR) genes such as *mutL homolog 1* (*MLH1*), *mutS homolog 2* (*MSH2*), *MSH6*, and *postmeiotic segregation increased 2* (*PMS2*). This suggests that the elevated level of *HOXC4* expression may influence DNA repair pathways by regulating MMR genes, which are critical for repairing DNA mismatch errors, thereby enhancing the survival of related malignant cells [450, 451].

Aberrant *HOXC6* expression has been explored to participate in the progression of various cancers, including BLCA and lung cancer. In lung cancer, *HOXC6* overexpression has been observed across multiple histological subtypes, including NSCLC, LUAD, and LUSC, with approximately 66.6% of NSCLC tumors exhibiting elevated *HOXC6* levels compared with healthy controls, suggesting its potential role as a biomarker. This overexpression is associated with enhanced cell proliferation, invasion, migration, lower immune scores, and poor clinical outcomes, including poor OS, advanced tumor size (T), and lymph node involvement (N) based on the TNM staging system. In cancer cells, prior studies have indicated that *HOXC6* upregulation contributes to chemotherapy resistance by activating the *multidrug resistance1* (*MDR1*) gene promoter and influencing other ATP‐binding cassette (ABC) transporters, specifically ABCG2 and MRP1, which expel chemotherapeutic agents from cancer cells [452]. Moreover, current evidence indicates that higher HOXC6 expression is associated with an increased inferred fraction of M0 macrophages, alongside decreased levels of resting mast cells and dendritic cells resting in LUAD [17]. It should be noted that HOXC6 also plays a crucial role in determining the effectiveness of targeted therapies, particularly gefitinib (Gef), which is an epidermal growth factor receptor‐tyrosine kinase inhibitor (EGFR‐TKI) commonly prescribed for the treatment of NSCLC. Correspondingly, in vitro studies on Gef‐resistant NSCLC cells demonstrate that knocking down *HOXC6* expression not only enhances Gef sensitivity but also leads to increased apoptosis, G2/M phase cell cycle arrest, and suppression of cell migration [453]. Interestingly, it should be noted that patients with LUAD exhibiting upregulation in the expression of *HOXC6* have been found to be more responsive to chemotherapeutic agents such as camptothecin and vorinostat. These results demonstrate the importance of considering *HOXC6* expression as a potential therapeutic target for improving treatment strategies in lung cancer [453, 454]. In addition to its role in modulating the expression of various genes associated with lung cancer, HOXC6 has been identified as a crucial regulator of several genes that contribute to tumorigenesis. It affects the expression of key tumorigenesis‐related genes such as *WNT6*, *MMP‐2*, and *secreted protein acidic and rich in cysteine* (*SPARC*). Furthermore, *HOXC6* is regulated by *miR‐27* *a*, which directly influences its expression levels [453, 454]. In lung cancer, research indicates that while *HOXC6* is often upregulated, *miR‐27a* is frequently downregulated in NSCLC, particularly LUAD; this imbalance is linked to aggressive malignancy and poor clinical outcomes in these patients. In contrast, in vitro and in vivo studies on Gef‐resistant NSCLC cells have indicated that the upregulation of miR‐27a can reverse Gef resistance by suppressing *HOXC6* expression. Correspondingly, miR‐27a downregulates *HOXC6*, which subsequently influences downstream drug resistance‐related markers such as *ABCG2*, *Bcl‐2*, and *N‐cadherin*, all of which are implicated in drug resistance [453].

*HOXC8* overexpression has been observed in various cancers, including breast and lung cancer, where its elevated levels are associated with disease progression and metastasis. In lung cancer, *HOXC8* is prominently expressed in NSCLC, particularly in LUAD and LUSC, with significantly higher mRNA levels compared with healthy lung tissues. Studies in NSCLC cell lines have shown that HOXC8 is likely involved in regulating gene expression; its overexpression in these cell lines leads to the upregulation of genes such as *TGFβ1* and *vimentin*, while downregulating *E‐cadherin*, thereby indicating its role as a transcriptional activator [379]. TGFβ1 is a well‐known cytokine with a critical role in cancer cell proliferation, migration, and metastasis across various cancer types. Notably, *TGFβ1* expression is nearly doubled in NSCLC compared with normal tissues and is strongly correlated with tumorigenesis, including advanced TNM stages and lymph node metastases. In NSCLC, *HOXC8*‐mediated *TGFβ1* upregulation directly contributes to enhancement cancer cell proliferation and anchorage‐independent growth [455, 456]. *HOXC8* upregulation is also implicated in the EMT process in lung cancer by downregulating *E‐cadherin* and upregulating *TGFβ1* and *vimentin*. The downregulation of *E‐cadherin* via HOXC8 is particularly associated with increased tumor growth, migration, and metastasis in NSCLC, indicating its significant role in cancer progression and poor clinical outcomes, including worse RFS. Moreover, *HOXC8* upregulation contributes to increased chemoresistance in NSCLC. Conversely, in vitro studies have shown that silencing *HOXC8* reduces chemoresistance and enhances NSCLC cell sensitivity to chemotherapy drugs such as cisplatin [379, 457]. Overexpression of *HOXC9* has been observed in various cancers, including lung cancer, although its expression levels in lung cancer remain controversial. While many studies report elevated *HOXC9* expression, some findings suggest that *HOXC9* may exhibit aberrant methylation in lung cancer samples, particularly when analyzed alongside other genes such as *RASSF1A* and *APC*. These genes often display aberrantly methylated in their promoter regions, indicating a complex regulatory role for HOXC9 in lung cancer progression. Interestingly, the methylation status of HOXC9 and associated genes appears to be more characteristic of advanced stages of NSCLC compared with early stages [458, 459]. In LUSC, hypermethylation of the *HOXC9* gene is observed at a frequency of 66%, whereas in Stage I NSCLC samples, *HOXC9* methylation occurs in 13.71% of cases. These findings suggest that *HOXC9* hypermethylation may serve as a potential biomarker for specific lung cancer subtypes and stages [459, 460]. On the other hand, *HOXC9* overexpression in lung cancer, particularly in NSCLC subtypes such as LUAD, is widely reported and associated with poor clinical outcomes. *HOXC9* overexpression has been confirmed in LUAD tumor tissues compared with adjacent noncancerous tissues, underscoring its role as a prognostic biomarker. In LUAD, upregulated *HOXC9* expression correlates with advanced tumor stages and reduced OS, highlighting its oncogenic potential. However, the association between *HOXC9* expression levels and OS is absent in LUSC. As an oncogene, *HOXC9* overexpression contributes to carcinogenesis in lung cancer by promoting cell proliferation, migration, and invasion, particularly in LUAD [458, 461, 462]. Moreover, *HOXC9* overexpression in LUAD is associated with immune suppression, as evidenced by reduced CD8+ T cell activation and diminished IFN‐γ production, whereas its knockdown enhances both, demonstrating its suppressive role in the TME. In LUAD, combining *HOXC9* knockdown with immune checkpoint inhibitor therapies, such as PD‐1 blocking therapies, has demonstrated synergistic effects in reducing tumor growth and enhancing antitumor immune responses through pronounced activation of CD3+/CD8+/IFN‐γ+ T cells, which are critical for targeting and killing lung cancer cells [461]. In lung cancer, *HOXC9* expression is also tightly regulated by specific circRNAs‐miRNA interactions [458, 462]. Notably, *hsa_circ_0020123* functions as a miR‐495 sponge, preventing miR‐495 from binding to the *HOXC9* 3′‐UTR and thereby promoting HOXC9 expression. This regulatory mechanism enhances tumor cell migration and proliferation, as demonstrated by the reversal of miR‐495‐induced suppression upon *HOXC9* overexpression. Elevated *hsa_circ_0020123* expression is consequently associated with poor prognoses in NSCLC patients [458, 462]. Similarly, circCENPF functions as a hsa‐miR‐184 sponge, increasing *HOXC9* expression and driving cell proliferation, migration, and invasion in NSCLC, particularly in LUAD. These findings underscore the significance of circRNA–miRNA–*HOXC9* axis in NSCLC progression and their potential for therapeutic intervention [462, 463].

Aberrant expression of *HOXC10* has been reported in various cancers, including lung cancer, where its elevated levels are strongly linked to tumorigenesis. Studies indicate that *HOXC10* expression is significantly higher in tumor tissues compared with noncancerous tissues in NSCLC, particularly in LUAD patients. Overexpression of *HOXC10* in LUAD is strongly associated with aggressive disease features, including advanced clinical stages and reduced distant metastasis‐free survival, underscoring its role in tumor progression and metastasis [464]. As an oncogene, elevated *HOXC10* drives tumor progression in LUAD by promoting angiogenesis through VEGFA upregulation and increased microvessel density, which correlates with aggression, metastasis, advanced stages, and poor outcomes. Beyond its role in angiogenesis, *HOXC10* upregulation has been reported to interact with the TME and immune regulatory pathway [313, 465, 466]. As mention earlier, research indicates a direct correlation between *HOXC10* expression and metastasis in lung cancer patients. Notably, in cases of bone metastasis, *HOXC10* expression is strongly associated with the presence of *KRAS* mutations, further emphasizing its role in lung cancer metastasis [464, 465]. *KRAS*, part of the sarcoma viral oncogene homolog (RAS) gene family, is the most frequently mutated oncogene in NSCLC, particularly in cases with bone metastases. These mutations, particularly in patients suffering bone metastases from LUAD, are linked to poor outcomes and have proven resistant to related‐targeted therapies such as mitogen‐activated protein kinase (MEK) inhibitors [464, 467, 468]. Experimental studies have shown that in NSCLC with *KRAS* mutations, around 50% of cases display abnormal expression of *HOXC10*. This overexpression is mainly linked to defects in *PRC2*, a vital histone methyltransferase that plays a significant role in regulating *HOX* gene expression. *PRC2* is frequently associated with various cancers, functioning both as a tumor suppressor and as an oncogene [464, 469, 470]. Consequently, defects in PRC2 genes play a pivotal role in the dysregulation of *HOXC10*. Specifically, the downregulation of PRC2 results in the upregulation of *HOXC10* in NSCLC, contributing to enhancement *HOXC10*’s role in tumor progression. HOXC10 interacts with the promoter region of *nucleotide‐binding oligomerization domain 1* (*NOD1*) at its specific binding‐site, leading to enhanced expression of *NOD1*. This interaction plays a crucial role in the progression of KRAS‐mutant lung cancer bone metastasis by activating the NOD1/ ERK signaling pathway. Studies have demonstrated that suppressing *HOXC10*, particularly in combination with a *STAT3* inhibitor, significantly inhibits the proliferation and migration of lung cancer cells with KRAS mutations in both in vivo and in vitro models. This suppression effectively reduces the metastatic spread of these cells to osteolytic bone sites by disrupting key mechanisms driving tumor progression. Specifically, *HOXC10* inhibition has been shown to impair the NOD1/ERK signaling pathway, which plays a critical role in reprogramming the EMT and modulating the bone microenvironment [466].

*HOXC11* has been found to be involved in the progression of various cancers, including renal cell carcinoma and CRC, where its overexpression correlates with poor clinical outcomes. In lung cancer, particularly in LUAD and LUSC, recent studies emphasize its significant role. Both of experimental and bioinformatics analyses demonstrate that *HOXC11* expression is significantly elevated in lung cancer tissues compared with the noncancerous tissues, with its overexpression especially notable in LUAD and LUSC. This upregulation is strongly associated with tumorigenesis, aggressive tumor phenotype, and poor clinical outcomes, including shorter OS in LUAD, highlighting its potential as a prognostic biomarker [471]. In particular, upregulation of *HOXC11* in LUAD cells has been reported to promote tumorigenesis and enhance malignant phenotypes, significantly affecting key characteristics such as cell proliferation, migration, and invasion. Elevated levels of *HOXC11* are also linked to increased colony formation, as well as promotion of subcutaneous and lung metastasis, thereby contributing to the overall aggressiveness of the tumor. Moreover, *HOXC11* expression is regulated through several mechanisms, including *IκB kinase α* (*IKKα*), and miR‐1197 [471, 472]. In LUAD, *HOXC11* expression has been detected to be tightly regulated by *IKKα*. *IKKα* has been detected in various types of cancer and performs multiple functions through both NF‐κB‐dependent and independent mechanisms. In canonical NF‐κB signaling, IKKα, as part of *IKK* complex, phosphorylates IκBα, leading to its degradation via the ubiquitin‐proteasome pathway and subsequent nuclear translocation of NF‐κB dimers. Additionally, IKKα can be influenced in the regulation of cell proliferation by stabilizing β‐catenin to enhance cyclin D1 expression or by phosphorylating cyclin D1 to induce its degradation [161, 473]. Elevated levels of *IKKα* are directly associated with the posttranscriptional regulation of *HOXC11*, enhancing the levels of *HOXC11* protein and promoting its stability by preventing degradation through the ubiquitin‐proteasome pathway. These results suggest the critical role of IKKα in promoting *HOXC11* expression and driving tumor progression in lung cancer [471, 473]. *HOXC11* also regulates the expression of *sphingosine kinase 1* (*SPHK1*) (HOXC11 downstream target gene) by binding to its promoter region. *SPHK1* is notably upregulated in NSCLC, particularly in LUAD, where its elevated expression correlates with a more malignant phenotype. Experimental studies indicate that inhibiting *SPHK1* in LUAD cells with stable *HOXC11* overexpression reduces migration and invasion, emphasizing the importance of the *HOXC11‐SPHK1* axis in lung cancer progression. Moreover, the elevated levels of *HOXC11* and *SPHK1* are strongly associated with poor prognosis in LUAD patients [473]. Interestingly, previous studies have revealed a distinct regulation and function for *HOXC11* in lung cancer, where it is downregulated by miR‐1197. In vitro studies have demonstrated that miR‐1197 is upregulated in NSCLC, and its expression inversely regulates *HOXC11* expression in NSCLC, leading to *HOXC11* downregulation. Conversely, silencing *miR‐1197* results in the upregulation of *HOXC11*, which, in turn, inhibits NSCLC cell proliferation and migration. These studies indicate that miR‐1197 plays a tumor‐suppressing role when downregulated and provides a novel epigenetic regulatory axis involving miR‐1197 and *HOXC11* in lung cancer [472].

*HOXC13* has been identified as a potential oncogene in lung cancer, with experimental and bioinformatics analyses confirming its significant upregulation in LUAD and LUSC compared with normal tissues. In vitro studies show that *HOXC13* mRNA and protein levels are notably higher in LUAD cell lines than the control cell lines. By analyzing data from the UCSC Xena and GEPIA databases on *HOXC13* expression, it was shown that *HOXC13* is consistently overexpressed across all stages of lung cancer. Clinically, this elevated expression is strongly associated with poor prognosis and adverse outcomes, suggesting *HOXC13* as a promising prognostic biomarker [474, 475]. *HOXC13* exerts its oncogenic effects in lung cancer by regulating the expression of key oncogenes, *CCND1* (*Cyclin D1*) and *CCNE1* (cyclin E1), which are crucial for tumor cell proliferation and are associated with poor clinical outcomes. Both genes are upregulated in lung cancer and correlate with enhanced metastasis and poor prognosis, highlighting their role as critical prognostic biomarkers. Specifically, the upregulation of *CCND1* promotes cell proliferation, invasion, and migration [476, 477, 478]. Moreover, elevated *CCNE1* expression has been observed in NSCLC, particularly LUAD, and is strongly associated with tumor growth [478, 479]. Thus, *HOXC13*‐mediated regulation of *CCND1* and *CCNE1* significantly contributes to lung cancer progression [476]. In lung cancer, upregulation of *CCND1* leads to promote cell cycle progression into the S phase, while CCNE1 facilitates the transition from the G1 phase to the S phase. In LUAD, evidence indicates that elevated *HOXC13* expression strongly correlates with the upregulation of both genes, enhancing cell proliferation and reducing G1‐phase arrest. This promotes continuous cell cycle progression and tumor growth, particularly in LUAD. Conversely, silencing *HOXC13* downregulates both *CCND1* and *CCNE1*, inducing G1‐phase arrest and inhibiting LUAD progression [476, 477].

In lung cancer, the expression of *HOXC13* has also been demonstrated to be modulated by miR‐141 and the LncRNA *HOXC‐AS2*. In LUAD, miR‐141 suppresses the expression of *HOXC13* by directly binding to its 3′‐UTR region, which subsequently leads to the downregulation of *CCND1* and *CCNE1*. This regulation enables miR‐141 to effectively inhibit cell proliferation in LUAD. Notably, studies have shown that the upregulation of *HOXC13* can counteract the inhibitory effects of miR‐141, underscoring the critical role of elevated *HOXC13* levels in promoting cell growth by mitigating miR‐141‐mediated suppression. The lncRNA *HOXC‐AS2* is located in close proximity to its target gene, *HOXC13*. Both experimental and bioinformatics analyses have confirmed a positive regulatory relationship between LncRNA *HOXC‐AS2* and *HOXC13*, leading to elevated *HOXC13* at both the mRNA and protein levels, particularly in NSCLC. Importantly, *HOXC13* has been identified as a coexpressed target of LncRNA *HOXC‐AS2*, demonstrating the interdependence of their activities and emphasizing the critical role of the LncRNA *HOXC‐AS2/HOXC13* regulatory axis in NSCLC pathogenesis [475, 476].

###### 4.3.1.1.5 *HOXD* Cluster's Role in Lung Cancer

Several genes within the *HOXD* cluster have been implicated in the development and progression of various cancers, including lung cancer. This subfamily exhibits context‐dependent functions, acting as either oncogenes or tumor suppressors depending on the type of cancer. Therefore, dysregulated *HOXD* expression has been associated with key clinical and pathological features, emphasizing its contribution to tumor growth and malignancy [480, 481]. Many genes in this subfamily have been reported to exhibit altered regulation in lung cancer, as discussed in greater detail below.

*HOXD1* is significantly downregulated in LUAD tissues and cell lines compared with their normal counterparts, and this reduced expression is associated with poor prognosis and lower OS in patients. DNA methylation of the *HOXD1* promoter region has been identified as a key mechanism driving its downregulation. However, studies in LUAD have demonstrated that upregulation of *HOXD1* can function as a tumor suppressor. Its overexpression has been shown to inhibit cell proliferation, migration, and invasion in both in vitro and in vivo models, particularly suppressing tumor growth in mouse models. Furthermore, elevated levels of *HOXD1* act as a TF, promoting the expression of *BMP2* and *BMP6*, which are implicated in the inhibition of LUAD progression. Although *HOXD1* downregulation in LUAD has been reported, the key pathways and mechanisms associated with its aberrant methylation remain poorly understood [481].

*HOXD3* has been the focus of conflicting reports regarding its expression levels in lung cancer. Studies on SCLC tissue samples report that *HOXD3* is overexpressed in tumors originating from primary or metastatic sites, but not in noncancerous lung tissues, emphasizing its role in lung metastasis. However, other studies have been found that *HOXD3* undergoes abnormal promoter methylation in lung cancer, particularly LUAD. These findings underscore the pivotal role of *HOXD3* in lung cancer progression [481, 482, 483]. In vitro studies on lung cancer cell lines have demonstrated a direct association between elevated *HOXD3* expression and increased invasion and metastasis, identifying *HOXD3* as a potential metastatic gene. Therefore, the expression of the *HOXD3* gene in lung cancer cell lines has been closely linked to alterations in cell adhesion, which directly contribute to promoting metastasis. Evidence suggests that elevated *HOXD3* levels drive lung cancer metastasis through the regulation of genes’ expression involved in cell adhesion and metastasis. Specifically, *HOXD3* overexpression is associated with the reduced expression of *E‐cadherin* and *plakoglobin*, both critical for maintaining epithelial integrity and cell–cell adhesion, while concurrently upregulating integrins and also *N‐cadherin* [484, 485]. E‐cadherin, a crucial cell–cell adhesion protein, plays an essential role in maintaining epithelial integrity. However, it is significantly downregulated in lung cancer, particularly in NSCLC, which can disrupt this important function [486, 487]. Similarly, plakoglobin, a key component of adherens junctions and desmosomes, is vital for the regulation of cell–cell adhesion. Studies indicate that *plakoglobin* expression is significantly downregulated in NSCLC cell lines, which contributes to their increased metastatic potential. Conversely, restoring plakoglobin levels has been shown to effectively suppress proliferation, invasion, and migration in lung cancer cells, indicating a tumor suppressor role of plakoglobin in lung cancer [488]. In contrast, aberrant expression of *N‐cadherin* is tightly associated with enhanced motility and malignancy of cancer cells. Its upregulation facilitates cancer progression by driving metastasis and invasion while promoting interactions between cancer cells and the extracellular microenvironment [489, 490]. Remarkably, this dual regulatory effect of *HOXD3* underscores its role in enhancing the invasive and metastasis potential of lung cancer cells [485].

On the other hand, aberrant methylation of the *HOXD3* gene has been specifically reported in various studies. Analyses of samples from patients with LUAD have demonstrated that the aberrant methylation of *HOXD3* is specific to cancerous tissues and is not present in healthy lung tissues, highlighting its potential as a cancer‐specific epigenetic alteration. Similarly, research utilizing blood‐based liquid biopsy samples from cancer patients, particularly those with lung cancer, has identified *HOXD3* as one of the genes undergoing abnormal methylation in its promoter region. Moreover, studies have shown that circulating methylated *HOXD3* levels, along with other genes such as *RASSF1A*, are significantly elevated in SCLC compared with NSCLC. Panels incorporating methylated *HOXD3* and *RASSF1A* achieve high sensitivity (75%) and specificity (88%) in distinguishing between lung cancer subtypes, from SCLC to NSCLC. Furthermore, elevated levels of methylated *HOXD3* are associated with node‐positive disease, metastatic dissemination, and an increased disease‐specific mortality rate. Consequently, methylated *HOXD3* has emerged as a potential biomarker for both diagnosis and prognosis in lung cancer [481, 482, 483].

The expression of *HOXD8* in lung cancer has demonstrated variability across studies. While certain investigations have reported upregulated *HOXD8* expression, others have highlighted abnormal methylation of its promoter region. These findings suggest a complex regulatory mechanism influencing *HOXD8*’s role in lung cancer pathogenesis [436, 491]. Notably, *HOXD8* is implicated in processes such as cell migration and has been found to be methylated in LUADs. This methylation is associated with its downregulation, which is observed more frequently in the metastatic tumors than in primary ones, underscoring its critical involvement in metastasis [436]. On the other hand, *HOXD8* overexpression has been observed in various cancers, including ovarian and lung cancers, where it plays a significant role in tumor progression. In lung cancer, *HOXD8* expression is markedly elevated in tumor tissues compared with adjacent noncancerous tissues, indicating its potential oncogenic role. Studies have demonstrated that HOXD8 protein levels are particularly increased in NSCLC. Consequently, in NSCLC, this upregulation is correlated with increased cancer cell proliferation, the formation of CSCs, and enhanced migratory capacity. These findings underscore the oncogenic role of *HOXD8* in driving the aggressive behavior of lung cancer cells. *HOXD8* expression in lung cancer has been demonstrated to be modulated by *miRNAs*. Specifically, *HOXD8* has been identified as a direct target gene of *miR‐142‐5p* and miR‐520a‐3p, suggesting that these *miRNAs* play a crucial role in the regulating of *HOXD8* expression levels in lung cancer cells. Studies have further demonstrated a direct correlation between the expression levels of *miR‐142‐5p* and *miR‐520a‐3p* and the regulation of *HOXD8*, which significantly contributes to the resistance of lung cancer cell lines to Gef [491, 492]. Therefore, HOXD8 and *miR‐142‐5p* play critical roles in modulating Gef sensitivity in NSCLC cell lines. Studies have shown that *miR‐142‐5p* binds to the 3′‐UTR of HOXD8 mRNA, resulting in the downregulation of *HOXD8* expression. Consequently, overexpression of *miR‐142‐5p* significantly reduces protein expression level of *HOXD8*, whereas its downregulation results in *HOXD8* upregulation, demonstrating a clear negative correlation between their expressions. Notably, *miR‐142‐5p* enhances Gef‐induced apoptosis in lung cancer cells by activating the mitochondria‐dependent apoptosis pathway, a process modulated through *HOXD8* regulation. Therefore, studies indicate that the coexpression of *miR‐142‐5p* and *HOXD8* significantly reduces the levels of apoptosis‐related proteins such as *Bax*, *cleaved‐caspase3*, and *cleaved‐PARP*, potentially influencing the cells response to Gef [492]. Studies on NSCLC and SCLC tissues and the related‐cell lines have demonstrated distinct roles of *miR‐520a‐3p* expression in NSCLC. While *miR‐520a‐3p* expression is decreased in NSCLC, its upregulation acts as a tumor suppressor by directly targeting *HOXD8*. Overexpression of *miR‐520a‐3p* not only degrades *HOXD8* mRNA but also inhibits cancer cell proliferation and CSC phenotypes specifically in NSCLC, with no notable effect in SCLC. Moreover, overexpression of *miR‐520a‐3p* leads to downregulate the level of the mesenchymal–epithelial transition (MET), while *HOXD8* has an adverse effect on its expression and leads to an increase in its expression [491]. MET, a proto‐oncogene expressed on epithelial cells across various organs, plays a pivotal role in biological processes such as embryonic development and tissue regeneration. Notably, MET–hepatocyte growth factor pathway is one of the well‐known pathways involved in several types of cancer, particularly lung cancer. Therefore, dysregulation of the MET pathway, often associated with mutations or overexpression, contributes to tumorigenesis and cancer progression through pathways such as Wnt/β‐catenin signaling, which drives proliferation, metastasis, and invasion in cancer cells [493, 494]. In NSCLC, aberrant MET dysregulation is frequently reported and is probably linked to resistance against Gef. Moreover, *HOXD8*‐mediated MET upregulation correlates with Gef resistance, whereas suppression of the MET has been shown to reverse this resistance in NSCLC cell lines [491, 493].

The aberrant expression of *HOXD9* has been detected in various cancers, including glioblastomas, gastric cancer, and lung cancer. In NSCLC, *HOXD9* is significantly upregulated in tissues and cell lines compared with normal counterparts, correlating with poor prognosis and lower OS rate. Therefore, overexpression of *HOXD9* in NSCLC directly contributes to tumorigenesis such as cell proliferation, migration, invasion, and apoptosis inhibition. In addition, its upregulation is correlated with metastasis in NSCLC [494, 495, 496]. Research suggests that *immunoglobulin TF 2* (*ITF2*), also known as TCF4, plays a crucial role in the regulation of *HOXD9* expression. *ITF2*, known as a downstream target of the Wnt/β‐catenin signaling pathway, is a key TF whose reduced expression is frequently observed in lung cancer. *ITF2* expression is often reduced in lung cancer, but its overexpression inversely correlates with *HOXD9* levels, improving patient outcomes by inhibiting tumor progression. This regulation appears to be mediated through an alternative mechanism involving Wnt pathway activation. Consequently, upregulation of *ITF2* is associated with lower *HOXD9* expression and improved OS in NSCLC patients. In vivo and in vitro studies further support the idea that silencing *HOXD9* suppresses metastasis, invasion, cell proliferation, and cell migration, while inducing apoptosis in NSCLC [495, 497]. Moreover, evidence indicates that the silence of *HOXD9* in NSCLC cell lines suppresses proliferation by inducing G1 cell cycle arrest. This process is characterized by reduced expression of *cyclin E* and *cyclin B1*, along with increased expression of *p53*, a critical regulator of DNA repair and cell cycle progression. *Cyclin E*, a key regulator of the G1/S transition in the cell cycle, is frequently overexpressed in tumors, contributing to dysregulated cell cycle progression in lung cancer. Similarly, *cyclin B1* (also known as *CCNB1*), critical for the G2‐M phase transition, is typically low under normal conditions but is upregulated during the transition and associated with malignancy in various cancers, including NSCLC, particularly in LUAD. Consequently, the aberrant expression of both of them drives uncontrolled cell cycle progression [495, 498, 499]. *HOXD9* also contributes to the regulation of angiogenesis and immune evasion in NSCLC. Specifically, it regulates the expression of *ANGPT2* and *programmed death ligand‐1* (*PD‐L1*), both of which are closely associated with tumor progression. *ANGPT2*, a proangiogenic factor, works alongside *VEGF* to significantly contribute to tumor vascularization and is upregulated in several cancers, including lung cancer. Elevated plasma levels of *ANGPT2* have been observed in NSCLC patients, with further increases noted following tumor resection [495, 500]. The overexpression of *HOXD9* is positively associated with elevated levels of *ANGPT2*, thereby promoting metastasis and angiogenesis in lung cancer. In contrast, downregulation of HOXD9 leads to a reduction in *ANGPT2* expression. *ANGPT2*, a proangiogenic factor, besides *VEGF* plays a significant role in tumor vascularization and is upregulated in several cancers, including lung cancer. Elevated plasma levels of ANGPT2 have been observed in NSCLC patients, with further increases noted after tumor resection. Additionally, *HOXD9* inhibition promotes apoptosis by activating *caspase‐3* and *polymerase 1* (*PARP1*) cleavage, disrupting DNA repair and inducing apoptosis [495, 500]. In addition, both in vivo and in vitro studies on lung cancer cell lines have demonstrated that *HOXD9* upregulates *PD‐L1*, a critical ICM. This upregulation significantly aids tumor cells in evading recognition by the immune system, particularly T cells, thereby contributing to unfavorable clinical outcomes in these patients. In contrast, silencing *HOXD9* markedly reduces *PD‐L1* expression, underscoring its role in immune evasion mechanisms in NSCLC [495]. As previously noted, *HOXD9* plays a significant role in lung cancer metastasis. Among the mechanisms linking HOXD9 to this process is its ability to promote increased glycolysis. The metabolic reprogramming observed NSCLC, characterized by the Warburg effect, is closely tied to the transcriptional activation of *6‐phosphofructo‐2‐kinase/fructose‐2, 6‐bisphosphatase 3* (*PFKFB3*) by HOXD9. By directly binding to the promoter region of *PFKFB3*, a critical enzyme in glycolysis, HOXD9 enhances its expression, establishing a positive relationship between *HOXD9* overexpression and *PFKFB3* upregulation in lung cancer. Notably, *PFKFB3* upregulation has been reported across various cancer types, including NSCLC, where it plays a central role in enhancing glycolysis, a hallmark of the Warburg effect [496, 501]. Prior evidence highlights a strong correlation between the Warburg effect and metastasis in NSCLC. In tumor cells, including those from NSCLC, glycolysis is the preferred pathway for glucose metabolism, rather than relying on mitochondrial oxidative phosphorylation. Consequently, elevated levels of *HOXD9* in NSCLC tissues enhance glycolysis and significantly contribute to tumor metastasis. Importantly, suppressing *PFKFB3* has been shown to markedly reduce the oncogenic effects of *HOXD9*, including its ability to promote metastasis [496].

The expression of *HOXD10* is notably reduced in lung cancer, demonstrating its role as a tumor suppressor in this disease. Furthermore, *HOXD10* methylation has been identified in plasma samples from patients with lung cancer. Consequently, the methylation of *HOXD10*, along with other genes such as *PAX9*, *STAG3*, and *PTPRN2*, suggests its potential utility as a diagnostic biomarker [303, 502, 503]. *HOXD10* expression is markedly reduced in NSCLC tissues and cell lines when compared with normal, noncancerous tissues. This downregulation is further influenced by miR‐224, a miRNA that is highly expressed in metastatic NSCLC tissues and the cell lines. *MiR‐224* directly targets the 3′‐UTR of *HOXD10*, leading to its suppression. Overexpression of *miR‐224* significantly reduces *HOXD10* levels, impairing its tumor‐suppressive functions and promoting cell migration and invasion in NSCLC. These findings highlight the critical interplay between *HOXD10* and *miR‐224* in lung cancer progression, although further research is needed to elucidate the precise mechanisms underlying this relationship [503]. Studies have shown that *HOXD11* exhibits inconsistent expression patterns in lung cancer, with evidence of both upregulation and downregulation depending on the lung cancer subtype. This dual profile underscores its complex role in lung cancer pathogenesis [474, 504, 505]. On the CpG island, the *HOXD11* gene has been found to be methylated in lung tumors in four out of eight cases. In contrast, DNA methylation in normal lung tissue was also observed in three out of eight cases for *HOXD11* [380]. cfDNA methylation analyses in plasma samples from lung cancer patients have identified *HOXD11*, along with genes like *BCAR1* and *HOPX*, as hypermethylated [504]. Methylation of *HOXD11* is frequently observed in the oral epithelium of lung cancer patients, particularly among smokers and individuals over 50 years of age. Combined analyses of methylation profiles have demonstrated high specificity and predictive value for assessing lung cancer risk, especially in relation to smoking and age. Aberrant *HOXD11* methylation in NSCLC is also detected to associate with resectable tumors, emphasizing its potential for early detection and risk stratification. Therefore, DNA methylation studies further emphasize the methylation of *HOXD11* as a promising biomarker [505]. Conversely, evidence has revealed significant overexpression of *HOXD11*, along with other *HOX* genes, in LUAD and LUSC tissues compared with the healthy tissue. This elevated expression is strongly correlated with poor clinical outcomes, including reduced OS and postprogression survival (PPS), further highlighting the complexity and dual role of *HOXD11* in lung cancer progression and prognosis [474].

The reported expression patterns of *HOXD13* in lung cancer vary significantly across studies, reflecting its complex role in tumor biology. Some research highlights hypermethylation of the *HOXD13* promoter, which may lead to transcriptional silencing. Conversely, other studies report increased expression of *HOXD13* in lung cancer [474, 483]. Methylation of the *HOXD13* promoter CpG island has been observed in lung tumors, reported in seven out of eight cases studied, highlighting context‐specific epigenetic silencing within the HOXD cluster. However, DNA methylation in normal lung tissue was observed in only one case [380]. Moreover, aberrant hypermethylation of *HOXD13* has been particularly observed in LUAD, suggesting its potential as a biomarker [483]. Bioinformatics analyses, including GEPIA, have demonstrated that *HOXD13*, along with other *HOX* genes such as *HOXA13*, *HOXB13*, *HOXC13*, and *HOXD11*, shows significantly higher expression in LUAD and LUSC tissues compared with the healthy tissues. Notably, this overexpression appears to persist across all stages of lung cancer and is strongly associated with a worse prognosis. The elevated expression of *HOXD13* and related *HOX* genes underscores their significant diagnostic and prognostic value, correlating with reduced OS and PPS. This makes them promising targets for further research and clinical applications in lung cancer management [474].

###### 4.3.1.1.6 Paired‐Like Homeodomain Family's Role in Lung Cancer

However, the role of PITX1 in lung development remains not fully understood, dysregulation of genes within *PITX* family has been observed in LUAD patient samples, and methylation of *PITX1* and *PITX2* has also been reported in lung cancer [45]. Despite of this, the expression profile of the *PITX* gene family has not been thoroughly explored in lung cancer, and further investigation will be required. Additionally, accumulating evidence suggests that PITX1 functions as a tumor suppressor in various cancers, including lung cancer, melanoma, and ESCC [45, 364] [506, 507]. Hypermethylation of *PITX1* gene in its promoter region has been detected in primary lung tumors and lung cancer, leading in downregulated expression. This suggesting that *PITX1* downregulation is associated with tumor progression, development, and higher stages of the tumor [364]. Studies have demonstrated that higher levels of *PITX1* are associated with decreased cell proliferation and enhanced apoptosis in melanoma cells. In ESCC, hypermethylation of the *PITX1* promoter was linked to increased tumor depth and advanced stages, indicating that reduced *PITX1* expression may contribute to aggressive cancer behavior [280]. PITX1 has been reported to inhibit the RAS signaling pathway and promote p53 activity, which are crucial in regulating cell proliferation and survival. This suggests that *PITX1* may play a pivotal role in tumor suppression by modulating these key cellular pathways [507]. In 62% of the lung cancer samples, the lack of *PITX1* expression was identified, and lower levels of expression were associated with advanced tumor stages. Despite this fact, the mechanism behind the downregulation of *PITX1* in lung cancer may not be due to its promoter hypermethylation, and further investigation will be required to determine the exact cause of downregulation [364]. Therefore, restoring *PITX1* function or targeting its regulatory pathways may offer therapeutic avenues for treating cancers where *PITX1* is silenced.

PITX2 is responsible for lungs left–right asymmetry but may not be necessary for other organs [364]. Although PITX2 involvement in carcinogenesis has yet to be precisely elucidated, the role of *PITX2* hypermethylation has been implicated in a variety of cancer types such as AML, prostate, and breast carcinoma [508, 509]. Hypermethylation of *PITX2* gene has been detected in lung SCC, with higher levels in tumor tissue compared with adjacent nontumor lung tissue [460]. Subsequent studies have revealed that methylation of *PITX2* occurred in both hypo‐ and hypermethylation in NSCLC. It is possible that hypomethylation of *PITX2* could lead to *PITX2* expression and subsequently cause the expression of *GTP‐binding protein Di‐Ras3* (*DIRAS3*) [508]. *DIRAS3*, also known as *aplysia ras homology member I* (*ARHI*), is an imprinted gene that is predominantly expressed from the paternal allele. Its downregulation has been implicated in various cancers, including lung cancer, where the loss of *DIRAS3* expression can occur through different mechanisms. Studies have shown that the re‐expression of DIRAS3 can inhibit multiple oncogenic signaling pathways, such as PI3K/AKT and RAS/MAPK, thereby blocking malignant transformation and promoting autophagy in cancer cells [510]. The evidence indicates that the DNA methylation status of *PITX2* is associated with the risk of disease. Thus, in patients with low *PITX2* methylation, the risk of disease progression was significantly higher than in patients with high methylation. Hypermethylation of *PITX2*, but not hypomethylation, has been reported to be associated with prolonged survival. As a biomarker in patients with NSCLC, the methylation of *PITX2* has been demonstrated to provide independent prognostic information regarding disease progression [508].

###### 4.3.1.1.7 Short Stature Homeobox Family's Role in Lung Cancer

Studies confirm that *SHOX2* has important roles in lung carcinogenesis. *SHOX2* hypo‐ and hypermethylation has been detected in patients with NSCLC. Hypermethylation of *SHOX2*, but not hypomethylation, has been reported to be associated with indicative of prolonged survival [508]. In 96% of tumor samples from lung cancer patients, hypermethylation of the *SHOX2* locus was found compared with that of normal adjacent tissues [47]. Accordingly, methylation of *SHOX2* was present in 70% of NSCLC, 97% of SCLC, 82% in squamous carcinoma, and 47% of adenocarcinoma [511]. The later study revealed *SHOX2* as a biomarker has been detected in 80% of SCLC and 63% of SCC, along with high sensitivity in advanced stages of cancer in comparison with the first stage [512]. In patients with low *SHOX2* methylation, the risk of disease progression was significantly higher than in patients with high *SHOX2* methylation [508]. The detection of hypermethylated *SHOX2* in bronchial aspirates could potentially provide a helpful biomarker for diagnosing lung carcinoma patients, particularly in cases with unclear results from cytological and histological examinations [511]. Despite the absence of visible tumors in the bronchoscopy, the methylation of *SHOX2* as a potential biomarker allowed the detection of malignant lung disease through blood plasma [512]. The methylation of *SHOX2* has been demonstrated to provide independent prognostic biomarkers for the progression of cancer in patients with NSCLC [508].

The 3q region of the chromosome is considered a critical region because it is frequently amplified in lung cancer. The differences between adenocarcinomas and SCC of the lungs are largely attributed to differential gene expression and 3q chromosomal copy number alterations. According to the study, SHOX2 (located at the 3q locus) exhibits higher methylation in lung SCC compared with LUAD. Therefore, it is possible to justify the connection between *SHOX2* hypermethylation and the locus amplification [508]. Accordingly, hypermethylation of the *SHOX2* gene locus has been found to be associated with frequent gene amplification, despite no differences in the expression of the *SHOX2* gene [47]. *SHOX2* hypermethylation shows promise as a biomarker in two key areas: risk stratification for the development of secondary primary lung cancer and as a prognostic indicator. It may serve as a surrogate marker for prolonged survival, particularly linked to the amplification of the 3q locus. Additionally, studies have been revealed that *SHOX2* and *PITX2* DNA methylation levels are significantly higher in SCC compared with LUAD. Hypomethylation of *PITX2* and *SHOX2* has been detected in patients with NSCLC with a high risk for tumor progression, while hypermethylation of *PITX2* and *SHOX2* has been observed in patients with low risk and is related to poor prognosis [508]. Both *SHOX2* and *PITX2* methylation could be potential predictors for tumor progression. Additionally, SHOX2 and PITX2 hypermethylation has been observed in relation with *Thyroid TF‐1* (*TTF‐1*) downregulation, which has been identified as an independent biomarker associated with an adverse prognosis in LUADs [508]. Studies have demonstrated that methylation of *RASSF1A* and *SHOX2* plays crucial roles in the tumorigenesis, progression, and metastasis in lung cancer. This makes these markers highly relevant as diagnostic biomarkers, offering high sensitivity and specificity for precise lung cancer screening [46]. In bronchoalveolar lavage fluid (BALF), both methylated *SHOX2* and *RASSF1A* were found to have high specificity and sensitivity (97.4 and 81% respectively), which can be utilized as a noninvasive method for lung cancer detection, particularly during the early stage [513]. The aberrant hypermethylation of the *RASSF1A* promoter region has been widely documented in lung cancer, occurring in approximately 63% of NSCLC cases. This distinct methylation pattern, absent in normal epithelial cells, highlights *RASSF1A* as a promising biomarker for lung cancer research. Notably, the combined analysis of *SHOX2* and RASSF1A methylation has demonstrated significant diagnostic potential, with sensitivity ranging from 71.5 to 83.2% in BALF samples. This combined methylation analysis correlates strongly with clinical parameters such as tumor size and TNM stage, providing valuable insights into disease progression. Importantly, the methylation status of *SHOX2* and RASSF1A can serve as an early indicator of tumor invasiveness, suggesting that patients testing positive for these markers may face a more aggressive clinical types of cancer [514].

###### 4.3.1.1.8 Iroquois Homeobox Family's Role in Lung Cancer

The *IRX* genes family participate in both development and differentiation as TFs, and they have been suggested to both suppress and promote several types of cancer. Specifically, *IRX1* promoter hypermethylation leads to the lack of its expression in lung cancer. Furthermore, *IRX1* promoter hypermethylation is more frequent in NSCLC than in SCLC [54]. In normal lung tissue, *IRX1* has a lesser degree of methylation in the promoter region, whereas in primary adenocarcinoma and SCC, the lack of *IRX1* expression is caused by hypermethylation of the *IRX1* gene promoter CpG sites. It is worth noting that in lung cancer, *IRX1* promoter hypermethylation occurs more often in NSCLC samples as compared with SCLC samples. It is likely that hypermethylation and downregulation of *IRX1* can be used to determine the prognosis and diagnosis of patients with adenocarcinoma [515]. *IRX2* is involved in cell migration and apoptosis, and in mouse embryos, it plays an important role in early lung development. Studies have found that the *IRX2* gene is hypermethylated at CpG sites within the promoter region in LUADs compared with normal lung tissue [516]. Furthermore, the *IRX2* gene has been detected as hypermethylated at CpG islands in lung SCC samples, compared with normal lung tissue. In Stage I lung cancer cases, *IRX2* may serve as useful biomarkers for early lung cancer diagnosis, particularly in SCC [435].

###### 4.3.1.1.9 Caudal‐Related Homeobox Family's Role in Lung Cancer

This multifaceted role of CDX2, due to its involvement in cell proliferation, differentiation, and apoptosis, makes it a key player in tumorigenesis across various cancer types. The expression and function of CDX2 have been extensively studied, particularly in the context of CRC and lung cancer. However, the role of *CDX2* can vary significantly depending on the cancer type and cellular context, acting either as a tumor suppressor or an oncogene [92, 362, 517, 518]. In the context of lung cancer, emerging evidence suggests that *CDX2* may act as a tumor suppressor by suppressing Wnt signaling, thereby preventing uncontrolled cell proliferation [362]. Interestingly, promoter hypermethylation of Wnt signaling antagonistic components has been observed to disrupt the Wnt signaling pathway in lung cancer. The re‐expression of *CDX2* has been shown to suppress cell proliferation and block cells in the G1 phase of the cell cycle by inhibiting β‐catenin/TCF activity and downstream target genes such as *c‐Myc* and *Cyclin D1*. Importantly, *CDX2* was found to be silenced in lung cancer due to its own promoter hypermethylation. In a study involving 110 primary lung cancer samples, *CDX2* was methylated in approximately 55% of cases, while it was not observed in normal lung tissue samples [362]. This suggests that *CDX2* functions as a tumor suppressor by negatively regulating Wnt signaling, thereby preventing the proliferation of lung cancer cells. Furthermore, a recent study indicated that the hypermethylation of *CDX2* in CpG islands was detected in 100% of LUAD samples, with a median level of methylation 10 times higher than that of adjacent nontumor lung tissue. This suggests that the hypermethylation of *HOXA1* and *CDX2* in CpG islands in LUAD could serve as a promising biomarker for the detection of lung cancer, particularly in early‐stage (Stage IA) tumor samples, using plasma or sputum samples [384].

###### 4.3.1.1.10 *LMX* Homeobox Family's Role in Lung Cancer

Recent studies have also uncovered tumor‐suppressive roles for several other *LHX* genes. Specifically, *LHX6*, *LHX9*, *ISL2*, and *LMX1A* have been found to exhibit anticancer functions in depends on the cancer types [519]. Research has uncovered that several members of the LMX family, including *LHX2*, *LHX3*, *LHX4*, *LHX4*, *LHX5*, *LHX6*, and *LHX9*, frequently display hypermethylation in different types of cancer tissues [90]. In lung cancer, some members of the *LHX* family have been reported to exhibit either aberrant expression or abnormal methylation, as detailed in the sections below. *LHX2* can exhibit either hypermethylation or overexpression, depending on the type of cancer. In NSCLC, *LHX2* is predominantly reported to function as an oncogene, with studies indicating its upregulation in 70% of NSCLC tissues compared with paired noncancerous samples. Therefore, evidence indicates that elevated *LHX2* expression promotes NSCLC progression by enhancing cell proliferation, migration, and invasion, largely through its regulation of the cell cycle [90, 319, 519]. *Notably, miR‐1238 levels are significantly reduced in NSCLC tissues and cells, with 62% of NSCLC tissue samples exhibiting this reduction. Analysis of patient tissue samples further reveals an inverse correlation between miR‐1238 and LHX2 expression, with simultaneous miR‐1238 downregulation and LHX2 overexpression observed in 77.4% of cases*. By targeting the 3′‐UTR of *LHX2*, *miR‐1238* directly reduces LHX2 mRNA and protein levels. Conversely, in vitro studies on lung cancer cell lines have shown that upregulation of *miR‐1238* suppresses cell viability, proliferation, migration, and invasion through downregulation of *LHX2*, highlighting its tumor‐suppressive potential. These research suggests that restoring miR‐1238 expression may provide a promising therapeutic strategy for managing NSCLC by reducing *LHX2‐*driven oncogenic activity [319]. Similarly, low levels of *miR‐124* are frequently detected in NSCLC tissues and show an inverse correlation with elevated *LHX2* expression. This upregulation of *LHX2* is associated with enhanced migratory and invasive abilities, increased metastasis, and poorer prognosis in NSCLC. Consequently, *miR‐124* downregulation exacerbates these tumorigenic phenotypes, while enhanced miR‐124 expression has been shown to suppress *LHX2* levels, reducing NSCLC cell migration and invasion. As a result, these findings underscore the tumor‐suppressive role of *miR‐124* in NSCLC progression [520]. On the other hand, aberrant methylation of the *LHX2* gene has been prominently observed in lung cancer tissues. Although low levels of methylation were detected in some normal tissues excised during tumor surgery, methylation was significantly more pronounced in tumor samples. Specifically, *LHX2* is found to be methylated in 58% of primary lung tumor cases, a frequency markedly higher than that observed in adjacent normal lung tissues. These results indicate distinct methylation patterns associated with *LHX2* in lung cancer, suggesting that its aberrant methylation may serve as an epigenetic mechanism driving tumor progression [90, 519, 521]. Similarly, *LHX4*, located on chromosome 1q25, shows significantly higher methylation levels in lung tumor tissues compared with noncancerous lung tissues. Studies indicate that 75% of primary lung tumors exhibit *LHX4* methylation, highlighting its potential role in lung cancer progression through epigenetic modifications. Specifically, low expression of *LHX4* has been correlated with an undifferentiated state of lung tumors [90, 519, 521].

*LHX3* exhibits significantly elevated expression levels in lung cancer tissues compared with adjacent noncancerous tissues. This overexpression suggests its role as a potential oncogene, particularly in NSCLC and more prominently in LUAD. Its elevated expression is closely associated with advanced clinical stages, tumor metastasis, and reduced OS, particularly in LUAD patients. Functionally, *LHX3* promotes cancer cell proliferation and invasion while inhibiting apoptosis, underscoring its oncogenic role and its designation as an unfavorable independent prognostic factor. Furthermore, *LHX3* acts as a radiosensitivity prognostic biomarker, especially in early‐stage LUAD, with higher expression levels observed in patients undergoing radiotherapy. Nevertheless, the precise molecular mechanisms underlying *LHX3*’s oncogenic activity remain poorly understood, necessitating further research [522].

*LHX6* is frequently hypermethylated at its CpG islands in lung cancer, a phenomenon observed in 56% of primary lung cancer cases. This epigenetic silencing results in a significant downregulation of *LHX6* expression in lung cancer tissues, highlighting its role as a tumor suppressor in lung carcinogenesis [523, 524]. In vitro and in vivo studies, including experiments on lung cancer cell lines and mouse models, demonstrate that enforced expression of *LHX6* markedly inhibits cell viability and tumor growth. Notably, *LHX6* expression is closely associated with inducing apoptosis, promoting G1/S cell cycle arrest, and suppressing migratory and invasive capabilities of the cancer cells. Conversely, *LHX6* downregulation facilitates cell proliferation and tumorigenesis‐related processes, emphasizing its critical role in suppressing malignancy [523, 524]. As an important TF, *LHX6* primarily mediates its tumor‐suppressive functions by negatively regulating the Wnt/β‐catenin signaling pathway. Through the silencing of *CTNNB1*, LHX6 reduces the pathway activity, leading to the suppression of four critical oncogenic downstream target genes. Therefore, it is inhibited the expression of *c‐Myc* and *Cyclin D*, key regulators of the G1 phase, thereby effectively impairing cell cycle progression. Subsequently, LHX6 diminishes tumor metastasis. Conversely, *LHX6* downregulation activates the Wnt/β‐catenin pathway, driving tumorigenesis, metastasis, and resistance to chemotherapeutic agents such as erlotinib, an EGFR‐TKI, particularly in NSCLC [523, 524, 525]. Moreover, LHX6 promotes apoptosis by upregulating the *p53* and *p21*, while simultaneously suppressing the expression of the *Bcl‐2*. These multifaceted mechanisms underscore *LHX6*’s pivotal role in inhibiting lung cancer progression and indicate its potential as a therapeutic target [523, 525, 526]. In addition, in vitro studies on NSCLC cell lines have been indicate that the upregulation of *miR‐214* is detected as a negative regulator of *LHX6* in lung cancer, directly reducing its mRNA level. This downregulation of *LHX6* activates the Wnt/β‐catenin signaling pathway, enhancing tumor cell migration and contributing to resistance against erlotinib. Therefore, restoring *LHX6* expression or inhibiting *miR‐214* expression could potentially reverse erlotinib resistance and mitigate metastatic progression in NSCLC [525, 526]. Similarly, *LHX9*, *ISL2*, and *LMX1A* have also been implicated as potential tumor suppressors, though the precise mechanisms by which they exert their inhibitory effects on cancer progression remain to be fully elucidated [519, 521]. Recent evidence indicates that epigenetic silencing of *LMX1A* in lung cancer is caused by hypermethylation of its promoter, resulting in its downregulation in NSCLC cells. Additionally, *LMX1A* exhibits tumor‐suppressive properties in NSCLC and partly prevents NSCLC cell invasion by modulating EMT, angiogenesis, and ECM remodeling [527, 528].

###### 4.3.1.1.11 *SIX* Homeobox Family's Role in Lung Cancer

Prior research has been demonstrated that aberrant expressions of the *SIX* genes can be implicated in tumor progression, tumorigenesis, and metastasis by promoting migration, angiogenesis, apoptosis, and cell proliferation [529, 530]. The *sine oculis homeobox homologue 2* (*SIX2*), as a HD‐containing gene, is located at chromosome 2. Methylation of *SIX2* has been detected in the CpG island spanning the promoter and its 3′ end in lung cancer. However, its precise role in lung cancer is still unclear and requires more extensive research [521]. There is evidence indicating that epigenetic silencing of *SIX3* in NSCLC is caused by methylation of its promoter, resulting in its downregulation in LUAD. As a consequence, in LUAD tissue, methylation of *SIX3* was downregulated compared with normal tissue adjacent to the tumor. There is evidence suggesting that *SIX3* acts as a transcriptional repressor in NSCLC by regulating relevant oncogenes, thereby preventing the phenotype of NSCLC cells [531]. However, there is limited information available about *SIX3*’s role in tumorigenesis. Besides, in patients with LUAD and bronchioalveolar carcinomas, there was an observed significant correlation between the expression of *SIX3* and improved patients’ OS rate and progression‐free survival. Consequently, *SIX3* could serve as a valid prognostic biomarker for LUAD [531]. *SIX6* gene was found to be hypermethylated in early‐stage NSCLC. In comparison with noncancerous lung tissues, SIX6 methylation is significantly higher in Stage I NSCLC. Furthermore, comethylation of SIX6 and SOX1 has been also observed in SCC and adenosquamous carcinoma samples from NSCLC patients; moreover, it is possible that their methylation contributed to the development of SCC. Its appears that comethylation of *SIX6*, *BCL2*, and *retinoic acid receptor beta* (*RARB*) might be caused by smoking. On the other hand, abnormal methylation of these three genes may be accurately used for diagnosing Stage I NSCLC due to their high‐sensitivity and specificity [49].

###### 4.3.1.1.12 Distal‐Less Family's Role in Lung Cancer

DLX1 has been found to exhibit a complex expression pattern in lung cancer, specifically in LUAD. Research indicates that both methylation of the *DLX1* gene and its upregulation are prominent features in lung cancer, particularly in LUAD [521, 532]. Despite these findings, *DLX1* mutations are rare in LUAD (1.5%) and show no correlation with LUAD patient's OS. Studies have demonstrated that *DLX1* mRNA levels are significantly elevated in LUAD tissues and cell lines compared with noncancerous controls. Notably, *DLX1* overexpression and aberrant promoter methylation in two CpG islands are strongly associated with poor OS in LUAD patients, highlighting its role as an independent prognostic factor. In particular, *DLX1* hypermethylation is believed to contribute to adverse survival outcomes in these patients. Additionally, Kaplan–Meier survival analyses reveal that elevated *DLX1* expression is significantly correlated with reduced OS, PFI, and DSS in LUAD patients. Functionally, evidence suggests that *DLX1* overexpression promotes proliferation and migration of LUAD cells. As a TF, DLX1 is involved in regulating multiple pathways, with potential links to TP53 activity and DNA replication; however, further research is needed to elucidate these associations. Furthermore, *DLX1* has been implicated in immune evasion mechanisms in LUAD cells, underscoring its multifaceted role in lung cancer progression [532].

DLX4 plays a multifaceted role in the progression of lung cancer. Notably, studies have reported both increased expression and aberrant methylation of the DLX4 gene in lung cancer tissues [190, 521, 533]. Interestingly, methylation of the DLX4 in its CpG island has been reported in more than 80% of LUAD cases [534]. Furthermore, studies on NSCLC patient samples have highlighted the methylation status of the DLX4 promoter region, revealing that 49.5% of NSCLC cases exhibit DLX4 methylation. The prevalence of methylated DLX4 is particularly high in advanced stages, with 84.6% in Stage II and 93.1% in Stage III. Similarly, in vitro studies on metastatic lung cancer cell lines demonstrated a significant downregulation of DLX4 expression, while its re‐expression was associated with an improvement in the poor prognosis of lung cancer patients. Additionally, evidence suggests that methylated DLX4 could serve as a potential biomarker for poor prognosis, particularly in Stage I NSCLC following curative resection [99, 535]. However, the results of the new studies contradict those of previous studies, not only regarding the level of DLX4 expression but also its involvement in the upregulation associated with lung cancer progression and metastasis [99, 533, 535]. Recent findings underscore the oncogenic role of DLX4 in NSCLC, particularly in regulating cell proliferation and the cell cycle. In vitro studies on NSCLC cell lines demonstrate that silencing DLX4 significantly reduces tumor cell viability and induces G1/S phase cell cycle arrest. This cell cycle arrest is characterized by a higher proportion of cells in the G1 phase and a corresponding reduction in the S phase. This arrest results in a greater number of cells being blocked in the G1 phase, coupled with a decline in the proportion of cells progressing to the S phase. Functionally, DLX4 exerts its regulatory effects through the cyclin‐dependent kinase subunit 2 (CKS2)/Y‐box binding protein 1 (YB‐1) axis, a pathway critical for NSCLC progression. DLX4 positively regulates YB‐1, a multifunctional protein essential for tumor progression, particularly in NSCLC. Research has revealed a strong correlation between DLX4 and YB‐1 expression, with DLX4 silencing directly suppressing YB‐1 levels. Silencing YB‐1, in turn, inhibits CKS2 expression, leading to reduced cell proliferation and upregulation of critical tumor suppressors genes’ expression such as phosphatase and tensin homolog deleted on chromosome 10 (PTEN), p53, p21. By modulating the CKS2/YB‐1 axis, *DLX4* drives tumor progression and highlights its central role in NSCLC growth [533].

*DLX5* has gained considerable attention for its involvement in lung cancer. Studies on various lung cancer cell lines and NSCLC tissue samples have consistently reported that *DLX5* is significantly overexpressed in cancerous tissues compared with noncancerous counterparts. Clinicopathologic analyses of NSCLC samples have further revealed that *DLX5* upregulation is strongly correlated with increased cell proliferation, tumor growth, aggressive disease phenotypes, and reduced CSS, suggesting its utility as a potential biomarker for poor prognosis. Therefore, both in vivo and in vitro studies underscore the critical role of *DLX5* as a growth factor in the development and progression of lung cancer. Functionally, DLX5 drives NSCLC progression by upregulating *MYC* transcription, a well‐established oncogene involved in promoting cell proliferation across various cancers. Silencing *DLX5* expression in lung cancer cell lines significantly reduces cell proliferation, an effect attributed to the concurrent suppression of *MYC* expression. These findings highlight the oncogenic role of *DLX5* in tumorigenesis and indicates its potential as a therapeutic target by regulating MYC‐dependent oncogenic pathways [536, 537, 538].

###### 4.3.1.1.13 Paired‐Like Homeobox Family's Role in Lung Cancer

In early‐stage of NSCLC, *PHOX2A* has been found to be hypermethylated, with significantly higher methylation rates observed in Stage I NSCLC compared with noncancerous lung diseases. This suggests its potential as a biomarker for early detection [49, 50]. In addition to its epigenetic regulation, studies on lung cancer cell lines have revealed a distinct role for *PHOX2A* in cancer progression. Increased *PHOX2A* expression has been directly linked to enhanced invasiveness in lung cancer. In vitro experiments show that enforced *PHOX2A* expression significantly alters cell cycle dynamics and apoptotic regulation, leading to a higher proportion of cells in the S phase, increased invasive potential, and reduced apoptosis rates. These effects appear to be modulated by miR‐326, demonstrating a complex regulatory network involving *PHOX2A* in lung cancer progression [539]. In vitro studies on lung cancer cell lines have identified *PHOX2A* as a direct target of miR‐326, revealing a critical relationship between their expression levels and lung cancer progression. Mechanistically, miR‐326 binds to the 3′‐UTR of *PHOX2A*, resulting in reduced PHOX2A accumulation in lung cancer cell lines. Notably, *miR‐326* levels are significantly downregulated in lung cancer; however, both in vivo and in vitro studies demonstrate that enforced *miR‐326* expression suppresses PHOX2A accumulation, inhibits cell proliferation and migration, and promotes apoptosis. Furthermore, *miR‐326* expression is negatively regulated by the *lncRNAs HOTAIR*, which suppresses its levels. In contrast, silencing *HOTAIR*, has been shown to elevate *miR‐326* expression, further supporting its tumor‐suppressive role in lung cancer [539].

###### 4.3.1.1.14 Orthodenticle Homeobox Family's Role in Lung Cancer

Numerous studies have identified *OTX1* as an oncogene, demonstrating its involvement in promoting cell proliferation, migration, and tumor progression across various cancer types. Overexpression of *OTX1* has been observed in cancers such as breast cancer, hepatocellular carcinoma, CRC, and lung cancer. While *OTX1* overexpression is frequently observed in lung cancer, its downregulation has also been reported, indicating a complex regulatory role. Additionally, *OTX1* hypermethylation at CGIs has been identified in 100% of SCC tumor samples, emphasizing its epigenetic significance in lung cancer [435, 534, 540, 541]. In NSCLC, *OTX1* is markedly upregulated in tissues and cell lines, implicating its critical role in tumor progression. Elevated *OTX1* expression is associated with poor OS in patients with NSCLC, suggesting its potential as a prognostic marker and therapeutic target. Mechanistically, in vitro studies on NSCLC cell lines reveal that *OTX1* downregulation significantly impairs cell proliferation, migration, and invasion through multiple pathways. Downregulation of *OTX1* induces G2/M phase arrest by reducing Cyclin B1 levels, which in turn suppresses the G2 to M phase transition and prevents cell growth. *OTX1* suppression also decreases p‐ERK protein levels, indicating that its tumor‐promoting effects may rely on p‐ERK activation. Furthermore, *OTX1* suppression impairs cell migration and invasion, likely by disrupting EMT through decreased expression of EMT‐related key markers such as *N‐cadherin* and *vimentin*. Finally, silencing *OTX1* promotes apoptosis, as evidenced by enhanced levels cleaved *PARP1* and active *Caspase‐3*, two key indicators of apoptotic activity. These results position *OTX1* as a multifaceted regulator of NSCLC progression, with its diverse roles in cell proliferation, migration, invasion, and apoptosis [541].

##### 4.3.1.2 Homeobox Genes Dysregulation in Other Four Cancers

Gene dysregulation of developmental regulators is a central theme in carcinogenesis, with homeobox genes emerging as pivotal modulators across diverse tumor types. Table 7 catalogues representative homeobox genes that are recurrently dysregulated in four major solid tumors breast, colorectal, prostate, and gastric cancers. Within each cancer type, the table distinguishes genes that are upregulated or downregulated and that have been functionally characterized as oncogenic drivers or tumor‐suppressive regulators. In addition, the table links these expression changes to the corresponding signaling pathways or axes and to the experimental evidence supporting their roles. Together, these data reveal both shared and tumor‑specific patterns of perturbation in developmental programs and highlight potential avenues for targeted therapy as well as candidate biomarkers for prognosis.

**TABLE 7:** Representative homeobox genes with oncogenic or tumor suppressive functions in four of the highest‐incidence solid tumors worldwide (breast, prostate, gastric, and colorectal cancers).

Cancer type	Homeobox gene(s)	Expression change in cancer (↑/↓)	Oncogene versus tumor suppressor	Signaling pathway/axis	Functional evidence/key findings	References
Breast cancer	*HOXA1*	**↑** Upregulated	Oncogene	Cell‐cycle progression; activation of NF‐κB signaling	*HOXA1* frequently upregulated in BC; overexpression drives cell proliferation and correlates with aggressive clinicopathologic features and poor prognosis, while knockdown reduces growth and induces apoptosis.	[542]
	*HOXA2*	↓ Downregulated	Tumor suppressor	Adipocytokine/PPAR signaling pathway	*HOXA2* is hypermethylated and downregulated in BC, with low expression linked to more aggressive disease and poorer survival; it positively regulates *PPARγ*, *CIDEC*/*FSP27*, and other adipocytokine/*PPAR*‐pathway genes, so loss of *HOXA2* expression disrupts this pathway in breast cancer cells.	[543]
	*HOXA5*	↓ Downregulated (promoter hypermethylation; in primary breast tumors); ↑ Upregulated (in ER^+^ cells and estradiol‐regulated; context‐dependent)	Tumor suppressor	p53 pathway	*HOXA5* shows a context‐dependent profile in BC: it is overexpressed and estradiol‐responsive in ER‐positive cells, whereas in primary tumors, its promoter methylation reduces *HOXA5* and *p53* expression; enforced *HOXA5* re‐expression restores *p53* and triggers apoptosis.	[544, 545]
	*HOXA9*	**↓** Downregulated	Tumor suppressor	EMT/Wnt/β‐cadherin pathway	*HOXA9* is frequently methylated and downregulated in BC, with its silencing linked to poorer survival and potential prognostic value; miR‐638 targets HOXA9 to inhibit EMT and Wnt/β‐catenin signaling and restrain breast cancer progression.	[546, 547]
	*HOXB7*	**↑** Upregulated	Oncogene	Activation of Ras–RAF–MAPK pathway, TGFB/SMAD3 signaling	*HOXB7* is highly expressed in BC and in a subset of TNBC; in TNBC, higher expression is paradoxically associated with weaker malignant traits, including reduced proliferative capacity, migration, and invasion.	[548]
	*HOXB9*	**↑** Upregulated	Oncogene	Activation of TGF‐β pathway; EMT; enhancement the expression of angiogenic factors	*HOXB9* is overexpressed in BC, particularly in aggressive subtypes, and activates TGF‐β signaling and EMT, upregulating angiogenic factors, thereby enhancing proliferation, neovascularization, and metastasis.	[549]
	*HOXC8*	**↓** Downregulated	Tumor suppressor	RA signaling	*HOXC8* is downregulated in BC stem/progenitor cells through promoter methylation and regulation by the miR‐196 family; its downregulation impairs retinoic acid–induced differentiation and reduces expansion of stem‐like *CD24* ^+^/*CD44* ^−^ cell populations.	[550]
	*HOXD10*	**↓** Downregulated	Tumor suppressor	miR‐10b–HOXD10–RhoC metastasis axis	*HOXD10* is frequently downregulated in invasive breast carcinoma, and reduced mRNA levels are associated with a worse histological grade. Loss of *HOXD10* enhances both metastasis and invasion. MiR‐10b suppresses the translation of *HOXD*10 mRNA, which in turn leads to increased expression of the RHOC (prometastatic gene) and is associated with breast cancer progression.	[551]
Colorectal cancer	*HOXA9*	**↑** Upregulated	Oncogene	RA signaling	Dysregulated *RA* signaling drives *HOXA9* overexpression in colorectal CSCs, leading to their abnormal expansion and contributing to CRC development and growth.	[552]
	*HOXA13*	**↑** Upregulated	Oncogene	IGF1R/PI3K/AKT/HIF1α signaling pathway	IGF1 induces *HOXA13* expression in CRC cells via an IGF1R/PI3K/AKT/HIF‐1α signaling pathway; once upregulated, HOXA13 increases *ACLY* and *IGF1R* expression, creating a positive‐feedback loop between *HOXA13* and *IGF1–IGF1R* that promotes invasion and metastasis.	[553]
	*HOXB5*	**↑** Upregulated	Oncogene	CXCR4–ERK1/2–ETS1 signaling pathway	CXCL12 upregulates *HOXB5* expression in CRC cells via a *CXCR4–ERK/ETS1* signaling cascade; once elevated, HOXB5 enhances cell migration and metastatic behaviour by increasing *CXCR4* and *ITGB3* expression, thereby creating a positive feedback loop between *HOXB5* and the *CXCL12/CXCR4* signaling that enhances metastasis.	[554]
	*HOXB7*	**↑** Upregulated	Oncogene	PI3K/AKT and MAPK/ERK signaling	*HOXB7* overexpression in CRC cells enhances proliferative capacity and coincides with stronger activity of PI3K/AKT and MAPK signaling pathway; elevated *HOXB7* drives progression from G0/G1 into S phase, accompanied by increased *cyclin D1* and reduced *p27Kip1* expression.	[555]
	*HOXB8*	**↑** Upregulated	Oncogene	STAT3 pathway	*HOXB8* overexpression enhances cell proliferation, migration, invasion, and EMT, at least probably via STAT3 activation, whereas *HOXB8* silencing reverses these malignant phenotypes.	[556]
Prostate cancer	*HOXA1*	**↑** Upregulated	Oncogene	ERK1/2 and AKT signaling	*HOXA1* is overexpressed in PCa, promoting cell proliferation, migration and invasion; conversely, knockdown of *HOXA1* suppresses ERK1/2 and AKT activation, thereby reducing tumor growth and metastasis.	[557]
	*HOXA10*	**↑** Upregulated	Oncogene	TGFβ/SMAD signaling	*HOXA10* is transcriptionally induced by *RFX6* in PCa, and its upregulation links to stronger EMT signatures, advanced tumors and recurrence; this *RFX6–HOXA10* axis amplifies *TGFβ/SMAD‐*driven EMT and contributes to enzalutamide resistance, while *RFX6* knockdown reduces *HOXA10* and restores drug sensitivity.	[558]
	*HOXA13*	**↑** Upregulated	Oncogene	HOXA13–SLC7A11/SLC3A2 axis	*HOXA13* is markedly upregulated in PCa and transcriptionally activates *SLC7A11* and *SLC3A2*, thereby inhibiting ferroptosis and promoting cell proliferation and metastasis. Conversely, *HOXA13* knockdown increases ROS, MDA and intracellular iron levels, triggering ferroptotic cell death.	[559]
	*HOXB13*	**↑** Upregulated	Oncogene	p21–RB–E2F signaling pathway	*HOXB13* is upregulated in PCa, particularly in hormone‐refractory tumors; by repressing p21 expression and activating RB–E2F signaling, it promotes androgen‐independent proliferation and survival of prostate cancer cells.	[560]
	*HOXC4*	**↑** Upregulated	Oncogene	Notch and Wnt pathways	*HOXC6* is strongly upregulated in PCa and directly modulates the expression of *BMP7*, *FGFR2*, *IGFBP3*, and *PDGFRA*, contributing to changes in Notch and Wnt signaling and thereby promoting cell proliferation.	[561]
	*HOXC8*	**↓**Downregulated	Tumor suppressor	NF‐κB signaling, miR‐196b‐5p–HOXC8–NF‐κB axis	*HOXC8* is downregulated by miR‐196b‐5p in PCa cells; by targeting the *HOXC8/NF‐κB* axis, this downregulation activates EMT and promotes metastasis.	[562]
	*DLX1*	**↑** Upregulated	Oncogene	β‐catenin/TCF4 signaling	*DLX1* is upregulated in PCa and, through binding *β‐catenin* and facilitating *β‐catenin/TCF4* complex formation, activates *β‐catenin/TCF* signaling and promotes proliferation, and migration.	[563]
Stomach (gastric) cancer	*HOXA10*	**↑** Upregulated	Oncogene	JAK1/STAT3 signaling	*HOXA10* is upregulated in GC and, by activating JAK1/STAT3 signaling, promotes cell proliferation and tumourigenesis while suppressing apoptosis. These tumour‐promoting effects are attenuated when JAK1 or STAT3 is downregulated.	[564]
	*HOXA13*	**↑** Upregulated	Oncogene	FN1‐mediated FAK/Src axis, HOXA13–FN1–FAK/Src axis; Akt/Erk1/2 activation (PI3K–Akt/MAPK, mTOR signaling)	*HOXA13* is overexpressed in GC and, by binding to the FN1 promoter, drives an FN1–FAK/Src axis that leads to phosphorylation of Akt and ERK1/2, activating PI3K–Akt and MAPK signaling pathways, thereby increasing cell proliferation and metastasis; meanwhile, miR‐449a represses *HOXA13* expression and attenuates these malignant traits.	[565]
	*HOXB7*	**↑** Upregulated	Oncogene	EGFR‐dependent pathway	*HOXB7* is overexpressed in GC, particularly in L‐OHP–resistant tumors and cell lines; it enhances cell proliferation, migration, and invasion and confers oxaliplatin resistance in an *EGFR*‐dependent manner, whereas *HOXB7* knockdown reduces proliferation and increases apoptosis, especially in cells with *L‐OHP* resistant.	[566]
	*HOXC8*	**↑** Upregulated	Oncogene	OPN‐dependent AKT/ERK signaling pathway	High *HOXC8* expression in GC is associated with increased proliferation, advanced tumor status and poor survival; *HOXC8* regulates *SPP1* expression and AKT/ERK phosphorylation, activating an OPN–AKT/ERK signaling axis, whereas *HOXC8* knockdown suppresses *SPP1* expression, decreases cell viability and weakens this signaling.	[567]
	*HOXC12*	**↑** Upregulated	Oncogene	Wnt/β‐catenin signaling pathway	High *HOXC12* expression enhances migration and invasion by promoting *SALL4* expression and *SALL4‐*dependent activation of Wnt/β‐catenin signaling and is accompanied by reduced CD8^+^ T‐cell infiltration.	[568]
	*HOXD9*	**↑** Upregulated	Oncogene	HOXD9–RUFY3 axis	High expression of *HOXD9* in GC enhances *RUFY3* transcription; its upregulation drives GC cell proliferation and migration, and is associated with poor survival, whereas *RUFY3* knockdown suppresses these *HOXD9*‐dependent malignant phenotypes.	[569]
	*CDX2*	**↑** Upregulated	Oncogene	Reg IV/SOX9 signaling	*CDX2* overexpression in GC cell lines enhances migration and invasion, in part through increased *Reg IV* expression, which in turn elevates *SOX9* expression and promotes a *Reg IV–SOX9* signaling axis linked to invasive behavior.	[570]

Abbreviations: Bcl‐2: B‐cell lymphoma 2; *BMP7*: *Bone morphogenetic protein 7*; CRC: colorectal cancer; CSCs: colonic cancer stem cell; *CXCL12*: *C‐X‐C motif chemokine ligand 12*; *CXCR4*: C*‐X‐C motif chemokine receptor 4*; *ERK*: *Extracellular regulated protein kinase*; *ETS1*: *ETS proto‐oncogene 1, transcription factor*; *FGFR2*: *Fibroblast growth factor receptor 2*; GC: gastric cancer; *IGF1*: *Insulin‐like growth factor 1*; *IGFBP3*: *Insulin‐like growth factor‐binding protein 3*; *ITGB3*: *Integrin subunit beta 3*; *L‐OHP*: *Oxaliplatin*; MDA: malondialdehyde; OPN: osteopontin; PCa: prostate cancer; *PDGFRA*: *Platelet‐derived growth factor receptor alpha*; RA: retinoic acid signaling; *RFX6*: *Regulatory factor X, 6*; *RUFY3*: *RUN and FYVE domain containing 3*; *SALL4*: *Spalt‐like transcription factor 4*; *SPP1*: *Secreted phosphoprotein 1*; *STAT3*: *Signal transducer and activator of transcription 3*; TNBC: triple‐negative breast cancer cells.

## 5 Clinical and Translational Implications of Homeobox Genes in Cancer Diseases

Given the broad involvement of homeobox genes across multiple cancer hallmarks and tumor types, there is growing interest in therapies that act through multiple, complementary strategies. Importantly, the oncogenic or tumor‐suppressive functions of homeobox gene dysregulation are not determined by altered expression alone but emerge from a broader regulatory network that includes DNA‐binding cofactors, intersecting signaling pathways, and multiple layers of ncRNA regulation such as miRNAs that directly target *HOX* transcripts [570, 571, 572, 573]. Pan‐cancer analyses further suggest that homeobox expression patterns are closely linked to features of the TME, which influence prognosis and may modulate responses to immunotherapy [17]. Moreover, in many malignancies, dysregulated oncogenic members of the homeobox family drive tumor initiation and progression while also contributing to intrinsic and acquired resistance to systemic therapy. For example, overexpression of *HOXA5*, *HOXB7*, and *HOXB5* in estrogen receptor‐positive breast cancer promotes resistance to tamoxifen by activating related signaling networks [574, 575, 576]. Consequently, a wide range of therapeutic approaches has been proposed or developed to target homeobox complexes directly or to modulate this extended regulatory network.

Therapeutic strategies under investigation for cancers with dysregulated *HOX* genes span direct targeting of HOX complexes and modulation of their upstream and downstream networks. Notable approaches include disruption of HOX/PBX dimers using peptide inhibitors such as HXR9, which demonstrates antitumor activity in vivo across cancer types including PCa and NSCLC [573]. Epigenetic‐related strategies, including histone deacetylase (HDAC) inhibitors, have been computationally prioritized as compounds correlated with *HOX* downregulation and possessing antitumor properties [17]. Small‐molecule DNA ligands that disrupt *HOXA9/DNA* binding, such as *DB1055* and *DB818*, have shown efficacy in suppressing proliferation and inducing cell death in *HOXA9*‐dependent *AML* models. RNA‐based approaches namely *siRNA* or *shRNA*‐mediated knockdown of oncogenic *HOX* genes also show promise; for example, targeted suppression of *HOXD3* in CRC cell lines using *siRNA* and *HOXD3*‐specific shRNA delivered by lentiviral vectors led to marked reductions in *HOXD3* transcripts in RKO cells, accompanied by impaired cell growth and increased apoptosis [577]. Homeobox genes are increasingly recognized as multilayered cancer biomarkers that provide diagnostically, prognostically, and therapeutically relevant information across biological levels. They can serve as diagnostic biomarkers by helping to distinguish tumor tissue from normal tissue or from benign disease. A prominent example is bladder cancer, where DNA methylation‐based urinary biomarkers improve risk stratification at diagnosis. Panels measuring methylation of genes such as *HOXA9*, *POU4F2*, *ONECUT2*, and *PCDH17* in urine can differentiate bladder cancer from nonmalignant urological conditions with high predictive value, enabling many genuinely low‐risk patients to avoid invasive follow‐up examinations [578].

## 6 Discussion

Considering that homeobox genes are implicated in both malignant and noncancerous disorders, current evidence indicates that dysregulation of these genes is described far more extensively in cancers than in noncancer conditions. This imbalance reflects the central role of homeobox TFs, including both HOX clusters and non‐HOX families, in key cancer hallmarks such as proliferation, survival, EMT, invasion, and metastasis. Across numerous studies, HOX family members are frequently dysregulated either overexpressed with oncogenic activity or silenced with LOF of tumor‐suppressive effects across a wide range of tumors. In aggregate, dysregulated expression of homeobox genes across tumor types has repeatedly been linked to more aggressive clinicopathological features and poorer outcomes [3, 18, 187]. Systematic comparisons of five of the most common solid cancers illustrate how frequently homeobox alterations recur across distinct histological subtypes and molecular contexts. In lung cancer, one of the most prevalent solid tumors, large‐scale genomic and epigenomic analyses have identified extensive sets of aberrantly expressed and epigenetically deregulated genes; genome‐wide DNA methylation profiling shows that a broad spectrum of loci become hyper‐ or hypomethylated and that numerous genes exhibit altered expression across the major histological subtypes [3, 301].

Aberrant expression of homeobox genes plays a pivotal role in the pathogenesis of lung cancer through various mechanisms such as epigenetic modifications, and also regulatory interactions with miRNAs and lncRNAs [2, 190, 579]. Alterations in gene expression are integral to the onset and progression of lung cancer, with many such changes being driven by epigenetic mechanisms. Among these, abnormal DNA methylation has emerged as a critical factor in lung cancer development. For instance, hypermethylation of promoter regions in essential tumor suppressor genes often results in their transcriptional silencing, thereby facilitating tumor growth, invasion, and metastasis [190]. Any alteration in gene expression plays a critical role in the onset and progression of lung cancer. Numerous gene expression changes have been identified in this cancer, many of which are regulated by epigenetic modifications. Among these, abnormal DNA methylation has gained recognition as a critical factor in lung cancer development. For example, hypermethylation of promoter regions in essential tumor suppressor genes often leads to their transcriptional inactivation, thereby promoting tumor growth and metastasis. As outlined in our previous study, such epigenetic modifications in lung cancer frequently correlate with increased metastasis and malignancy [301]. Beyond methylation, the mis‐regulation of specific genes is closely tied to disruptions in crucial signaling pathways, such as the Wnt pathway, which is integral to cell proliferation and differentiation. Additionally, the homeobox genes superfamily, a well‐known group of TFs fundamental to developmental processes, warrants particular attention in this context. This superfamily comprises a broad array of genes with context‐specific expression patterns across various cancer types [580]. According to experiments conducted on lung cancer, HOX genes clusters exhibit different expression patterns across various types of cancer, especially lung cancer [1]. In this way, HOXA‐ and HOXB‐related genes from the 3′ end expression mostly observe in healthy adult lung tissues [381]. Moreover, the majority of homeobox genes are not expressed in adult tissues; therefore, their absence contributes to de novo methylation during malignancy progression [581]. As a consequence, aberrant expression of HOX gene clusters is a defining feature, with distinct gene families within the superfamily exhibiting unique expression profiles depending on the tumor type in lung cancer. This variability highlights the multifaceted roles of these genes in cancer biology, characterized by their remarkable complexity and duality. While some studies suggest an oncogenic role for certain *HOX* genes, others point toward their tumor‐suppressive properties [582]. Therefore, in human lung tissue, HOX genes have distinct patterns of expression [373]. In particular, depending on the type of lung cancer, these expression levels can vary, either upregulation or downregulation.

This indicates the complex and context‐dependent nature of *HOX* genes function in cancer. Understanding the intricate regulation of these genes could provide valuable insights into cancer mechanisms and pave the way for novel therapeutic approaches. Unraveling the complex interplay of HOX genes in cancer is also essential to develop more effective and targeted therapeutic strategies. Deciphering the precise role of individual HOX genes, and how they interact with other cellular pathways, will be crucial in leveraging their potential for cancer treatment. A comprehensive understanding of the *HOX* genes network in different solid tumor types may open up new avenues for developing more personalized and precise interventions [582]. The relationship between DNA methylation and homeobox gene expression is multifaceted and complicated, with significant implications for understanding tumor biology. Both hypermethylation and hypomethylation can influence the behavior of these critical genes, but in differing ways across various cancer types. In some instances, hypermethylation of homeobox genes has been associated with their transcriptional silencing and a potential tumor suppressor role. This epigenetic modification can lead to the downregulation of these genes, which may otherwise function to inhibit tumor development and progression. Conversely, hypomethylation of homeobox genes has been observed to result in their overexpression, potentially contributing to an oncogenic phenotype in certain cancers. This dysregulation of the normal expression patterns of these developmentally important genes can disrupt cellular homeostasis and promote malignant transformations [191, 583]. Aberrant DNA methylation patterns across different cancer types can alter *HOX* genes’ expression, which is a key factor in tumorigenesis and can serve as a biomarker for cancer prognosis and treatment [303, 407]. According to previous reports, several homeobox genes are abnormally methylated in the lung cancer cell lines [2]. Despite most of the CGIs in *HOX* gene clusters being highly methylated in lung cancer cells and primary lung tumors, some of the CGIs are still nonmethylated. Therefore, in the gene‐rich *HOX* cluster, both of these regions can occur adjacent to each other [2]. The studies evidence suggests that methylation of *HOX* genes is specifically tied to various types of cancer and may even be related to normal development and tissue‐specific differentiation [584]. Accordingly, *HOX* genes can provide a better understanding of some types of human diseases when considered in relation to cancers [82]. For instance, in 97 samples from NSCLC patients, *HOXA2* (78%), *HOXA10* (40%), and *SHOX2* (39%) were found to be among the methylated genes [394]. Studies have previously reported that the *HOXA* and *HOXD* clusters substantially contain high levels of methylation, while the clusters *HOXB* and *HOXC* have a lower proportion of methylation in the lung SCC [585]. For instance, studies have reported hypermethylation of some *HOX* genes, such as *HOXA3* and *HOXD10*, exhibit hypermethylation across a wide range of 16 cancer types. Additionally, *HOXA9* and *HOXB13* have been found to be hypermethylated in solid tumors, while *HOXA* genes are frequently hypermethylated in breast cancer [1, 191].

On the other hand, a comparison between normal and cancerous lung tissue revealed that several *HOX* genes from different clusters, such as *HOXA1* [385], *HOXA10* [428], *HOXB7* [586], *HOXB8* [69], and *HOXC6* [587], *HOXC13* [588] and HOXD9 [496], are highly expressed in lung cancer tissues [585]. Evidence suggests that the increased expression of certain *HOX* genes across various clusters is strongly associated with their oncogenic potential. Abnormal *HOX* genes’ expression may promote oncogenesis by activating antiapoptotic pathways, which contribute to the survival and proliferation of cancer cells. Consequently, it is postulated that *HOX* gene involvement in suppressing apoptosis plays a critical role in cancer progression, promoting malignancy through mechanisms such as cell proliferation, survival, migration, and invasion [150, 579]. Conversely, some *HOX* genes with downregulated expression are suggested to act as tumor suppressors, highlighting the dual roles of these genes in cancer biology. During *HOX* genes hypermethylation, the activity of tumor‐suppressor and/or apoptotic *HOX* genes is silenced, which contributes to tumorigenesis in multiple types of cancer [303]. Notable example is *HOXA5*, which has been identified as a tumor suppressor in NSCLC. *HOXA5* demonstrates tumor‐suppressive roles by regulating cytoskeletal remodeling and inhibiting metastasis. Research indicates that the downregulation of *HOXA5* expression, often due to promoter hypermethylation, is associated with NSCLC pathogenesis and poor prognosis. Conversely, its upregulation can inhibit cell proliferation by regulating the expression of *p21* [399, 401, 402]. Conversely, other genes within the same cluster, such as *HOXA10* and *HOXA13*, are frequently upregulated in lung cancer. *HOXA10*, particularly in LUAD, is implicated in promoting tumor progression and metastasis. As an oncogene, *HOXA13* enhances tumorigenicity and metastasis through its regulation of the p53 and Wnt/β‐catenin pathways [351, 589, 590]. A similar duality in expression is observed in the *HOXB* cluster, where most genes are reported to be dysregulated in lung cancer, particularly in LUAD tumor tissue samples. These include *HOXB2*, *HOXB3*, *HOXB4*, *HOXB6*, *HOXB7*, *HOXB8*, *HOXB9*, and *HOXB13* [303, 396, 438]. Among them, *HOXB3*, *HOXB7*, *HOXB8*, and *HOXB9* are consistently upregulated in LUAD and are strongly associated with lung cancer pathogenesis. Specifically, the upregulation of *HOXB9* and *HOXB3* expression are correlated with poor cancer progression in patients with LUAD [69, 438, 443, 447]. In addition, several genes within the *HOXC* clusters, such as *HOXC4*, *HOXC6*, *HOXC8*, *HOXC9*, and *HOXC10*, are upregulated in lung cancer. Most of these upregulated genes predominantly exhibit oncogenic roles and are directly involved in tumorigenesis [379, 450, 452, 466]. For example, the oncogene *HOXC8* has been studied for its critical role in promoting the lung tumor progression. Research demonstrates that HOXC8 enhances the proliferation and migration of lung cancer cells by upregulating TGFβ1, a key factor involved in tumor development and progression [379]. However, both downregulation and upregulation of genes’ expression in *HOXD* cluster, such as *HOXD3* [483, 485], *HOXD8* [436], and *HOXD13* [474, 483], are reported. For instance, *HOXD8* and *HOXD9* exhibit elevated expression levels in NSCLC, suggesting their potential involvement in tumor development. Interestingly, their expression levels are associated with metastasis in NSCLC [491, 496]. In contrast, *HOXD10* is downregulated in NSCLC, due to promoter methylation, indicating that it may have a distinct or opposing function, potentially [502, 522]. Consequently, compared with normal lung tumors, primary NSCLC exhibits dramatically higher transcription of most *HOXC* and *HOXD* cluster‐related genes [372, 399]. Furthermore, both of these clusters expressed in fetal lungs as well [381].

Notably, this review also highlights the dysregulation of gene expression in various homeobox families beyond the *HOX* clusters. Significant examples include genes within the *IRX*, *SHOX*, *PITX*, *CDX*, *SIX*, *LHX*, *OTX*, and *DLX* families. The *LHX* family, in particular, exhibits both upregulation and downregulation across different genes. For instance, *LHX2* is hypermethylated, leading to its downregulation, whereas *LHX3* is frequently upregulated [521]. It is important to emphasize that duality in expression patterns is a recurring theme across homeobox genes in lung cancer [3, 521]. Some studies report downregulation of specific genes in certain subtypes, while others observe upregulation in different contexts. Notable examples of this phenomenon include *LHX2* [520], *DLX4* [534], and *HOXD13* [483], whose expression levels vary depending on the lung cancer subtype. This variability underscores the complex and context‐dependent roles of homeobox genes in lung cancer pathogenesis [3, 521]. In addition, the overexpression of *LHX2* may function as an oncogenic driver, highlighting the complex role of *LHX* family genes, where individual members can either promote or inhibit tumorigenesis depending on their expression context. Given the altered expression patterns of *LHX* genes, such as *LHX2* and *LHX4*, in cancerous tissues, these genes are being investigated as potential biomarkers for the diagnosis and prognosis of lung cancer. Their expression levels could offer valuable insights into tumor behavior and patient outcomes [90, 519]. As mentioned earlier, *HOX* genes are intricately linked with several oncogenic pathways in lung cancer. Dysregulation of homeobox gene expression is influenced by key signaling pathways, including the Wnt/β‐catenin, PI3K/AKT, and TGF‐β/SMAD3 pathways, all of which are known to be altered in various types of cancer, including lung cancer [377]. For instance, activation of the Wnt pathway has been associated with aberrant expression of specific *HOX* genes, contributing to tumorigenesis [301]. Similarly, upregulation of *HOXB7* has been shown to promote activation of the TGF‐β/SMAD3 signaling pathway, directly driving tumor metastasis in LUAD patients [377]. These interconnected pathways underscore the multifaceted role of *HOX* genes in lung cancer and highlight their potential as targets for combinatorial therapeutic approaches.

LncRNAs and miRNAs are known to regulate the expression of *HOX* genes in lung cancer. Studies have revealed a complex interplay between lncRNAs, miRNAs, and *HOX* genes, demonstrating how they can influence homeobox gene regulatory networks directly or indirectly. Notably, lncRNAs and miRNAs are implicated in metastasis and drug resistance, underscoring their potential as novel therapeutic targets in lung cancer [329, 330, 591, 592]. Additionally, the interaction between lncRNAs and DNA methylation plays a crucial role in shaping the epigenetic landscape of lung cancer [326, 327]. Given their significant impact on lung cancer pathogenesis, further investigation into their specific roles in tumorigenesis is essential. In lung cancer, *lncRNAs* can function as either oncogenes or tumor suppressors, further complicating their roles in the disease. Several *lncRNAs* and also *lncRNAs* associated with *HOX* genes, such as *HOXA*‐*AS2* and *HOTAIR*, are upregulated in lung cancer and are involved in cancer‐related processes. *LncRNAs* can also interact with their target genes by binding to related TFs, thereby repressing or promoting the transcription of these genes. Moreover, *lncRNAs* can act as ceRNAs, interacting with *miRNAs*. Consequently, *lncRNAs* regulate homeobox gene expression through multiple pathways, highlighting their critical role in lung cancer [329, 593]. Similarly, miRNAs can function as either oncogenic miRNAs (oncomiRs) or tumor suppressors, depending on their function and the specific target genes they regulate [594, 595, 596]. This duality is evident in lung cancer, where altered miRNA expression contributes to both tumor initiation and progression. For example, *HOXA5* is a target gene of miR‐196a and miR‐891a‐5p in NSCLC, and both *miRNAs* have been identified as oncogenes in NSCLC [400, 593, 597]. Additionally, *HOXA5*, acting as a tumor suppressor, is also downregulated by *HOTAIR*, which is directly linked to increased cell migration and tumorigenesis in NSCLC. Interestingly, studies have shown that HOXA5, as an important TFs, can bind to the promoter of lncRNA *LINC00312*, suppressing NSCLC cell proliferation. However, *LINC00312* levels are reported to decrease in the plasma of NSCLC patients [598]. Targeting *lncRNAs* and *miRNAs* in lung cancer has garnered significant attention as a potential therapeutic strategy. Notably, specific regulatory axes identified in lung cancer, such as the *LINC00472*/*miR‐1275*/*HOXA2* axis, *HCP5*/*miR‐17‐5p*/*HOXA7* axis, and *LINC00466*/*miR‐144*/*HOXA10* axis, have been extensively studied due to the intricate regulatory networks of *lncRNAs* and *miRNAs* [395, 404, 426]. These discoveries underscore the potential of targeting these molecular pathways for the development of innovative treatment strategies.

## Author Contributions

M.D. and S.R. conducted all literature searches relevant to the article's topic and contributed to drafting and writing the manuscript. Mo.D. and S.Y. assisted with data collection. M.A. and Sh.F. provided grammar and language editing of the final manuscript. S.R. and M.D. created all figures and finalized and edited the manuscript. S.R. is the corresponding author. All authors reviewed and approved the final version and confirm their agreement with its content and readiness for publication.

## Conflicts of Interest

The authors declare no conflicts of interest.

## Ethics Statement

The authors have nothing to report.

## Data Availability

The authors have nothing to report.
